# Unraveling Charge and Energy Transfer in a Singlet Fission Donor–Acceptor Complex: An *Ab Initio* Quantum Dynamical Study

**DOI:** 10.1021/acs.jctc.5c01945

**Published:** 2026-02-25

**Authors:** Karin S. Thalmann, Pedro B. Coto, Michael Thoss

**Affiliations:** † Institute of Physics, University of Freiburg, Hermann-Herder-Str. 3, 79104 Freiburg, Germany; ‡ Nanomaterials and Nanotechnology Research Center (CINN), 203373 CSIC-University of Oviedo-Principality of Asturias and Donostia International Physics Center (DIPC), Avda. de la Vega 4-6, 33940 El Entrego, Spain

## Abstract

Singlet fission is a photophysical process in organic molecules that generates two triplet electronic states from an excited singlet electronic state. Molecules exhibiting singlet fission can multiply charge carriers and thus have the potential to enhance the performance of solar cells beyond the Shockley–Queisser limit by reducing thermalization losses. However, in order to implement singlet fission for applications in photovoltaics, it is essential to understand how charge or energy can be harvested from triplet excitons. In this work, we investigate these processes in a prototypical donor–acceptor complex consisting of a bis­(diazadiborine)-based chromophore as a singlet fission-active donor and tetracyanoquinodimethane as an acceptor molecule. Using a combined approach of high-level *ab initio* multireference perturbation theory techniques and quantum dynamical simulations, we show the existence of intermolecular singlet fission, charge and energy transfer following intramolecular singlet fission, and energy loss decay channels to low-lying states as the three competing charge and energy transfer mechanisms from the donor to the acceptor molecule. We analyze the role of the different electronic states, specific vibrational modes, and vibronic couplings in these processes. The results provide insights into the rational design of donor–acceptor systems with efficient singlet fission-based charge and energy transfer.

## Graphical abstract

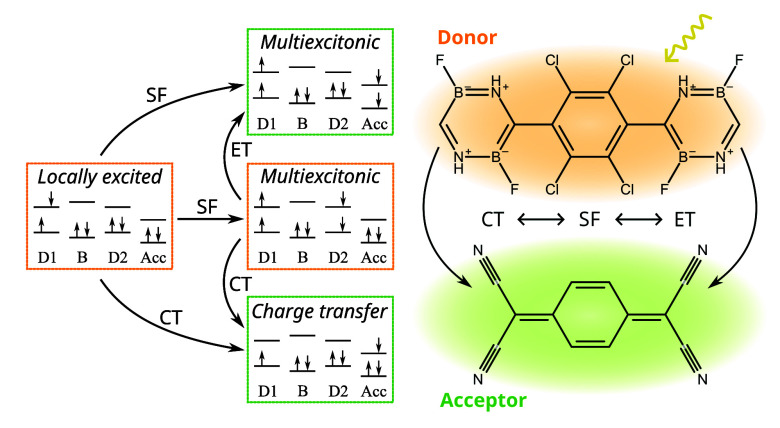

## 1 Introduction

Silicon-based solar cells are the most mature photovoltaic technology currently in operation, exhibiting good energy conversion efficiencies, low degradation rates, and low production costs. However, despite improvements in design and fabrication, the power conversion efficiency of conventional single-junction silicon-based solar cells is limited by thermalization processes, causing energy loss of hot charge carriers.^1^ Preventing this energy loss would enable overcoming the theoretical Shockley–Queisser efficiency limit in a single-junction solar cell.^2^

In this context, singlet fission (SF)^3,4^ has been proposed as a potential candidate to overcome thermalization losses by multiple exciton generation.^5–11^ SF is a spin-allowed process that transforms a high-energy photoexcited singlet electronic state S_1_ to two low-energy triplet states T_1_ mediated by a multiexcitonic state ^1^(TT):^3,4,12,13^

1 S0S1↔(TT)1↔T1+T1

The possibility of extracting energy or multiple charge carriers from the low-energy T_1_ excitons makes materials exhibiting SF potential candidates for the design of next-generation solar cells with improved power conversion efficiencies.^14,15^ This has motivated a multidisciplinary research area devoted to identifying key structural features and energetic properties of materials showing efficient SF^3,16–24^ and to characterizing the mechanism and dynamics of the process.^12,25–37^ As a result, a number of compounds exhibiting SF have been identified. These include different classes of organic materials, such as polycyclic aromatic hydrocarbons,^27,32,38–41^ pyrenes,^42^ diradicals,^16,43,44^ and different structures such as molecular dimers,^19,45–50^ donor–acceptor copolymers,^17,22,34,51–57^ conjugated polymers^20,58,59^ and molecular crystals.^9,40,46^

Concerning the nature of the SF process, two main mechanisms have been proposed: direct and mediated. The direct mechanism proceeds through the population of the ^1^(TT) multiexcitonic (ME) state directly from the S_0_S_1_ locally excited (LE) state.^28^ The mediated mechanism, on the other hand, involves the participation of high-energy charge transfer (CT) or doubly excited (DE) states.^8,29,37,47,60^ In both mechanisms, the ME state ultimately splits into two T_1_ states.^35,61,62^

To find ways for the successful implementation of SF in energy conversion devices, the charges or energy generated by using SF must be extracted. For this, two main aspects must be addressed. The first step is the selection of an appropriate acceptor material. In this respect, several molecules, including chloranil, fullerene, and tetracyanoquinodimethane (TCNQ), have been identified as suitable acceptor candidates based on their electrochemical properties.^15,63–67^ The second aspect is the investigation of the conditions that enable efficient charge and/or energy transfer from the SF-active molecule to the acceptor. This involves the characterization of the electronic states involved in the processes, their specific roles and vibronic couplings, the identification of competing processes, and the analysis of the dynamics, including the vibrational degrees of freedom. The theoretical investigation of these properties, however, is challenging due to the complexity of the problem and the resulting high computational cost involved.^30,68,69^

In this work, we tackle this problem and analyze the complex interplay between SF and charge and energy transfer processes. For our analysis, we consider an SF-active donor–acceptor model complex with a bis­(diazadiborine)-based chromophore (DADB)^70^ as the SF-active donor and TCNQ as the electron acceptor molecule (see Figure 1), and perform quantum dynamical simulations. Using vibronic model Hamiltonians, we reveal competing intermolecular charge and energy transfer processes and assess the role of the different electronic states involved. Furthermore, we show the importance of vibronic coupling and study the influence of vibrational modes on the dynamics to provide insights into the design of future donor–acceptor systems exhibiting efficient SF-based charge and energy transfer.

**Figure 1:**
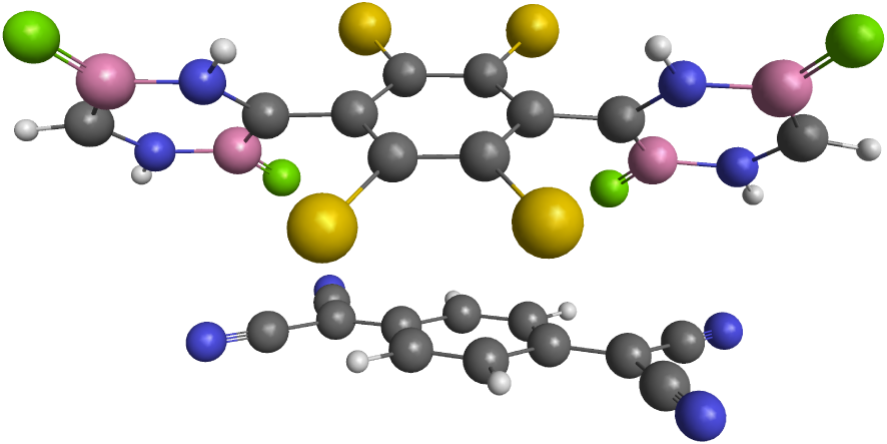
Ground-state equilibrium structure of the donor–acceptor complex with the bis­(diazadiborine)-based chromophore as the donor (D) at the top and tetracyanoquinodimethane as the acceptor (Acc) molecule at the bottom. The H, B, C, N, F, and Cl atoms are described by the white, pink, gray, blue, green, and yellow spheres, respectively.

## 2 Method

We use a combined approach of high-level *ab initio* electronic structure methods and quantum dynamical simulations.^47^ In detail, the time-dependent Schrödinger equation is solved for a vibronic coupling model Hamiltonian in the diabatic basis using the multilayer formulation of the multiconfiguration time-dependent Hartree (ML-MCTDH) method.^71–76^ In the harmonic approximation, the employed vibronic model Hamiltonian is described by

2 $$Ĥ=\underset{I}{\sum }{Ĥ}_{II}\left|I\right\rangle \left\langle I\right|+\underset{I>J}{\sum }\left({\mathrm{V̂}}_{IJ}\left|I\right\rangle \langle J|+h.c.\right)$$

with the diagonal terms

3 $${Ĥ}_{II}=E_{II}+\underset{m}{\sum }\frac{1}{2}\omega_{m}\left({\mathrm{p̂}}_{m}^{2}+{\mathrm{q̂}}_{m}^{2}\right)+\underset{m}{\sum }\kappa_{II}^{m}{\mathrm{q̂}}_{m}$$

and the off-diagonal terms

4 $${\mathrm{V̂}}_{IJ}=E_{IJ}+\underset{m}{\sum }\kappa_{IJ}^{m}{\mathrm{q̂}}_{m}$$

In the equations above, we have used units with *ℏ* = 1. The indices *I* and *J* denote the diabatic states with energies of *E*_*II*_ and electronic interstate couplings of *E*_*IJ*_. The position and momentum operators are described by *q̂* and *p̂,* respectively, and the frequency of vibrational mode *m* is denoted by ω_*m*_. The term $\frac{1}{2}\omega_{m}\left({\mathrm{p̂}}_{m}^{2}+{\mathrm{q̂}}_{m}^{2}\right)$ thus describes the kinetic and potential energy of the vibrational mode *m*. The linear vibronic coupling constant is denoted by κ. In the calculations reported below, modes were included if the dimensionless reorganization energy fulfills κ^2^/(2ω^2^) > 0.275 and the frequency ω_*m*_ > 100 cm^–1^.

To determine the parameters of the vibronic model Hamiltonian of the donor–acceptor complex, the ground-state equilibrium structure and the vibrational modes of the dimer were obtained using density functional theory (DFT), employing the B3LYP exchange-correlation functional,^77^ Grimme’s D3 dispersion interaction correction,^78^ C_1_ symmetry, and the 6–31+G­(d) basis set.^79–87^ The resulting ground-state equilibrium geometry of the complex is asymmetric, while the isolated DADB molecule is of C_2_ symmetry, and TCNQ is of D_2h_ symmetry. The 19 lowest-lying adiabatic singlet electronic states were calculated using state-averaged complete active space self-consistent field (CASSCF) calculations with an active space of 8 active electrons and 8 active orbitals, and the cc-pVDZ basis set.^88^ To keep the calculations computationally feasible, the number of electronic states was optimized while ensuring the state characters accurately represent our model system. Also, the active space was kept at 6 active orbitals localized on DADB, similar to the work of Zeng,^70^ with two additional orbitals localized in TCNQ. To account for the dynamic correlation effects, the extended multiconfiguration quasi-degenerate perturbation theory (XMCQDPT)^89^ at second order was employed. To mitigate the effects of intruder states, an intruder state avoidance shift of 0.02 (au)^2^ was used. These adiabatic states were used for the construction of the corresponding diabatic states in Nakamura and Truhlar’s fourfold way.^90–92^ The linear vibronic couplings in the diabatic model Hamiltonian were calculated from the diabatic potential energy surfaces by using a linear fitting function. The ground-state equilibrium structure was calculated at the DFT level, while the diabatic potential energy surfaces are based on the high-level XMCQDPT. This results in a difference in the diabatic ground-state equilibrium structure. To correct this difference, the diabatic Hamiltonian was shifted to the ground-state equilibrium structure at the XMCQDPT level by using a coordinate transformation.

To analyze the charge and energy transfer processes, we consider the population *P*_*I*_(*t*) of the different states, which is defined by

5 $$P_{I}\left(t\right)=\mathrm{Tr}\left\{\left|I\right\rangle \langle I|\mathrm{\rho ̂}\left(t\right)\right\}$$

where the density matrix *ρ̂*(*t*) is given by

6 $$\mathrm{\rho ̂}\left(t\right)=e^{-iĤt}\mathrm{\rho ̂}\left(0\right)e^{iĤt}$$

For a clearer understanding of the processes, we further analyze the state population sums *P*_{*I*}_(*t*) of subsets of diabatic electronic states *I* with similar character using

7 $$P_{\{I\}}\left(t\right)=\underset{\left\{I\right\}}{\sum }P_{I}\left(t\right)$$

In our simulations, we assume an instantaneous photoexcitation resulting in the initial state

8 $$\mathrm{\rho ̂}\left(0\right)=\left|\Psi_{0}\right\rangle \left|0_{0}\right\rangle \left\langle 0_{0}\right|\left\langle \Psi_{0}\right|$$

with |Ψ_0_⟩ denoting the initial diabatic electronic state and |0_0_⟩ the vibrational ground state of the electronic ground state. The initially excited electronic state is one of the diabatic LE states in the donor molecule.

All electronic structure calculations needed to parametrize the vibronic model Hamiltonian were carried out using the Turbomole V7.5 package^93^ and GAMESS 2020 R2.^94^ The quantum dynamical simulations were carried out using the Heidelberg MCTDH package.^95^ Further computational details are provided in the Supporting Information (SI).

## 3 Results and Discussion

In the following, we present the results of the simulation of the SF, charge transfer, and energy transfer processes of the DADB-TCNQ dimer, obtained using the methods outlined above.

### 3.1 Electronic Structure of the Donor–Acceptor Complex

In this section, we discuss the electronic structure of the donor–acceptor complex, including the diabatic states, their energies, and electronic interstate couplings.

The diabatic electronic states were obtained from the corresponding adiabatic states using the fourfold way method^90–92^ (see SI for details). Figure 2 shows the diabatic molecular orbitals (DMOs) used for the construction of the electronic diabatic states. These orbitals are π-like and localized on the different structural moieties involved in the SF, charge transfer, and energy transfer processes. Specifically, the DMOs of the donor moiety include two classes of π-π* orbital pairs. One class ( $\pi_{D1}-\pi_{D1}^{*}$ and $\pi_{D2}-\pi_{D2}^{*}$ ) is localized on the left (D1) and on the right (D2) diazadiborine ring. The other class $\left(\pi_{B}-\pi_{B}^{*}\right)$ is localized on the tetrachlorophenylene bridge (B) moiety linking the diazadiborine rings. The orbitals have been selected to account for the intramolecular SF process involving the diazadiborine rings and to incorporate the effects of the molecular linker on the SF.^70^ The remaining pair of DMOs $\left(\pi_{\mathrm{Acc}}-\pi_{\mathrm{Acc}}^{*}\right)$ is localized on the TCNQ molecule and has been selected to account for the donor–acceptor charge and energy transfer processes.

**Figure 2:**
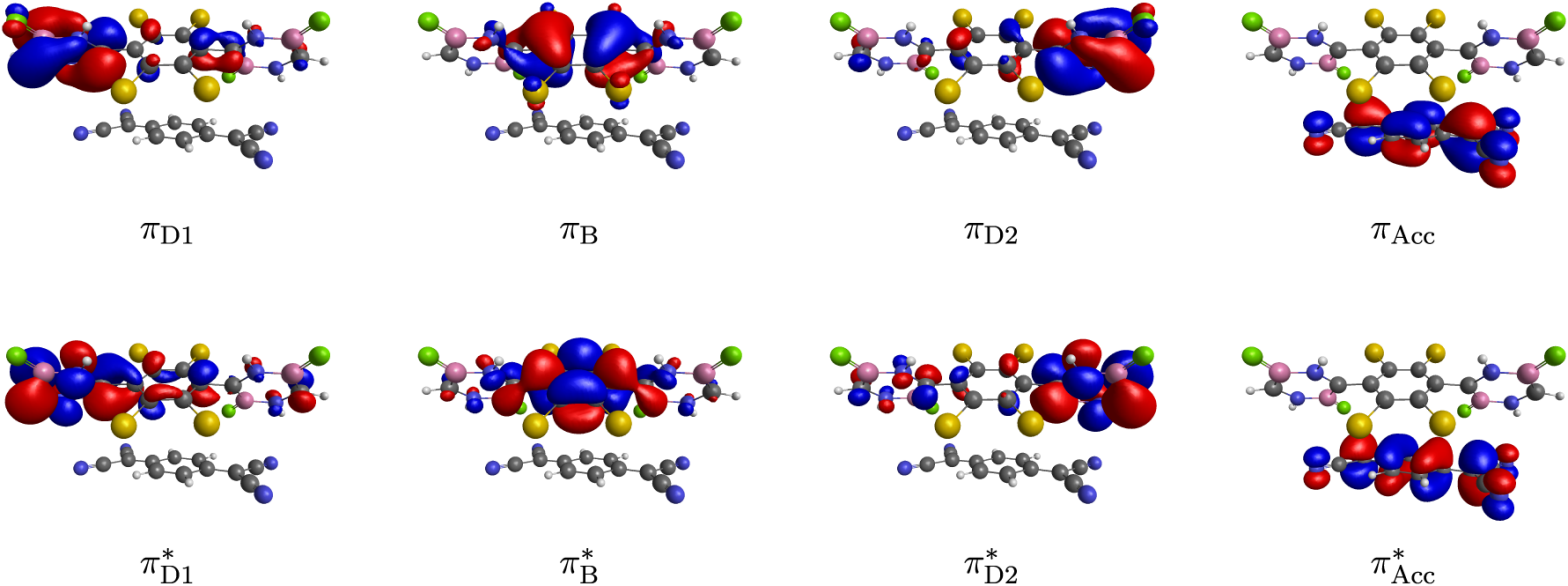
Diabatic molecular orbitals of the active space (with contour value 0.03). The H, B, C, N, F, and Cl atoms are described by the white, pink, gray, blue, green, and yellow spheres, respectively.

Figure 3 presents the characterization of the 19 diabatic states in terms of their dominant configurations and DMOs. The letters of each state represent the fragments of the DADB–TCNQ dimer, where the DMOs are localized. In detail, the first, second, and third letters denote the character of D1, B, and D2 of the donor molecule. The final paragraph describes the acceptor. The letters G, E, T, C, and A represent the ground-state character, locally excited character, and multiexcitonic character, as well as the character of radical cation and radical anion of a charge transfer state, respectively. C* denotes excited-state cationic character. The states are thus categorized into LE, CT, or ME states in the donor molecule (D), and CT or ME states between the donor and acceptor molecule (DA). Based on their character and for ease of discussion below, they are grouped into five subsets: LE, CT_D_, ME_D_, CT_DA_, and ME_DA_. The subset LE includes the locally excited states in the left and right diazadiborine rings of the donor and the acceptor molecule. The subset CT_D_ includes charge transfer states between the tetrachlorophenylene bridge and the diazadiborine rings of the donor molecule. The subset ME_D_ includes the multiexcitonic state formed by two coupled triplets located in the diazadiborine rings of the donor (TGTG) and state TETG, which contains two coupled triplets and an excitation in the tetrachlorophenylene bridge. The subsets CT_DA_ and ME_DA_ include all states with the acceptor in a radical anionic state and a triplet state, respectively. It is noted that, according to the chosen diabatization scheme, the diabatic ground state GGGG also includes contributions of configurations with doubly excited character (see SI). This is in line with the character of the adiabatic ground state.

**Figure 3:**
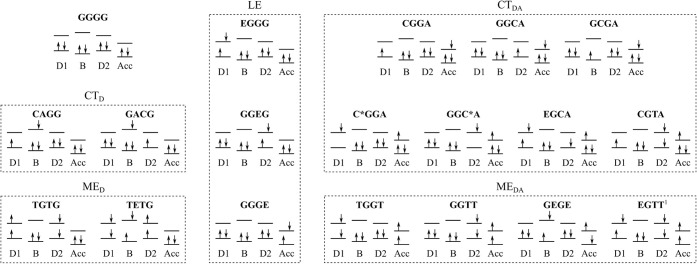
Configurations with the largest weights of the 19 diabatic states included in the quantum dynamical simulations. The occupation of the different DMOs are described by spin-up and spin-down arrows. The bottom and top bars denote the π and π* orbitals of each part of the molecule, respectively. A description of the different states can be found in the main text. ^1^The singlet excitation is located in fragments D1, D2, or Acc (see SI).

The diabatic electronic Hamiltonian ${Ĥ}_{\mathrm{el}}$ , presented in Figure 4, shows the relative diabatic state energies and interstate electronic couplings calculated at the XMCQDPT diabatic ground state minimum (see SI for details). The relative energies of the 18 diabatic singlet excited electronic states considered span a range from 1.89 to 4.91 eV, in line with the adiabatic vertical excitation energies (see SI). The LE states EGGG and GGEG, which are the initial electronic states of the quantum dynamical simulations discussed below, are found at 3.15 and 4.10 eV, respectively, and are localized in the diazadiborine rings of the donor. These states are strongly coupled to the CT_D_ states at 3.91 and 3.08 eV, characterized by the transfer of charge from the diazadiborine rings to the bridge (see Figure 3). The CT_D_ states themselves show a large coupling to the ME_D_ states at 3.16 eV (TGTG) and 4.91 eV (TETG). This, together with the fact that the coupling between the multiexcitonic state TGTG and the EGGG and GGEG locally excited states is smaller, suggests that the main channel responsible for the intramolecular SF process is a mediated mechanism.^3,12^

**Figure 4:**
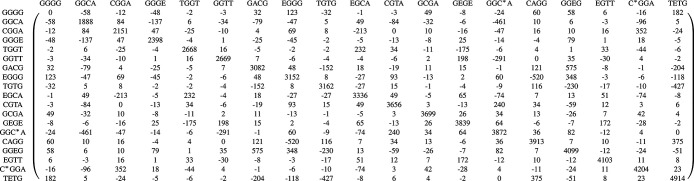
Diabatic electronic Hamiltonian ${Ĥ}_{\mathrm{el}}$ (in meV) of the donor–acceptor complex.

From the diabatic state energies, it is already possible to distinguish between favorable and unfavorable channels for efficient charge and energy transfer after the initial excitation. To minimize energy losses, a charge or energy transfer to high-lying states is desired, while a transfer to low-lying states is undesired. The population of ME_DA_ states would result from a high-lying energy transfer, while that of the states GCGA, C*GGA, GGC*A, EGCA, and CGTA would be the result of a high-lying charge transfer. In contrast, a transfer to the low-lying charge transfer states CGGA and GGCA, as well as the excited state in the acceptor GGGE, would be rather undesirable due to their comparatively low energies.

Regarding the donor–acceptor charge and energy transfer processes, our results indicate a complex mechanism characterized by an interplay between various diabatic states, linking these processes to SF. In the following section, we report the results obtained using quantum dynamical methods to gain insights into the underlying mechanisms driving the transfer processes.

### 3.2 Dynamics

To unravel the mechanisms underlying the SF, charge transfer, and energy transfer processes, and to address the role of molecular vibrations, we employ the vibronic model Hamiltonian introduced above (see eq 2). Using this framework, we investigate the dynamics of these processes, assuming an initial instantaneous population of the donor LE states EGGG and GGEG. Figure 5 (a) and (b) display the time evolution of the populations of the diabatic state subsets in the DADB–TCNQ dimer for the first 300 fs after the initial excitation of the EGGG and GGEG states, respectively. The insets present the dynamics for the first 10 fs.

**Figure 5:**
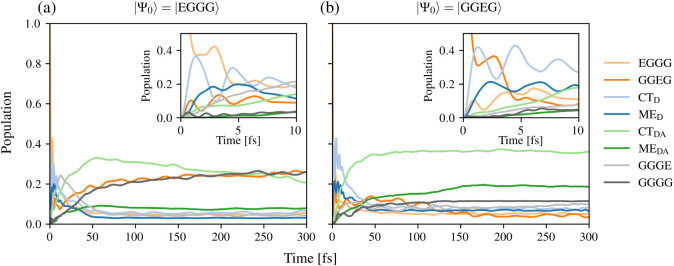
Time evolution of the population of the electronic states in the donor–acceptor complex using the vibronic model Hamiltonian Ĥ with the initial state |Ψ_0_⟩ set to (a) |EGGG⟩ and (b) |GGEG⟩.

In the case of the initial population of the EGGG state, the short-time dynamics show a rather complex interplay between various states. In detail, the population of the initially prepared LE state decreases rapidly to ∼40% in the first 2 fs. At the same time, the populations of the CT_D_ and ME_D_ states rise and reach ∼40% and ∼20%, respectively. Additionally, the populations of the GGEG, GGGE, and CT_DA_ states increase to a minor extent (see the inset of Figure 5 (a)). Subsequently, the population of the EGGG state and those of the CT_D_ and ME_D_ groups decrease to small and similar values, showing a plateau after ∼50 fs. This trend can also be observed for the LE state in the acceptor molecule (GGGE), whose population increases up to a value of ∼25% in the first 20 fs, to subsequently decreasing to similar values as those found for the EGGG state and the CT_D_ and ME_D_ groups, yet at slightly longer times. The CT_DA_ and ME_DA_ groups, as well as the GGEG and GGGG states, show an increase in population during the initial 50 fs, reaching values of ∼35% (CT_DA_), ∼10% (ME_DA_), and ∼20% (GGEG and GGGG). At longer times, however, the CT_DA_ group shows a slow but steady decrease, reaching a value of ∼25% at 300 fs, while the ME_DA_ group and the GGEG and GGGG states no longer show significant changes.

The dynamics when GGEG is the initially populated state (see Figure 5 (b)) exhibit both similarities and differences compared to those observed for the initial state EGGG. In detail, the short-time dynamics closely resemble those observed in the EGGG case. The population of GGEG rapidly decreases to a value of ∼30% at 2 fs. This decay occurs alongside a simultaneous increase in the total population of CT_D_ and ME_D_ groups. The populations of the GGEG, GGGE, and CT_DA_ states exhibit slight increases. Concerning the long-time dynamics, more significant differences can be observed with respect to the dynamics of the initial state EGGG. The population of the different states shows plateaus after 150 fs, with the largest values found for the CT_DA_ group (∼35%). In addition, the population of the GGGG state differs significantly from that found in the EGGG case, reaching a maximum of ∼10% over the full 300 fs simulation time, while EGGG does not show a sizable population. Finally, the ME_DA_ group exhibits a moderate increase in population, reaching values of ∼20%, significantly larger than those found for the EGGG initial state case. Both the CT_DA_ and ME_DA_ groups are the most populated sets of states at the end of the simulation. Thus, the charge and energy transfer processes are dependent on the initial electronic state and are more efficient for the higher-lying LE state GGEG. This can be explained on the basis of the energy of the state GGEG at 4.10 eV, which is higher than TGTG and closer to the range of the high-lying CT_DA_ (3.34 to 4.20 eV) and ME_DA_ (2.67 to 4.10 eV) states, which makes the generation of intramolecular ME_D_ states as well as charge and energy transfer more favorable.

For both initial states, the vibronic dynamics in Figure 5 show ultrafast, mediated intramolecular SF in the DADB dimer. After the initial excitation, the population of the CT_D_ states and the ME_D_ states increases subsequently, which is in agreement with the results reported for the isolated DADB molecule in the work of Zeng.^70^ In addition, the dynamics reveal intermolecular charge and energy transfer processes between the donor and acceptor moieties (population of the CT_DA_ and ME_DA_ groups). From the vibronic dynamics in Figure 5, however, the exact mechanisms of these processes are difficult to describe. To unveil the specific roles of the diabatic electronic states involved in these processes, and those of the vibrations, we discuss in the following results obtained using several reduced vibronic model Hamiltonians that incorporate different subsets of diabatic electronic states and vibrational modes.

### 3.3 Role of the Diabatic Electronic States

To analyze the role of the relevant diabatic states in the dynamics and unravel the charge and energy transfer mechanisms, we use vibronic model Hamiltonians with different subsets of diabatic states. We consider the full Hamiltonian Ĥ as described in eq 2 and the Hamiltonian ${Ĥ}_{\mathrm{inter}}$ , which is obtained from the full Hamiltonian by excluding all CT_D_ and ME_D_ states and their couplings. Additionally, we consider the Hamiltonian ${Ĥ}_{\mathrm{intra}}$ , which is the full Hamiltonian excluding the high-lying CT_DA_ states C*GGA, GGC*A, and GCGA and their couplings. In this manner, ${Ĥ}_{\mathrm{inter}}$ excludes the states involved in the population of the intramolecular ME_D_ states (i.e., the intramolecular SF process in the donor) and allows us to distinguish between a direct intermolecular transfer mechanism and a transfer mechanism mediated by the ME_D_ states. On the other hand, ${Ĥ}_{\mathrm{intra}}$ was chosen to investigate the transfer mechanism mediated by the ME_D_ states, since the high-lying CT_DA_ states play a significant role in the direct intermolecular charge and energy transfer processes.

Figure 6 shows the time evolution of the population of selected electronic states obtained using the three vibronic model Hamiltonians Ĥ , ${Ĥ}_{\mathrm{inter}}$ , and ${Ĥ}_{\mathrm{intra}}$ . In all three models, the initial state is set to GGEG. A first examination of Figure 6 reveals that the population of the intermolecular ME_DA_ states increases over ∼15% for all three models, indicating that a charge and energy transfer process from the donor to the acceptor molecule occurs in all cases. However, the nature of the different diabatic states underlying the transfer mechanism varies.

**Figure 6:**
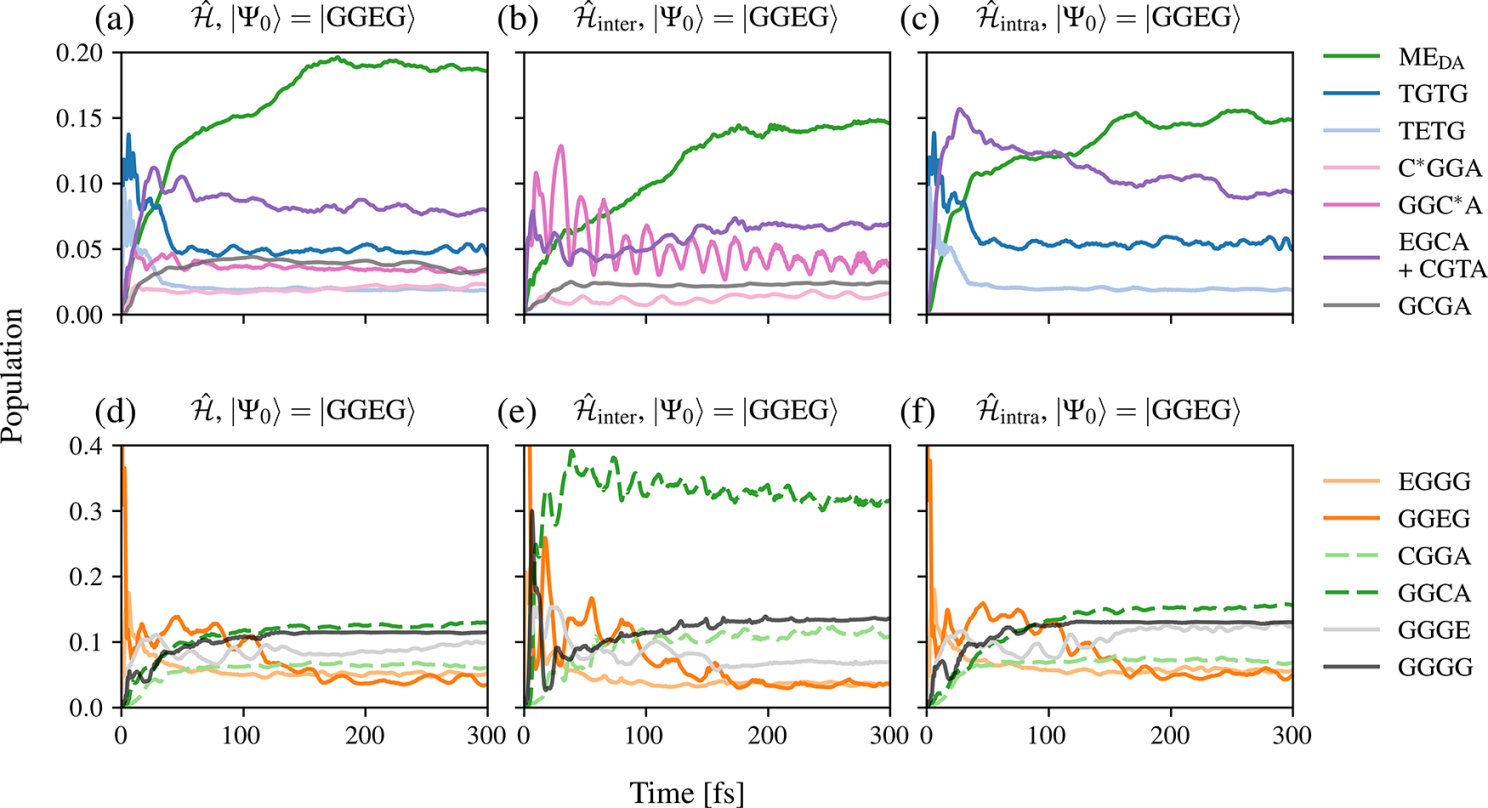
Time evolution of the population of selected electronic states with initial state |Ψ_0_⟩ = |GGEG⟩ of the full model Ĥ , model ${Ĥ}_{\mathrm{inter}}$ , and the model ${Ĥ}_{\mathrm{intra}}$ . The top graphs (a)–(c) present the populations of high-lying states, while the bottom graphs (d)–(f) display the populations of the locally excited and low-lying states.

The simulation, based on the full model Ĥ in Figure 6 (a) shows an increase of the TGTG and TETG states after the initial excitation in the first 15 fs, resulting from the intramolecular SF in the DADB molecule. Afterward, the population of the states GGC*A, C*GGA, GCGA, EGCA, and CGTA successively increases, like that of the ME_DA_ group.

This indicates an energy transfer mechanism from the ME_D_ to the ME_DA_ group (i.e., TGTG → GGTT) with the interplay of several high-lying intermolecular CT_DA_ states. In this mechanism, charges are transferred from the ME_D_ to the CT_DA_ states and further from the CT_DA_ states to the ME_DA_ states. The population analysis of the low-energy CT_DA_ states (CGGA and GGCA), EGGG, GGGE, and GGGG states is presented in Figure 6 (d) for the first 300 fs. The population of the CGGA, GGCA, GGGE, and GGGG states increases over time, with the total population of the states exhibiting CT character (CGGA and GGCA) reaching values larger than 15%. This increase in population results from strong coupling between the LE states in the donor molecule and the low-lying states (CGGA, GGCA, GGGE, and GGGG). This becomes especially visible in the population of the GGGE state, which oscillates in antiphase with GGEG. Altogether, these results show the existence of charge and energy transfer mechanisms from the initial state GGEG to low-lying states.

Removal of the CT_D_ and ME_D_ states from the diabatic basis ( ${Ĥ}_{\mathrm{inter}}$ model Hamiltonian, see Figure 6 (b)) leads to a scenario where an immediate rise in the population of the GGC*A state takes place upon relaxation of the initially populated GGEG state. The time evolution of the GCGA state is qualitatively similar to that found using the full vibronic model Ĥ , but shows a slightly lower population. Also, the population dynamics of the ME_DA_ group show a similar behavior to that obtained by the full model Ĥ , yet exhibit slightly lower (by ∼5%) values at the end of the simulation time. These results indicate an intermolecular SF process from the state GGEG to the ME_DA_ group (i.e., GGEG → GGTT) with mediating states GGC*A and GCGA. Further, we can infer that the CT_D_ and ME_D_ states, responsible for intramolecular SF, exhibit a minor role in this process, and both the intermolecular and intramolecular SF channels seem to be mostly decoupled. Figure 6 (e) reveals that, after the initial excitation, the population of the GGGG state rises almost instantly up to ∼30%, showing strong oscillations in antiphase to the GGEG state until roughly 40 fs, which indicates a strong coupling between GGEG and the ground state GGGG. In addition, the LE state EGGG and the LE state of the acceptor, GGGE, increase in population but slowly decrease with time. Differences from the full model Ĥ can be observed for the states CGGA and GGCA, whose population increases up to ∼10% and ∼40%, respectively. Further, the population of the GGCA state shows oscillations in antiphase to the high-lying GGC*A state, indicating a large coupling between the two.

Excluding the high-lying CT_DA_ states (C*GGA, GGC*A, and GCGA) in Figure 6 (c) leads to dynamics in line with the results found for the full vibronic model Hamiltonian Ĥ . After the initial relaxation of the GGEG state, an increase in the population of the TGTG and TETG states can be observed. The most significant difference between the two models is the larger population of EGCA and CGTA, which act as the mediating states after the ME_D_ generation to populate the intermolecular ME_DA_ states. This indicates a mediated charge and energy transfer mechanism following intramolecular SF (noticeable by the population of the ME_DA_ group). The population of the low-lying states (see Figure 6 (f)) shows a similar behavior to the full model Ĥ and is not further discussed. Thus, removing the high-lying CT_DA_ states (C*GGA, GGC*A, and GCGA) in ${Ĥ}_{\mathrm{intra}}$ mostly influences the charge transfer states (EGCA and CGTA) and multiexcitonic states.

### 3.4 Role of the Vibrational Modes

To analyze the role of the vibrational modes in the dynamics, we performed quantum dynamical simulations using the diabatic electronic Hamiltonian ${Ĥ}_{\mathrm{el}}$ (see Figure 4) and several vibronic model Hamiltonians that incorporate different subsets of vibrational modes selected based on their frequencies.

Figure 7 (a) and (b) show the electronic-only dynamics of the population of the different diabatic states after the initial excitation of the states EGGG and GGEG, respectively. For both initial states, the population of the CT_D_ group increases immediately after the excitation. This is followed by an increase in the population of the other LE state in the donor (i.e., the GGEG state increases after excitation of the EGGG state and vice versa) and the ME_D_ group, while the population of the intermolecular groups CT_DA_ and ME_DA_ does not significantly increase. Comparing the dynamics to the full vibronic dynamics depicted in Figure 5 reveals that the intramolecular SF process also occurs purely electronically. The pronounced difference to the full vibronic dynamics is in the population of the intermolecular CT and ME states. This points out that molecular vibrations are instrumental in enabling the intermolecular charge and energy transfer processes in the donor–acceptor complex.

**Figure 7:**
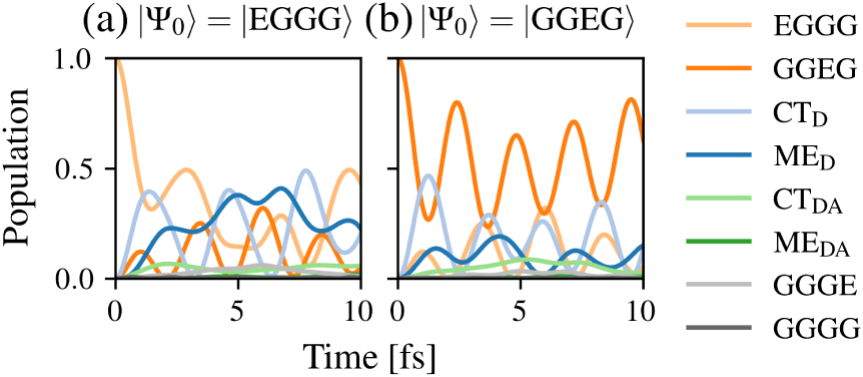
Time evolution of the state populations in the DADB–TCNQ dimer based on the diabatic electronic Hamiltonian ${Ĥ}_{\mathrm{el}}$ with the initial state |Ψ_0_⟩ set to (a) |EGGG⟩ and (b) |GGEG⟩.

To get further insights into the specific roles of the vibrational modes in these processes, we carried out dynamical simulations using three vibronic model Hamiltonians. Specifically, these models were constructed by incorporating different sets of vibrational modes, namely vibrational modes with frequencies ω_*m*_ between 100 cm^–1^ and 1000 cm^–1^ (model ${Ĥ}_{1}$ ), vibrational modes with ω_*m*_ > 1000 cm^–1^ (model ${Ĥ}_{2}$ ) and only ring-breathing vibrational modes of the tetrachlorophenylene donor bridge with ω_*m*_ between 1000 cm^–1^ and 1200 cm^–1^ (model ${Ĥ}_{3}$ ).

In the following, we focus on the intermolecular charge and energy transfer processes since the population of the ME_D_ group and ultrafast intramolecular SF in the DADB molecule also occur in the electronic dynamics. The initial population of the ME_D_ is independent of the vibrational modes, which only influence the details of the dynamics afterward (see SI). The dynamics of model ${Ĥ}_{1}$ incorporating normal modes with 100 cm^–1^ < ω_*m*_ < 1000 cm^–1^ (see Figure 8 (a) and (d)) show a different behavior compared to the full model Ĥ . After the initial excitation, the ME_D_ group, including the states TGTG and TETG, rises immediately in population to ∼10%. The TGTG state further increases to ∼17% at about 30 fs and then decreases until it reaches a plateau, similar to the state TETG. A slower increase in population can be observed for the states EGCA and CGTA, the GCGA state, and the ME_DA_ group, reaching ∼25%, ∼22%, and ∼15% after 300 fs, respectively. The three curves show oscillating behavior after about 100 fs. In detail, local maxima of the ME_DA_ group are observable at local minima of the GCGA state, and vice versa, which indicates a strong coupling between the two. The population of the GGC*A state reaches higher values compared to the full model Ĥ and increases up to a plateau at ∼8%, similar to the value reached by the state TGTG. At 250 fs, its population shows a maximum value (together with the ME_DA_ group), while similarly, the states EGCA and CGTA, as well as the GCGA state, exhibit local minima, which also indicates a coupling between these states. The population of the C*GGA state stays low throughout the simulation. Regarding the low-lying states in Figure 8 (d), their populations remain at low values below ∼5%. Differences from the full model Ĥ can especially be observed for the states GGCA and GGGG, which barely rise in the ${Ĥ}_{1}$ model, while they exhibit populations up to ∼10% in Ĥ . Thus, the vibrational modes with 100 cm^–1^ < ω_*m*_ < 1000 cm^–1^ do not induce the charge and energy transfer to the low-lying states. Further, the charge and energy transfer to the ME_DA_ group is less efficient and slower compared to the full model Ĥ . The high populations of the states GCGA, EGCA, and CGTA indicate a reduction of the mediated transfer process to the ME_DA_ group. Nevertheless, charge and energy transfer from the ME_D_ to the ME_DA_ group remains possible, even if less efficient, since the plateau of TGTG stays at larger values. The points made above could be explained by the nature of some of the vibrational modes incorporated. They change the intermolecular distance or relative orientation between the molecules and thus influence the effective coupling, which influences the charge and energy transfer between the two molecules.^96^

**Figure 8:**
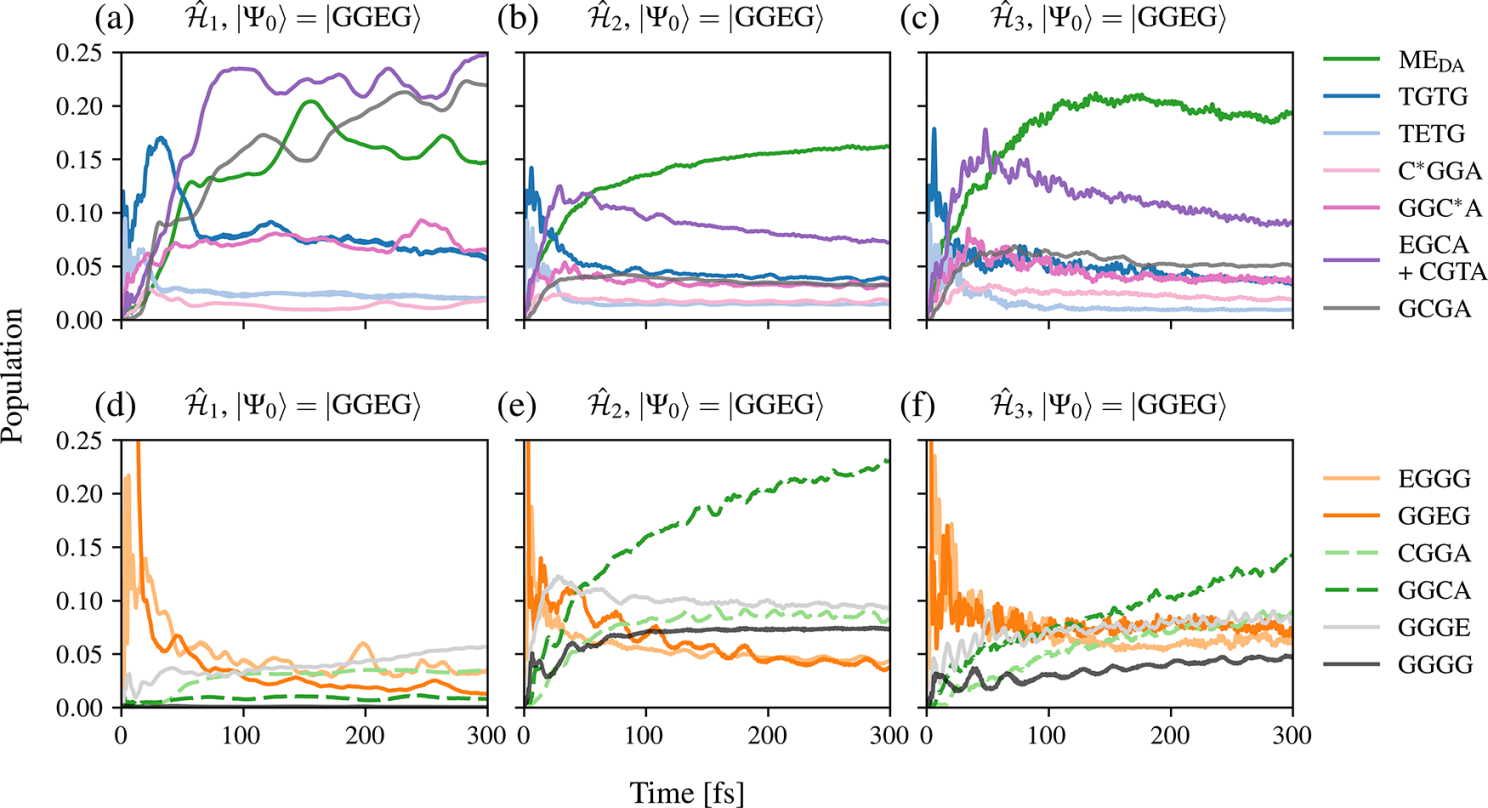
Time evolution of the population of selected electronic states with initial state |Ψ_0_⟩ = |GGEG⟩ for the first 300 fs of model ${Ĥ}_{1}$ , the model ${Ĥ}_{2}$ , and model ${Ĥ}_{3}$ . The top graphs (a)–(c) present the populations of high-lying states, while the bottom graphs (d)–(f) display the populations of the locally excited and low-lying states.

In the model including high-frequency vibrational modes with ω_*m*_ > 1000 cm^–1^ (see Figure 8 (b) and (e)), the population of the high-lying states shown in Figure 8 (b) exhibits similar dynamics to the full model Ĥ calculation but overall reaches lower values. Some differences can also be noted in the dynamics of the low-lying states, where the population of the state GGCA continuously rises up to ∼23% after 300 fs. The similarity in the dynamics of the high-lying states indicates that the vibrational modes with ω_*m*_ > 1000 cm^–1^ are contributors to both the SF (intra- and intermolecular) and the intermolecular charge and energy transfer processes. Additionally, the charge transfer process to the low-lying GGCA state is enhanced. It is important to mention the nature of the high-frequency vibrational modes, ω_*m*_ > 1200 cm^–1^, which are partially located in the acceptor molecule.

The dynamics obtained from removing these modes and only including the ring-breathing modes in the tetrachlorophenylene bridge of the donor molecule with 1000 cm^–1^ < ω_*m*_ < 1200 cm^–1^ are presented in Figure 8 (c) and (f). Here, the population of the high-lying states in Figure 8 (c) is similar to the full model Ĥ , but shows a less smooth behavior, which can be explained on the basis of the small number of five vibrational modes included in the simulation. Comparing the two simulations closely sheds some light on slight differences. The population of the states TGTG, EGCA, and CGTA, and the ME_DA_ group reach values of ∼17% for the first two (TGTG, EGCA, and CGTA) and ∼20% for the latter. In particular, the high population shown by the TGTG state suggests that efficient intramolecular SF is driven by the ring-breathing modes of the tetrachlorophenylene bridge moiety of DADB. A comparison of the time evolution of the population of the low-lying states in Figure 8 (f) to the full model Ĥ shows steadily increasing populations of the states CGGA, GGCA, GGGE, and GGGG over time. On the basis of these results, we conclude that the ring-breathing modes of the donor bridge essentially drive the SF (intra- and intermolecular) as well as the charge and energy transfer in the complex.

To summarize, the dynamics obtained with the full model Hamiltonian Ĥ are the result of the complex interplay of the different vibrational modes of the system. The ring-breathing modes of the donor bridge drive the SF, as well as charge and energy transfer to the ME_DA_ group, while the vibrational modes with ω_*m*_ > 1200 cm^–1^ induce a transfer to the low-lying states, which can be quenched, including the vibrational modes with 100 cm^–1^ < ω_*m*_ < 1000 cm^–1^.

### 3.5 Charge and Energy Transfer Mechanisms

Based on the results presented above, in this section, we provide a mechanistic analysis of the charge and energy transfer processes in the donor–acceptor complex. For simplicity, Figure 9 presents the transfer mechanisms split into three possible pathways: (a) intermolecular SF, (b) charge and energy transfer after intramolecular SF, and (c) charge and energy transfer to low-lying states. The two pathways: (a) and (b), are high-energy transfer mechanisms since high-lying states are involved, while pathway (c) involves the low-lying states.

**Figure 9:**
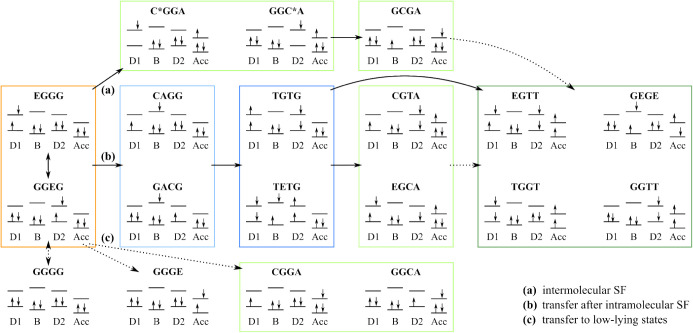
Scheme of the charge and energy transfer mechanisms in the donor–acceptor complex based on the vibronic dynamics. Each state is represented by its configuration state function with the highest coefficient. The arrows indicate the transition from one state to another, where the dotted arrows indicate a transition driven by vibrational modes with ω*_m_* > 1000 cm^–1^. In accordance to Figure 5 the LE states in the donor are marked in orange, the CT_D_ group in light blue, the ME_D_ group in dark blue, the CT_DA_ group in light green, and the ME_DA_ group in dark green.

The possible mechanism of intermolecular SF is described by pathway (a). After initial excitation, the electron in the π_D1_/π_D2_ orbital transfers to the $\pi_{\mathrm{Acc}}^{*}$ orbital (C*GGA/GGC*A), from where the CT state GCGA is populated, which incorporates the bridge in a cationic state. The high-frequency modes with ω_*m*_ > 1000 cm^–1^ drive the transition from the state GCGA to the ME_DA_ group, where not only GEGE but also the other ME_DA_ states are populated. Furthermore, these vibrational modes enable a large coupling between the LE states in the donor and the GGCA state. The transfer following intramolecular SF in the donor molecule is described by pathway (b). After the initial excitation, a strong coupling to the CT_D_ states (CAGG and GACG) transfers the electron from $\pi_{D1}^{*}/\pi_{D2}^{*}$ to $\pi_{B}^{*}$ . From this intermediate state, the ME_D_ states (TGTG and TETG) are populated, describing the intramolecular SF in the donor molecule. Afterward, an electron from the $\pi_{D1}^{*}/\pi_{D2}^{*}$ is transferred to the acceptor molecule (population of the CGTA and EGCA states), followed by an electron in π_Acc_ being transferred back to $\pi_{D1}^{*}/\pi_{D2}^{*}$ . Nevertheless, triplet energy transfer to the acceptor molecule can occur directly without the intermediate states CGTA and EGCA. Both pathways describe triplet exciton transfer from ME_D_ to the ME_DA_. The transfer to low-lying states is described by pathway (c). This mechanism is driven, similar to the intermolecular SF, by the vibrational modes with ω_*m*_ > 1000 cm^–1^ and is increased by vibrational modes partially located in the acceptor molecule with even larger frequencies of ω_*m*_ > 1200 cm^–1^.

Overall, the donor–acceptor system exhibits three competing charge and energy transfer mechanisms from the LE states of the donor molecule to those of the acceptor molecule. These transfer mechanisms depend not only on the electronic states but also on the vibrational modes. To increase the efficiency of the transfer processes in the donor–acceptor complex, the population of the ME_DA_ group should be increased, while that of the low-lying states (GGGE, CGGA, and GGCA) should be decreased.

To reduce the transfer to low-lying states, a different acceptor molecule could be chosen with higher-lying CT states CGGA and GGCA. In addition, one could control the intermolecular distance between the molecules. To increase the transfer efficiency, the addition of a second acceptor molecule in an acceptor–donor–acceptor configuration could provide the ability to extract two excitons or electrons instead of one. Thus, the triplets of the ME_DA_ states generated would be more delocalized, and hence the efficiency of charge or energy transfer could be increased. Furthermore, replacement of the chlorine atoms of the tetrachlorophenylene bridge with other groups influences the ring-breathing vibrational modes of the donor bridge, their coupling strengths, and electronic couplings,^70^ which might result in a more efficient transfer mechanism.

## 4 Conclusions

We have investigated charge and energy transfer processes in a donor–acceptor complex consisting of a singlet fission-active donor (bis­(diazadiborine)-based chromophore) and an acceptor (tetracyanoquinodimethane) molecule using high-level *ab initio* multireference electronic structure calculations and quantum dynamical simulations.

The results show a complex interplay of singlet fission, charge transfer, and energy transfer mechanisms. Our analysis, using different model Hamiltonians varying in the diabatic electronic states and vibrational modes, revealed that the relaxation of the initially populated locally excited states in the donor molecule proceeds through three competing charge and energy transfer mechanisms namely, intermolecular singlet fission from the donor to the acceptor molecule, charge and energy transfer to the acceptor molecule following intramolecular singlet fission in the donor molecule, and charge and energy transfer to low-lying states. The intermolecular singlet fission process to the intermolecular multiexcitonic states is mediated by charge transfer between the donor and acceptor. Similarly, the transfer from intramolecular multiexcitonic states (generated via ultrafast singlet fission in the donor) to intermolecular multiexcitonic states also proceeds through donor–acceptor charge transfer states. In addition, charge and energy transfer occur to low-lying intermolecular charge transfer states and the locally excited state of the acceptor molecule.

The results also show that singlet fission, charge transfer, and energy transfer processes are strongly influenced by electronic-vibrational coupling. Specifically, all three charge and energy transfer pathways are primarily driven by the ring-breathing vibrational modes in the donor bridge, with frequencies between 1000 cm^–1^ and 1200 cm^–1^. Vibrational modes above 1200 cm^–1^, partially localized in the acceptor, enhance the transfer to low-lying states. In contrast, vibrational modes below 1000 cm^–1^, which change the intermolecular distance, quench this transfer.

Our analysis gives insights into future design possibilities to reduce the transfer to low-lying states and increase the efficiency of singlet fission-influenced charge and energy transfer from the donor to the acceptor molecule. This could improve the performance of singlet fission-based devices.

## Supplementary Material



## Notes

Further details of the adiabatic electronic structure calculations, diabatization, Cartesian coordinates, vibronic model Hamiltonian, and quantum dynamical simulations (PDF)
