# Supplementary material for: Unraveling Charge and Energy Transfer in a Singlet Fission Donor–Acceptor Complex: An Ab Initio Quantum Dynamical Study
Source: J Chem Theory Comput. 2026 Feb 25;22(5):2129–40. doi: 10.1021/acs.jctc.5c01945 (PMC12980725; doi:10.1021/acs.jctc.5c01945)
Supplement: Supplementary file 1 [file ct5c01945_si_001.pdf]

Supporting Information for Unraveling Charge and Energy Transfer in a Singlet Fission Donor-Acceptor Complex: An Ab Initio Quantum Dynamical Study

Karin S. Thalmann,*,† Pedro B. Coto,‡ and Michael Thoss†

†*Institute of Physics, University of Freiburg, Hermann-Herder-Str. 3, 79104 Freiburg, Germany.*

‡*Nanomaterials and Nanotechnology Research Center (CINN), CSIC-University of Oviedo-Principality of Asturias and Donostia International Physics Center (DIPC), Avda. de la Vega 4-6, 33940 El Entrego, Spain.*

E-mail: karin.thalmann@physik.uni-freiburg.de

Contents

S1 Adiabatic Electronic Structure Calculations **S3**

S2 Diabatization **S5**

S3 Cartesian Coordinates **S5**

S4 Vibronic Model Hamiltonian **S9**

S4.1 Fit of the Potential Energy Surfaces S9

S4.2 Transformation to the Ground State at XMCQDPT Level of Theory S10

S4.3 Vibrational Modes and Coupling Constants S12

S5 Quantum Dynamical Simulations **S58**

S5.1 Computational Details S58

S5.2 Dynamics of the Intramolecular Singlet Fission in the DADB Molecule . . . S59

References **S64**

S1 Adiabatic Electronic Structure Calculations

This section provides further computational details on the electronic structure calculations performed for this study.

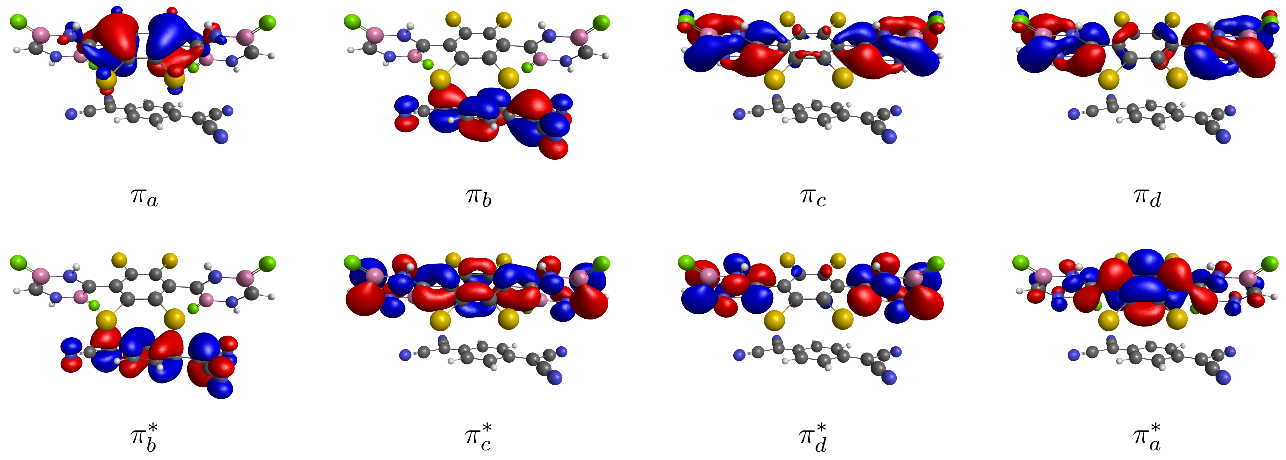

Figure S1: Optimized MCSCF molecular orbitals of the active space (with contour value 0.03). The H, B, C, N, F, and Cl atoms are described by the white, pink, gray, blue, green and yellow spheres, respectively.

As described in the main text, we performed complete active space self consistent field theory (CASSCF) calculations for 19 states with equal weights, an active space of 8 active orbitals and 8 active electrons, as well as the basis set cc-pVDZ.[1] The corresponding optimized MCSCF active orbitals are shown in Figure S1. The orbitals are localized on either the donor or acceptor molecule, and form four sets of bonding and antibonding π-like orbitals. The first set (π_a - π_a^*) is rather localized in the bridge of the donor molecule, the second set (π_b - π_b^*) is localized in the acceptor molecule, and the last two sets (π_c - π_c^* and π_d - π_d^*) are distributed over the donor molecule.

Additionally, we calculated the permanent dipole moment μ_{CAS} and the oscillator strength f_{CAS} with respect to the ground state S_0 for each state. To account for the dynamic correlation effects, we performed an extended multiconfigurational quasidegenerate perturbation theory (XMCQDPT) calculation[2] with an intruder avoidance shift of 0.02 $(\mathrm{au})^2$ and matched each state to their largest CASSCF state contributions (contrib.). The relative state energies

Table S1: Adiabatic relative energies (in eV) calculated at the (a) CASSCF/cc-pVDZ (ΔE_{CAS}) and (b) XMCQDPT/cc-pVDZ (ΔE_{XPT}) theoretical levels, dipole moments μ_{CAS} (in Debye) and oscillator strengths f_{CAS} calculated at CASSCF/cc-pVDZ. The largest contributions of the CASSCF states to the XMCQDPT states is presented by the contrib. column. The state character char. is given by GS for ground state, ME for multiexcitonic state, ES for excited state and CT for charge transfer state. The subindex denotes, whether the character is in the donor D, acceptor A, or in both the donor and acceptor DA.

state	ΔE_{CAS}	μ_{CAS}	f_{CAS}
S_0	0.00	0.560	0.0000
S_1	1.91	0.567	$< 10^{-5}$
S_2	2.67	0.288	0.0006
S_3	2.69	0.353	0.0002
S_4	3.04	6.826	0.0150
S_5	3.39	8.147	0.0072
S_6	3.64	0.387	0.0001
S_7	4.08	7.377	0.0005
S_8	4.33	8.957	0.0062
S_9	4.52	0.613	0.0006
S_{10}	4.68	0.566	0.0384
S_{11}	5.30	0.567	0.5314
S_{12}	5.53	1.307	2.0494
S_{13}	5.63	4.923	0.3564
S_{14}	5.73	4.622	0.0069
S_{15}	5.76	0.936	0.0074
S_{16}	5.81	4.390	0.0056
S_{17}	5.84	5.835	0.0065
S_{18}	6.10	0.440	0.0056

(a) CASSCF

state	ΔE_{XPT}	contrib.	char.
$S_{0,\text{XPT}}$	0.00	S_0	GS
$S_{1,\text{XPT}}$	1.87	S_4	CT_{DA}
$S_{2,\text{XPT}}$	2.16	S_5	CT_{DA}
$S_{3,\text{XPT}}$	2.42	S_{11}	ES_{A}
$S_{4,\text{XPT}}$	2.65	S_2	ME_{DA}
$S_{5,\text{XPT}}$	2.70	S_3	ME_{DA}
$S_{6,\text{XPT}}$	2.72	S_{12}/S_{18}	$\text{ES}_{\text{D}}/\text{CT}_{\text{D}}$
$S_{7,\text{XPT}}$	2.75	S_{12}/S_{18}	$\text{ES}_{\text{D}}/\text{CT}_{\text{D}}$
$S_{8,\text{XPT}}$	2.96	S_1	ME_{D}
$S_{9,\text{XPT}}$	3.49	S_7	CT_{DA}
$S_{10,\text{XPT}}$	3.67	S_8	CT_{DA}
$S_{11,\text{XPT}}$	3.85	S_{14}	CT_{DA}
$S_{12,\text{XPT}}$	3.90	S_9	$\text{ES}_{\text{D}}/\text{CT}_{\text{D}}$
$S_{13,\text{XPT}}$	4.05	S_{10}	$\text{CT}_{\text{D}}/\text{ME}_{\text{D}}$
$S_{14,\text{XPT}}$	4.10	S_6/S_{16}	ME_{DA}
$S_{15,\text{XPT}}$	4.20	S_{13}	CT_{DA}
$S_{16,\text{XPT}}$	4.30	S_{16}/S_{17}	CT_{DA}
$S_{17,\text{XPT}}$	4.35	S_6/S_{16}	ME_{DA}
$S_{18,\text{XPT}}$	5.15	S_{15}	ME_{D}

(b) XMCQDPT

ΔE_{CAS} of 19 lowest-lying singlet excited states at CASSCF level of theory, the permanent dipole moments μ_{CAS}, and the oscillator strengths f_{CAS} to the ground state S_0 are presented in Table S1 (a). The relative state energies ΔE_{XPT} calculated at XMCQDPT/cc-pVDZ level of theory, the contrib., and the character of the different states (char.) are shown in Table S1 (b). The character of the states are assigned according to f_{CAS}, μ_{CAS}, and the configuration state functions at XMCQDPT level of theory. However, the states of the donor-acceptor complex are heavily mixed, making a clear assignment of the state characters in the adiabatic basis difficult.

The large permanent dipole moments μ_{CAS} compared to the ground state indicate a

charge transfer (CT) state. In our system, CT states are intramolecular in the donor (D) or intermolecular delocalized over the donor and acceptor (DA). A large oscillator strength f_{CAS} to the ground state typically describes a bright (absorbing) excited state (ES). The ES state can be located in the donor (D) or in the acceptor (A) molecule. Multiexcitonic (ME) states usually show a low dipole transition moment and oscillator strength. They consist of two excitons, which can be located intra- (in D or A) or intermolecular (in DA). Table S1 shows that the states $S_{1,\mathrm{XPT}}$ and $S_{2,\mathrm{XPT}}$ are of CT character. The states $S_{6,\mathrm{XPT}}$ and $S_{7,\mathrm{XPT}}$ show a strong mixing between a CT and ES character. High-lying states are of CT or ME character.

The calculations above were carried out using GAMESS 2020 R2.[3]

S2 Diabatization

The diabatization was performed using the fourfold way introduced by Nakamura and Truhlar[4–6] as implemented in GAMESS 2020 R2.[3] Figure S2 shows the configuration state functions (CSFs), their electronic occupations, and weights included in the diabatic states. The letter code of each CSF describes the adiabatic character of the left (D1) and right (D2) diazadiborine ring, the bridge (B) moiety, and the acceptor (Acc) molecule. In detail, g, e, d, t, c, and a denote the ground state, singly excited state, doubly excited state, triplet state, cation and anion, respectively. The electronic diabatic Hamiltonian obtained after the diabatization is presented in Figure S3.

S3 Cartesian Coordinates

The optimized ground state equilibrium geometry of the donor-acceptor complex is provided in Table S2.

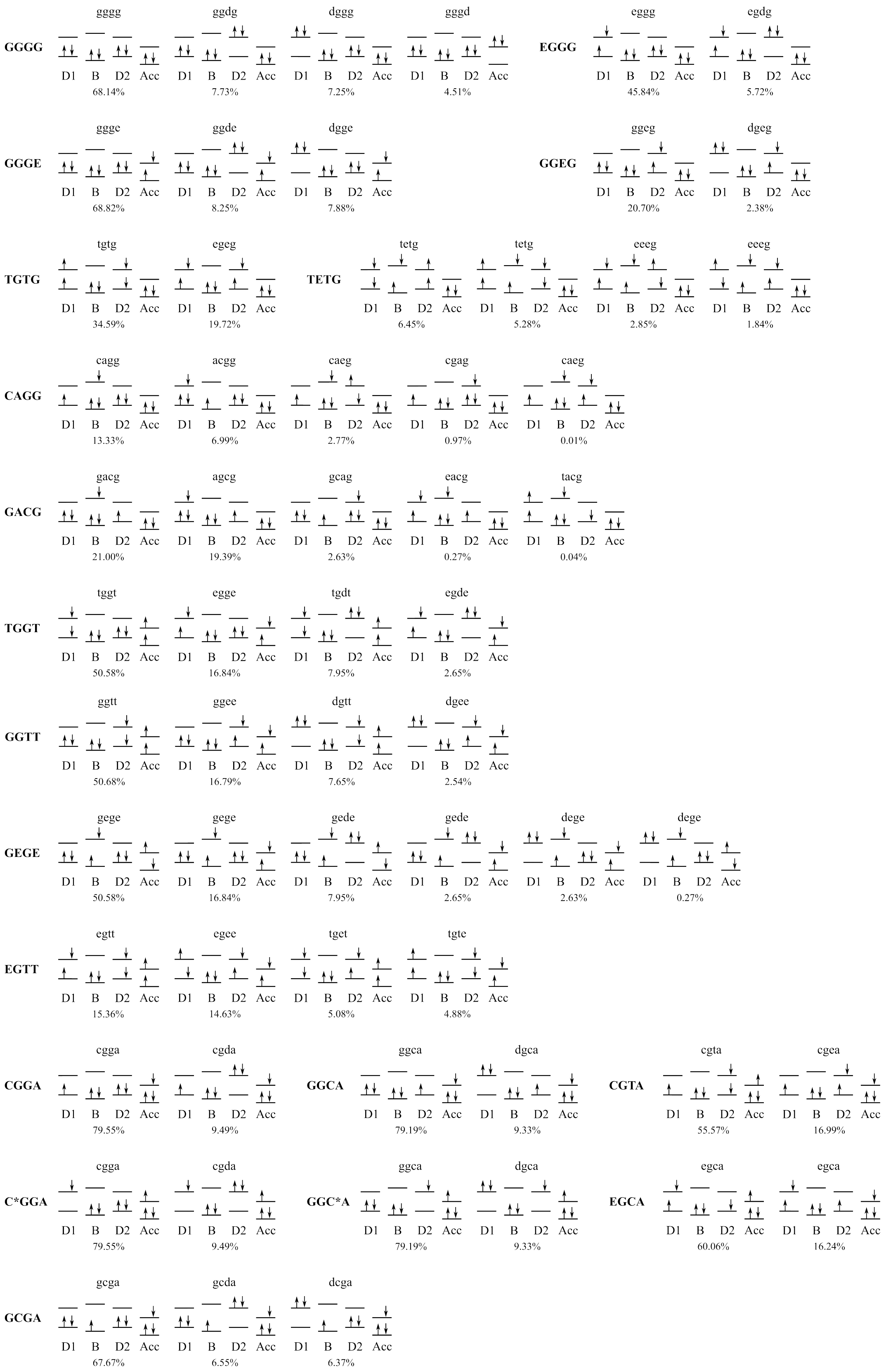

Figure S2: Configuration state functions, their electronic occupations, and weights for each diabatic state.

	GGGG	GGCA	CGGA	GGGE	TGGT	GGTT	EGGG	TGTG	GACG	GGEG	EGCA	CGTA	GCGA	CAGG	GGC*A	GEGE	EGTT	C*GGA	TETG
GGGG	0	4	-25	-94	-1	-4	-22	38	-232	-312	2	-4	31	305	-29	-5	2	-17	124
GGCA	4	1867	83	-87	11	-71	-26	0	-30	-18	20	0	23	0	-80	4	-5	-43	-6
CGGA	-25	83	2073	46	-42	-16	30	4	8	-4	-83	19	-19	2	0	-1	13	135	-9
GGGE	-94	-87	46	2365	-3	3	-33	5	-48	49	-6	-12	-1	5	-16	6	0	14	-11
TGGT	-1	11	-42	-3	2650	12	-3	-2	-3	0	155	22	-9	2	-3	-112	18	-60	-2
GGTT	-4	-71	-16	3	12	2674	-2	-2	4	12	-6	16	11	1	-183	122	-21	2	0
EGGG	-22	-26	30	-33	-3	-2	2780	30	-102	15	-9	43	-4	-386	17	5	0	8	-74
TGTG	38	0	4	5	-2	-2	30	3018	-125	-146	1	31	0	109	-9	0	-8	8	-307
GACG	-232	-30	8	-48	-3	4	-102	-125	3256	540	26	-13	6	162	9	8	-3	3	-297
GGEG	-312	-18	-4	49	0	12	15	-146	540	3268	5	-29	-7	-20	48	-2	-4	-12	23
EGCA	2	20	-83	-6	155	-6	-9	1	26	5	3441	20	-2	-5	-62	37	51	-88	6
CGTA	-4	0	19	-12	22	16	43	31	-13	-29	20	3745	-3	18	250	-12	39	0	-19
GCGA	31	23	-19	-1	-9	11	-4	0	6	-7	-2	-3	3798	12	7	38	2	14	1
CAGG	305	0	2	5	2	1	-386	109	162	-20	-5	18	12	3815	20	-2	4	1	365
GGC*A	-29	-80	0	-16	-3	-183	17	-9	9	48	-62	250	7	20	3989	31	-13	1	-11
GEGE	-5	4	-1	6	-112	122	5	0	8	-2	37	-12	38	-2	31	4120	120	-14	2
EGTT	2	-5	13	0	18	-21	0	-8	-3	-4	51	39	2	4	-13	120	4199	16	2
C*GGA	-17	-43	135	14	-60	2	8	8	3	-12	-88	0	14	1	1	-14	16	4230	14
TETG	124	-6	-9	-11	-2	0	-74	-307	-297	23	6	-19	1	365	-11	2	2	14	4853

Figure S3: Diabatic electronic Hamiltonian $\hat{\mathcal{H}}_{\text{el}}$ (in meV) of the donor-acceptor complex at the DFT ground state equilibrium structure.

Table S2: Cartesian coordinates of the equilibrium structure of the donor-acceptor complex (in Å).

atom	x	y	z
C	-1.4349068	-0.3026975	0.0664304
C	-0.7295769	-1.4871691	0.3291203
C	0.6751807	-1.5489282	0.1892274
C	1.3438230	-0.3646627	-0.1927779
C	0.6422028	0.8272332	-0.4218019
C	-0.7650323	0.8766328	-0.3250064
C	-1.5242152	2.0941381	-0.6884151
C	1.4051731	-2.8157207	0.4140417
B	-1.9254203	4.5199399	-0.2543232
C	-2.8268943	4.5790496	-1.4608179
N	-3.0272160	3.4420524	-2.1699640
B	-2.4524142	2.1574668	-1.8623823
N	-1.3254266	3.2456477	0.0253563
B	3.2181747	-3.9669681	1.6937533

C	3.0164695	-5.2021818	0.8561325
N	2.3749500	-2.8488772	1.3830249
B	1.2009284	-4.0582093	-0.3970443
F	-1.6363444	5.5651268	0.5661170
F	-2.7529266	1.1038193	-2.6772211
Cl	1.5059080	2.2647392	-0.9217134
Cl	-3.1647144	-0.2557117	0.2890714
Cl	-1.5882937	-2.8942767	0.8987497
Cl	3.0727396	-0.4159478	-0.4734825
F	0.2917089	-4.1979182	-1.4059767
F	4.1247333	-3.8348379	2.7009246
N	2.0592509	-5.1766484	-0.1022199
H	-3.3223280	5.4709846	-1.8350355
H	-3.6059809	3.5111988	-3.0071696
H	-0.6796504	3.1895196	0.8114388
H	3.5870334	-6.1231385	0.9413960
H	2.5101650	-1.9941919	1.9209074
H	1.9573978	-6.0111720	-0.6779407
N	-0.7843122	-5.0377873	-4.8237243
N	-2.6126183	3.1385171	-5.4130939
H	-1.4491483	-2.1475755	-4.9478594
H	-1.9805448	0.2310914	-5.1264912
C	-0.0316026	-4.2146887	-4.4878878
C	-0.7088883	-1.4031751	-4.6705804
C	-1.0028933	-0.0817036	-4.7757620
C	-1.5847522	2.7491786	-5.0263475

C	0.8896767	-3.1978965	-4.0959224
C	0.5792523	-1.8458917	-4.1819285
C	-0.0360177	0.9297607	-4.4071321
C	-0.3308732	2.2853735	-4.5260215
C	2.1487954	-3.6538997	-3.6043714
C	1.5382783	-0.8341939	-3.7926434
C	1.2476690	0.4871910	-3.9064108
C	0.6025422	3.3061532	-4.1740100
N	3.1713576	-4.0245985	-3.1867139
H	2.5029235	-1.1433672	-3.4019580
N	1.3639911	4.1412847	-3.8912079
H	1.9812766	1.2295165	-3.6105729

S4 Vibronic Model Hamiltonian

The Hamiltonian used for the quantum dynamical simulations includes the vibrational modes and vibronic couplings between the electronic states. In this section, we provide further details on the parametrization of the vibronic model Hamiltonian used in our quantum dynamical simulations

S4.1 Fit of the Potential Energy Surfaces

To obtain the potential energy surfaces the complex is distorted in dimensionless steps of 0.1 away from the ground state equilibrium structure along every vibrational mode. The vibrational modes with $\omega < 100$ cm^{-1} and $\omega > 100$ cm^{-1} were distorted up to 0.5 and 0.2 from the equilibrium structure, respectively. At the different points the diabatic energies and couplings were calculated using diabatization in the fourfold way. Further the linear coupling constants κ are extracted using a linear fitting function. An example of the surfaces and their

fits including the potential energy of the vibrational mode for the states GGGG, EGGG, GGEG, and TETG of the mode $\omega = 1045.06\ \text{cm}^{-1}$ are shown in Figure S4.

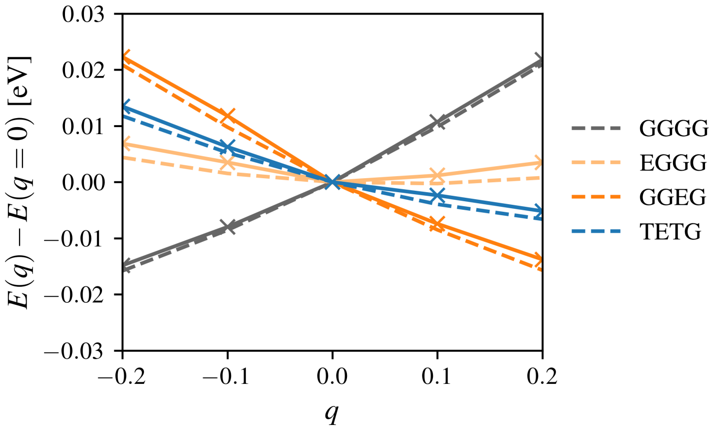

Figure S4: Potential energy surface of vibrational mode $\omega = 1045.06\ \text{cm}^{-1}$ for the states GGGG, EGGG, GGEG, and TETG referenced to their respective diabatic energies at $q = 0$. The crosses mark the values at different q after the diabatization in the fourfold way. The dashed lines present the fitted surfaces including the potential energy of the vibrational mode.

S4.2 Transformation to the Ground State at XMCQDPT Level of Theory

Since the ground state equilibrium structure is obtained at DFT level of theory, the Hamiltonian is transformed to the equilibrium geometry of the ground state at XMCQDPT level of theory to increase the accuracy of the model. To find the minimum of the multidimensional Hamiltonian of the ground state, the derivative of the Hamiltonian along the coordinates q is set to zero

$$\frac{\partial}{\partial q_m} \langle 0 | \hat{\mathcal{H}} | 0 \rangle = \left[\sum_m \omega_m q_m + \kappa_{00}^m \right] = 0. \tag{S1}$$

The coordinate $q_m^{(0)}$ minimizing the Hamiltonian for each diagonal state I and vibrational

mode m is such

$$q_m^{(0)} = -\frac{\kappa_{00}^m}{\omega_m}. \tag{S2}$$

The Hamiltonian expressed in the new coordinates $\hat{\tilde{q}}_m = \hat{q}_m + \frac{\kappa_{00}^m}{\omega_m}$ reads

$$\begin{aligned}\hat{\mathcal{H}} = &\sum_I \left[E_{II} + \sum_m \frac{1}{2}\omega_m(\hat{p}_m^2 + \hat{\tilde{q}}_m^2 - 2\frac{\kappa_{00}^m}{\omega_m}\hat{\tilde{q}}_m + \frac{(\kappa_{00}^m)^2}{\omega_m^2}) + \sum_m \kappa_{II}^m(\hat{\tilde{q}}_m - \frac{\kappa_{00}^m}{\omega_m})\right] |I\rangle \langle I| \\ &+ \sum_{I>J}\left[E_{IJ} + \sum_m \kappa_{IJ}^m(\hat{\tilde{q}}_m - \frac{\kappa_{00}^m}{\omega_m})\right] |I\rangle \langle J| + h.c.\end{aligned} \tag{S3}$$

After adapting, the equation above becomes

$$\begin{aligned}\hat{\mathcal{H}} = &\sum_I \left[E_{II} + \sum_m \left(\frac{(\kappa_{00}^m)^2}{2\omega_m} - \kappa_{II}^m\frac{\kappa_{00}^m}{\omega_m}\right) + \sum_m \frac{1}{2}\omega_m(\hat{p}_m^2 + \hat{\tilde{q}}_m^2) + \sum_m (\kappa_{II}^m - \kappa_{00}^m)\,\hat{\tilde{q}}_m\right] |I\rangle \langle I| \\ &+ \sum_{I>J}\left[E_{IJ} - \sum_m \kappa_{IJ}^m\frac{\kappa_{00}^m}{\omega_m} + \sum_m \kappa_{IJ}^m\hat{\tilde{q}}_m\right] |I\rangle \langle J| + h.c..\end{aligned} \tag{S4}$$

The diagonal and offdiagonal energies of the Hamiltonian expressed in the new coordinates $\tilde{q}_m$ are thus given by

$$\begin{aligned}\tilde{E}_{II} &= E_{II} + \sum_m \left(\frac{\kappa_{00}^{m\,2}}{2\omega_m} - \kappa_{II}^m\frac{\kappa_{00}^m}{\omega_m}\right) \quad \text{and} \\ \tilde{E}_{IJ} &= E_{IJ} - \sum_m \kappa_{IJ}^m\frac{\kappa_{00}^m}{\omega_m},\end{aligned} \tag{S5}$$

while the diagonal and offdiagonal linear couplings are given by

$$\begin{aligned}\tilde{\kappa}_{II}^m &= \kappa_{II}^m - \kappa_{00}^m \quad \text{and} \\ \tilde{\kappa}_{IJ}^m &= \kappa_{IJ}^m.\end{aligned} \tag{S6}$$

S4.3 Vibrational Modes and Coupling Constants

The vibrational modes used in the quantum dynamical simulations of the full model $\hat{\mathcal{H}}$ as well as their frequencies are presented in Table S3. The linear coupling constants κ of the hermitian Hamiltonian $\hat{\mathcal{H}}$ are presented in Table S4.

Table S3: Vibrational modes and their frequencies (in cm^{-1}) used for the quantum dynamical simulations. The H, B, C, N, F, and Cl atoms are described by the white, pink, gray, blue, green and yellow spheres, respectively. The arrows present the eigenvector of the vibrational mode scaled by a factor of 15.

v022 (102.1 cm^{-1})	v023 (112.14 cm^{-1})	v025 (126.73 cm^{-1})	v026 (132.81 cm^{-1})
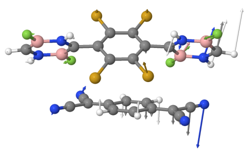	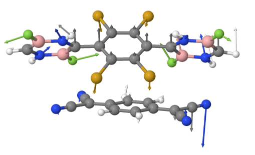	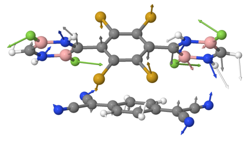	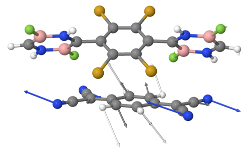
v027 (134.65 cm^{-1})	v029 (145.18 cm^{-1})	v030 (145.81 cm^{-1})	v031 (156.95 cm^{-1})
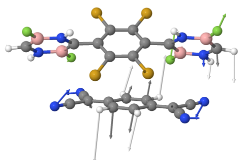	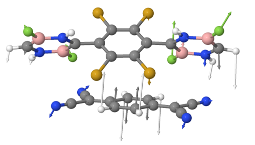	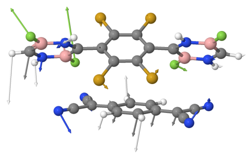	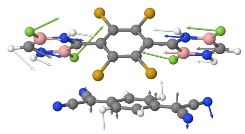
v032 (157.7 cm^{-1})	v033 (179.76 cm^{-1})	v034 (224.14 cm^{-1})	v036 (226.49 cm^{-1})
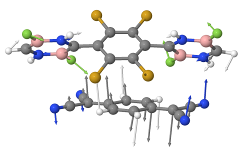	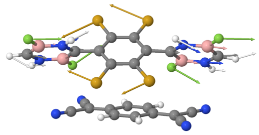	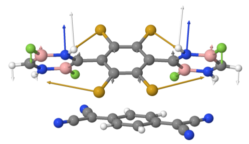	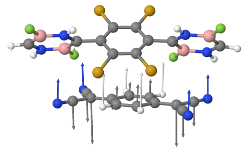
v038 (242.93 cm^{-1})	v039 (255.32 cm^{-1})	v040 (270.03 cm^{-1})	v043 (310.13 cm^{-1})
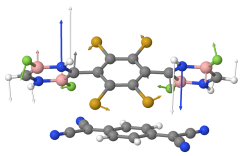	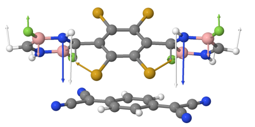	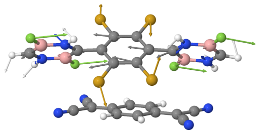	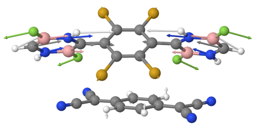
v044 (317.5 cm^{-1})	v045 (332.11 cm^{-1})	v046 (332.98 cm^{-1})	v047 (335.8 cm^{-1})

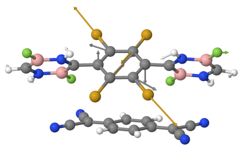
v048 (337.03 cm^{-1})

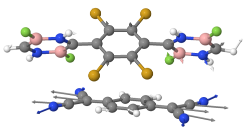
v050 (353.68 cm^{-1})

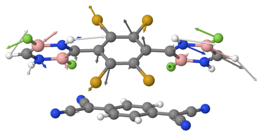
v054 (415.25 cm^{-1})

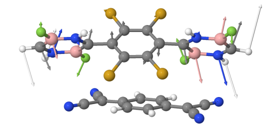
v055 (418.17 cm^{-1})

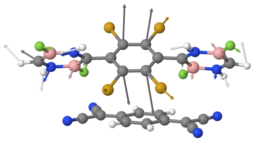
v056 (419.72 cm^{-1})

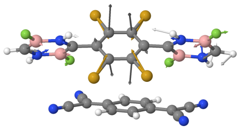
v057 (427.59 cm^{-1})

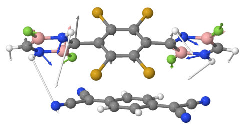
v058 (445.19 cm^{-1})

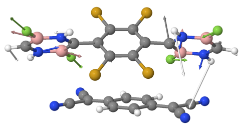
v067 (537.3 cm^{-1})

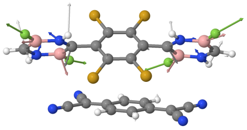
v069 (570.06 cm^{-1})

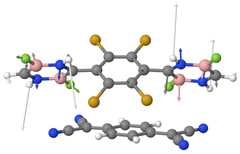
v070 (589.75 cm^{-1})

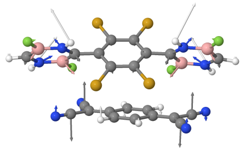
v074 (622.78 cm^{-1})

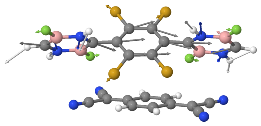
v087(746.47 cm^{-1})

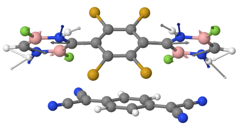
v105 (1045.06 cm^{-1})

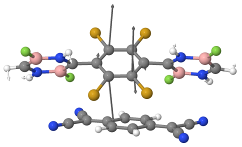
v106 (1122.48 cm^{-1})

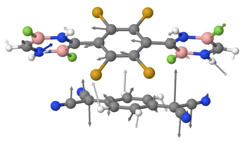
v108 (1161.36 cm^{-1})

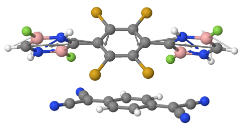
v109 (1179.16 cm^{-1})

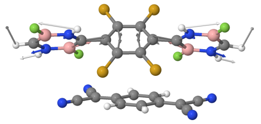
v110 (1199.34 cm^{-1})

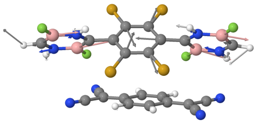
v122 (1326.93 cm^{-1})

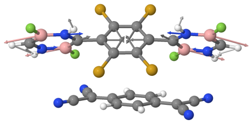
v123 (1339.59 cm^{-1})

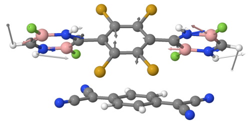
v124 (1343.18 cm^{-1})

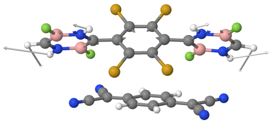
v126 (1368.98 cm^{-1})

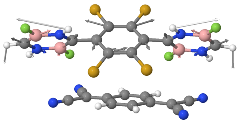
v128 (1424.47 cm^{-1})

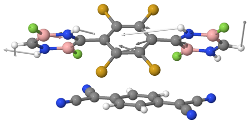
v129 (1437.13 cm^{-1})

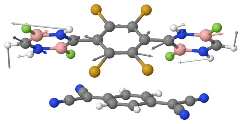
v130 (1484.32 cm^{-1})

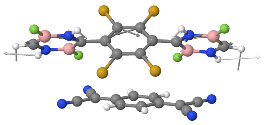 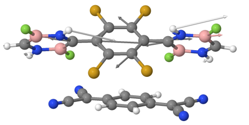 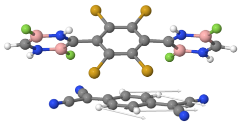 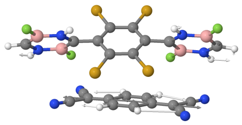

v133 (1527.13 cm^{-1})	v137 (1561.29 cm^{-1})	v139 (1569.96 cm^{-1})	v140 (1583.66 cm^{-1})

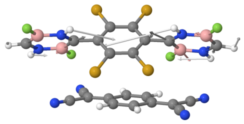 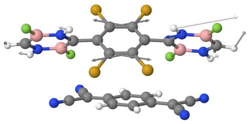 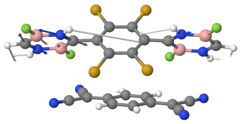 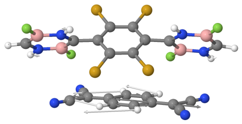

Table S4: Linear coupling constants (in eV) of the vibrational modes used in the quantum dynamical simulations.

mode	v022	v023	v025	v026	v027	v029	v030	v031
$\kappa_{0,0}$	0.0	-0.0	0.0	0.0	0.0	0.0	0.0	0.0
$\kappa_{0,1}$	0.00402	-0.00015	-0.00061	-0.00136	-0.00362	-0.00289	0.00312	-0.00264
$\kappa_{0,2}$	0.00205	0.00201	-0.00051	-7e-05	0.00028	-0.00093	-0.00132	-0.00175
$\kappa_{0,3}$	0.00403	0.00239	-0.0019	-0.00244	0.00114	-0.00047	0.00155	0.00555
$\kappa_{0,4}$	4e-05	5e-05	5e-05	-0.00014	-0.00018	-1e-05	0.00013	-4e-05
$\kappa_{0,5}$	-0.00101	0.00257	-0.00135	0.00309	0.00119	0.00758	-0.02075	-0.00089
$\kappa_{0,6}$	0.00054	0.00088	-0.0002	0.00031	0.00012	-9e-05	9e-05	-0.00017
$\kappa_{0,7}$	0.00288	-0.00048	-0.00135	0.00437	0.01652	0.0124	-0.00628	0.02189
$\kappa_{0,8}$	0.00271	0.00204	-0.00116	0.00068	0.00291	0.00194	-0.00222	-0.00329
$\kappa_{0,9}$	5e-05	-0.00013	0.00028	0.00058	-0.00072	0.00089	0.00071	-0.00013
$\kappa_{0,10}$	-0.00136	-7e-05	-6e-05	0.00109	0.00047	0.00096	8e-05	0.00058
$\kappa_{0,11}$	0.00115	-0.00075	0.00053	-0.00124	-0.00089	2e-05	-0.00071	0.00089
$\kappa_{0,12}$	-0.00294	0.00103	0.00226	-0.00038	-0.00122	-0.00407	-0.00267	-0.01151
$\kappa_{0,13}$	-0.00234	-0.00175	0.00213	-0.00111	-0.00578	-0.0012	0.00935	0.01044
$\kappa_{0,14}$	0.00061	-0.0	-5e-05	-0.00036	-0.00092	-0.00029	0.0008	-0.00039

$\kappa_{0,15}$	-0.00311	-0.00025	-0.0024	0.00405	0.00551	0.00481	-0.00392	0.0014
$\kappa_{0,16}$	-0.00212	-0.00127	0.00021	-0.00108	0.00262	0.00027	0.00246	0.00262
$\kappa_{0,17}$	-0.00018	-0.00011	0.00021	0.0	0.00033	0.00018	-0.00015	0.00024
$\kappa_{0,18}$	-0.00245	9e-05	0.00129	-0.00132	-0.0065	-0.003	0.00402	0.00382
$\kappa_{1,1}$	0.03558	0.02304	-0.01369	-0.00429	-0.01405	-0.02576	-0.01232	-0.00538
$\kappa_{1,2}$	0.0067	0.00472	-0.00285	0.00269	0.00294	-0.00039	0.00179	0.00517
$\kappa_{1,3}$	-0.00139	2e-05	-0.00367	0.00047	-0.00446	-0.00137	0.00472	-0.00258
$\kappa_{1,4}$	-0.00015	-0.00044	-7e-05	0.00069	-0.0002	5e-05	0.00036	-0.00032
$\kappa_{1,5}$	0.00109	0.00124	-0.00094	-0.00036	-0.00083	-0.00096	-9e-05	-0.00197
$\kappa_{1,6}$	5e-05	0.00678	-0.00301	0.00504	0.00449	0.00256	-0.00227	0.00202
$\kappa_{1,7}$	0.00649	0.00706	-0.00092	0.00261	-2e-05	-0.00121	-0.0002	-0.00365
$\kappa_{1,8}$	-0.0008	-0.00053	7e-05	-8e-05	0.00023	0.00028	0.00013	0.00082
$\kappa_{1,9}$	-0.00027	0.00011	0.0001	-0.00027	-5e-05	0.00027	-0.00035	0.00112
$\kappa_{1,10}$	-0.00257	0.00018	0.00138	0.00026	-0.00327	-0.00105	0.00776	-0.00088
$\kappa_{1,11}$	-0.0089	-0.00464	6e-05	-0.00118	-0.00202	-0.00066	-0.00609	-0.00531
$\kappa_{1,12}$	-0.00017	0.00046	-0.00024	0.00135	0.00103	0.00058	-0.00081	0.00031
$\kappa_{1,13}$	0.00234	0.00169	-0.00099	-0.00098	-0.00154	-0.00093	0.00093	-0.00303
$\kappa_{1,14}$	0.00077	0.00017	-0.00082	-0.00026	-0.00066	-0.0005	0.00031	-0.00095
$\kappa_{1,15}$	-0.01625	-0.0093	0.00446	-0.00167	-0.00682	-0.00098	0.00655	-0.02213
$\kappa_{1,16}$	0.00133	5e-05	-0.00019	0.00099	8e-05	-0.00124	0.00148	-0.00143
$\kappa_{1,17}$	-7e-05	9e-05	3e-05	-0.0002	-8e-05	0.00017	-9e-05	0.00021
$\kappa_{1,18}$	0.00034	0.00041	-0.00015	-0.00059	-1e-05	-5e-05	-0.00055	-6e-05
$\kappa_{2,2}$	0.015	0.01433	-0.00195	-0.01263	-0.01819	-0.01069	-0.00738	-0.01158
$\kappa_{2,3}$	0.00029	0.00067	-0.00041	-7e-05	0.00057	-0.00348	-0.00344	-0.00258
$\kappa_{2,4}$	0.00029	0.00167	0.00078	-0.0034	0.00137	-0.00025	-0.00128	0.0007
$\kappa_{2,5}$	-0.00092	-0.00067	0.00068	0.00303	0.00095	0.0012	-0.00063	0.00153

$\kappa_{2,6}$	-8e-05	0.00152	-0.00077	0.00123	0.0009	0.00045	-0.00049	0.00042
$\kappa_{2,7}$	0.00049	-0.00035	-0.00035	-9e-05	-0.00012	0.00021	-0.00029	0.00112
$\kappa_{2,8}$	-0.00027	-0.00047	0.00026	-0.00045	-0.0003	-0.00012	0.00025	0.00033
$\kappa_{2,9}$	-0.00175	-0.00287	0.00034	0.00096	0.00069	-0.00041	-0.00233	-0.00891
$\kappa_{2,10}$	-0.00072	-0.00029	2e-05	0.0001	-4e-05	0.00055	0.00109	-0.00026
$\kappa_{2,11}$	-0.00174	0.00055	0.00048	0.0003	-0.00124	0.00428	0.00556	0.00746
$\kappa_{2,12}$	0.00133	0.00084	0.00011	-0.00058	-0.00072	-0.00026	-0.00096	-0.00142
$\kappa_{2,13}$	-0.00032	-0.00072	-0.0001	-0.00031	0.00038	-0.0004	-0.00095	-0.00062
$\kappa_{2,14}$	-0.0007	0.00016	0.00046	0.00063	0.00164	0.00094	-0.00034	0.00028
$\kappa_{2,15}$	-0.00312	-0.00188	0.00059	-0.00019	-0.00019	0.00091	0.00093	-0.00235
$\kappa_{2,16}$	-0.00473	-0.00125	0.00015	-0.00306	-0.00434	0.00134	-0.00776	0.00758
$\kappa_{2,17}$	-0.00027	-0.00068	0.00052	-0.00025	0.00032	0.00015	-0.0003	0.00018
$\kappa_{2,18}$	0.00057	2e-05	-0.00049	-0.00083	-0.00023	-0.00038	-2e-05	-0.00062
$\kappa_{3,3}$	0.00885	0.00184	-0.00256	-0.00043	0.00223	-0.00231	0.00079	0.01094
$\kappa_{3,4}$	0.0002	0.00022	2e-05	-0.00026	-9e-05	6e-05	0.00023	8e-05
$\kappa_{3,5}$	-0.00063	-0.00142	-0.00022	-0.0006	0.00079	0.00052	-0.0034	-0.00252
$\kappa_{3,6}$	-0.00019	-0.00074	0.00042	-0.00069	-0.0004	-0.00022	0.00026	0.0002
$\kappa_{3,7}$	-0.00543	-0.00179	0.00023	0.00051	0.00083	0.00159	0.00049	0.00138
$\kappa_{3,8}$	0.00053	0.0002	0.00014	5e-05	0.00033	0.00019	-0.00018	-0.00033
$\kappa_{3,9}$	0.00017	0.00014	-5e-05	-0.00022	-0.00015	0.0	-9e-05	0.00017
$\kappa_{3,10}$	0.00055	0.00078	-0.00061	0.00043	0.00012	3e-05	2e-05	-3e-05
$\kappa_{3,11}$	-0.00324	-0.0	-0.00371	-0.00028	-0.0005	0.00373	-0.00494	0.00306
$\kappa_{3,12}$	-0.00125	0.00034	0.00136	0.00031	0.00021	0.00029	-0.00086	-0.00041
$\kappa_{3,13}$	-0.00082	0.00058	-0.00084	-0.00027	-0.00163	-0.00096	0.00078	0.00172
$\kappa_{3,14}$	-0.00044	-0.00059	0.0007	-8e-05	0.00016	0.00075	0.00021	0.00112
$\kappa_{3,15}$	0.00099	0.00222	-0.00095	0.00138	0.00126	0.00073	-0.00102	0.00078

$\kappa_{3,16}$	-0.0002	-0.00032	6e-05	0.00066	-0.00027	0.00016	0.00056	0.00065
$\kappa_{3,17}$	2e-05	3e-05	5e-05	-1e-05	-5e-05	6e-05	6e-05	2e-05
$\kappa_{3,18}$	-9e-05	0.00014	0.0004	-0.00031	-0.00065	-0.00021	0.00051	0.00016
$\kappa_{4,4}$	0.00228	-0.00672	-0.00601	-0.0	5e-05	-0.001	-0.00462	0.01239
$\kappa_{4,5}$	-0.0001	-2e-05	6e-05	6e-05	-0.00022	0.00047	0.00079	0.00056
$\kappa_{4,6}$	0.00045	0.00052	-7e-05	0.00011	0.00045	0.00054	0.00041	0.00055
$\kappa_{4,7}$	0.0	-9e-05	-8e-05	-6e-05	-5e-05	0.00012	0.00021	0.0002
$\kappa_{4,8}$	0.00048	0.00043	4e-05	2e-05	-0.00021	-0.00018	0.00027	-0.00033
$\kappa_{4,9}$	-0.00369	-0.00426	0.00735	-0.00038	0.00678	0.00243	-0.00609	0.00538
$\kappa_{4,10}$	-0.00044	-0.00058	0.0011	-1e-05	0.00101	0.00059	-0.00106	0.00077
$\kappa_{4,11}$	0.00028	0.0001	0.00043	-0.00084	0.00042	-0.00053	5e-05	-0.00041
$\kappa_{4,12}$	-1e-05	-8e-05	9e-05	3e-05	5e-05	-0.00016	-0.00032	-0.00017
$\kappa_{4,13}$	4e-05	0.0	-6e-05	2e-05	-0.00017	0.00012	0.00044	6e-05
$\kappa_{4,14}$	-0.00107	-0.00147	0.00032	-0.00064	0.0006	-0.00383	-0.00889	-0.0071
$\kappa_{4,15}$	0.00012	6e-05	-0.00028	5e-05	-0.0004	3e-05	4e-05	-0.00013
$\kappa_{4,16}$	0.00178	0.00119	0.00149	-0.00098	0.00024	0.00781	0.00089	0.00577
$\kappa_{4,17}$	0.00063	0.00091	0.00021	0.00027	0.00077	0.00108	0.00075	0.00148
$\kappa_{4,18}$	0.00014	0.00011	-0.0002	-8e-05	-0.0	9e-05	0.00014	-5e-05
$\kappa_{5,5}$	0.00093	-0.0102	-0.00646	-0.00439	0.00522	-0.00725	-0.02137	0.00658
$\kappa_{5,6}$	-7e-05	0.0001	-0.00023	0.00011	-1e-05	0.00016	0.0001	-0.00016
$\kappa_{5,7}$	-0.0012	-0.00263	8e-05	-0.00096	-0.0061	-0.00805	-0.00062	0.0109
$\kappa_{5,8}$	0.00108	0.00184	-0.00028	0.00062	0.00137	0.00323	0.00385	0.00262
$\kappa_{5,9}$	7e-05	0.00039	-0.00043	-0.00071	2e-05	-0.00141	-0.00023	-0.00086
$\kappa_{5,10}$	-0.00061	-0.00167	0.00054	-0.00077	0.00305	0.00084	-0.00358	0.00292
$\kappa_{5,11}$	0.00019	0.00026	-0.00046	-0.00011	-3e-05	-0.00049	-0.00025	-0.0007
$\kappa_{5,12}$	-0.0047	0.01023	0.00581	0.00269	-0.00542	0.00656	0.02481	0.01183

$\kappa_{5,13}$	-0.00262	-0.00135	-0.00018	-0.00136	-0.00706	-0.00388	-0.00029	0.00373
$\kappa_{5,14}$	-0.00017	-0.00034	0.00013	-0.0001	0.00037	-0.00014	-0.00054	3e-05
$\kappa_{5,15}$	0.0	-0.00065	0.00084	-0.00123	-0.0026	-0.00206	0.00031	0.00174
$\kappa_{5,16}$	0.00172	0.00065	-0.0007	0.00088	0.00047	0.00106	-0.00123	-0.00193
$\kappa_{5,17}$	-8e-05	-3e-05	-2e-05	-7e-05	0.00012	4e-05	-4e-05	2e-05
$\kappa_{5,18}$	0.00292	0.00332	-0.0022	0.00176	0.0043	-0.00082	-0.00484	-0.00017
$\kappa_{6,6}$	-0.00379	-0.0088	0.00709	-0.00152	-0.00202	-0.00521	0.0003	0.0125
$\kappa_{6,7}$	-0.00094	-0.00041	0.00139	-0.00012	0.00054	0.00027	-0.00049	0.00157
$\kappa_{6,8}$	0.00013	0.00012	-0.00015	6e-05	-7e-05	5e-05	3e-05	-0.00023
$\kappa_{6,9}$	1e-05	-0.00019	0.00024	-5e-05	-0.00022	0.00072	0.00064	0.00062
$\kappa_{6,10}$	-0.00063	0.00094	-0.00322	0.0005	-0.00103	-0.00546	-0.00141	-0.00441
$\kappa_{6,11}$	0.00059	-0.00112	0.00066	-0.00119	-0.00118	-0.00087	0.00088	-0.00073
$\kappa_{6,12}$	-4e-05	-6e-05	1e-05	2e-05	0.00011	5e-05	-5e-05	-8e-05
$\kappa_{6,13}$	0.00013	-0.00015	0.00025	-6e-05	-0.00019	-0.00021	-0.00014	-0.0
$\kappa_{6,14}$	0.00557	0.00287	-0.00089	0.00062	0.0035	0.00639	0.00189	0.0037
$\kappa_{6,15}$	0.00465	0.00545	-0.01038	0.00036	-0.00998	-0.00466	0.00819	-0.00826
$\kappa_{6,16}$	-6e-05	-9e-05	0.00013	5e-05	0.0002	-0.00015	-0.00042	0.0
$\kappa_{6,17}$	-0.00061	-0.00075	-2e-05	-0.00032	-0.00047	-0.00092	-0.00072	-0.00108
$\kappa_{6,18}$	0.00017	2e-05	-0.00013	0.00013	0.00031	-3e-05	-0.00053	-0.00023
$\kappa_{7,7}$	-0.03074	-0.01919	0.01479	-0.00992	-0.03978	-0.04002	0.00984	0.03517
$\kappa_{7,8}$	-0.00069	-0.0009	-0.00022	0.00018	-0.00057	-0.00507	-0.00602	-0.00669
$\kappa_{7,9}$	-0.00084	-0.00081	0.00099	-0.00096	-0.00154	-0.00125	0.00061	0.00027
$\kappa_{7,10}$	0.00269	0.00091	-0.00192	0.00106	0.00256	0.00104	-0.004	-0.00565
$\kappa_{7,11}$	-0.00016	0.00072	-8e-05	0.00085	0.00041	0.00014	-0.00055	-0.00147
$\kappa_{7,12}$	-0.01089	0.00196	0.0084	-0.00166	-0.01722	-0.00456	0.02551	0.01299
$\kappa_{7,13}$	-0.00804	-0.00985	0.00217	-0.00452	-0.00838	-0.00975	-0.0067	-0.00804

$\kappa_{7,14}$	-0.00046	-6e-05	-0.00035	0.00042	0.00072	1e-05	-0.00088	-0.0004
$\kappa_{7,15}$	-0.00726	-0.00697	0.00266	-0.00197	0.00045	-0.00076	-0.00294	0.00111
$\kappa_{7,16}$	-0.00074	0.00018	0.00075	0.00159	-0.00081	0.00234	0.00303	0.00036
$\kappa_{7,17}$	2e-05	0.00021	-0.00022	0.00026	-0.00014	5e-05	0.00027	-0.00036
$\kappa_{7,18}$	-0.00728	-0.00076	0.00472	-0.00201	-0.01084	-0.00113	0.01175	0.00049
$\kappa_{8,8}$	-0.00354	-0.01423	0.00216	-0.00031	-0.00214	-0.00203	8e-05	0.01497
$\kappa_{8,9}$	0.00158	-0.00261	0.00092	-0.00459	-0.00342	-0.00249	0.00297	-0.00117
$\kappa_{8,10}$	0.00108	-0.00032	0.00056	0.00256	-0.00344	-0.00052	0.00073	-0.0014
$\kappa_{8,11}$	-0.0001	1e-05	-2e-05	0.00024	0.00039	0.00013	-0.00029	-4e-05
$\kappa_{8,12}$	0.00476	0.00304	-0.00126	0.00128	0.00499	0.00365	-0.00479	-0.00199
$\kappa_{8,13}$	-0.00299	-0.0033	0.00111	-0.00109	-0.00463	-0.00604	-0.00281	-0.00404
$\kappa_{8,14}$	-4e-05	-3e-05	3e-05	-0.00022	-9e-05	-0.00022	2e-05	-0.00019
$\kappa_{8,15}$	0.00012	0.00067	-0.00014	-0.00126	-0.00125	-0.00185	-0.00043	-0.00013
$\kappa_{8,16}$	0.00111	-0.00117	5e-05	-0.0021	-0.00087	-0.00152	-0.00014	-0.00134
$\kappa_{8,17}$	0.0008	0.00064	-0.00053	5e-05	-0.00077	-0.00021	0.00076	-0.00068
$\kappa_{8,18}$	-0.00387	-0.0022	-0.00083	0.0009	-0.00132	-0.00946	-0.01299	-0.01095
$\kappa_{9,9}$	0.03339	0.01681	-0.01749	-0.004	-0.01663	-0.02495	-0.01357	-0.00988
$\kappa_{9,10}$	0.00585	0.00346	-0.00403	0.00192	0.00166	-0.00082	-0.00092	0.00357
$\kappa_{9,11}$	-0.0013	-0.00078	0.00036	8e-05	-9e-05	0.00059	-0.00025	-0.00104
$\kappa_{9,12}$	0.00012	0.00057	0.00067	0.00114	-0.00164	0.00103	0.00231	0.00109
$\kappa_{9,13}$	-0.0013	-0.00099	0.00017	-0.00104	0.00143	-0.00234	-0.0022	-0.00115
$\kappa_{9,14}$	-0.00142	-0.0012	0.00171	0.0004	0.00134	0.00114	-0.00015	0.00224
$\kappa_{9,15}$	-0.00157	-0.00156	0.00087	-0.00088	-0.00167	-0.0017	-0.00142	-0.00193
$\kappa_{9,16}$	0.0098	0.00692	-0.00373	0.00393	0.00233	-0.00062	0.00321	0.00714
$\kappa_{9,17}$	0.00096	-0.00463	0.00229	-0.00434	-0.00308	-0.00194	0.00133	-9e-05
$\kappa_{9,18}$	-0.0003	-0.00041	-0.00021	-0.0009	0.00075	-0.00147	-0.00125	-0.00111

$\kappa_{10,10}$	0.01103	0.00886	0.00309	-0.01063	-0.01831	-0.01104	-0.00096	-0.01305
$\kappa_{10,11}$	0.00051	-0.00029	-8e-05	8e-05	0.00018	-0.00035	-0.0004	-0.00032
$\kappa_{10,12}$	-0.00149	-0.00078	0.00123	-0.00084	-0.00069	3e-05	0.00126	0.00156
$\kappa_{10,13}$	0.00074	0.00026	-0.00073	-0.0002	0.00014	-0.00015	-0.00099	-0.00169
$\kappa_{10,14}$	0.00017	0.00042	-0.00068	0.00021	-0.00049	-0.00021	0.00032	-0.00016
$\kappa_{10,15}$	0.00789	0.00571	-0.0042	0.0036	0.00379	0.00028	0.00183	0.00361
$\kappa_{10,16}$	0.00158	0.00139	-0.00082	0.00107	-0.0001	0.00069	0.00167	0.0012
$\kappa_{10,17}$	3e-05	-0.00207	-0.00018	0.00216	-0.00131	0.00053	0.00138	-0.00098
$\kappa_{10,18}$	-0.00078	0.00146	0.00084	-0.00012	-0.00444	-0.00049	0.00489	0.00026
$\kappa_{11,11}$	0.0246	0.01622	-0.00719	-0.0067	-0.00956	-0.01224	-0.00488	-0.00955
$\kappa_{11,12}$	-2e-05	0.0004	-1e-05	0.00148	0.00015	9e-05	-0.00048	-0.00045
$\kappa_{11,13}$	-9e-05	0.00034	8e-05	0.00076	0.00142	0.00112	-0.00112	0.00027
$\kappa_{11,14}$	0.00123	-0.0022	-0.0012	-0.00165	-0.00294	-6e-05	0.00039	-0.00186
$\kappa_{11,15}$	0.00314	0.00193	-0.00088	0.0003	0.00017	8e-05	-2e-05	0.00162
$\kappa_{11,16}$	-2e-05	-0.00027	2e-05	-0.00082	-0.00121	-0.00052	0.00075	0.00258
$\kappa_{11,17}$	0.00032	0.00014	-0.0003	-0.00031	-2e-05	0.0003	-0.00022	-0.00012
$\kappa_{11,18}$	-0.00089	-0.00066	7e-05	-0.00031	1e-05	0.00017	8e-05	0.00049
$\kappa_{12,12}$	-0.00378	-0.0028	-0.00455	0.00197	-0.00257	-0.01107	-0.02027	0.00859
$\kappa_{12,13}$	-0.00354	-0.0002	0.00286	-0.00108	-0.00445	0.00016	0.00552	0.0007
$\kappa_{12,14}$	-0.00027	0.00019	0.00016	-0.00013	-0.00062	6e-05	0.00046	0.0001
$\kappa_{12,15}$	-0.0017	-0.00099	0.00184	-0.0014	-0.00341	-0.00252	0.00271	0.00249
$\kappa_{12,16}$	0.00011	-0.00079	-0.00104	-0.00134	0.00099	-0.00235	-0.00345	-0.00137
$\kappa_{12,17}$	-0.00025	-0.00025	0.00024	6e-05	0.00029	0.00015	-0.00026	0.00028
$\kappa_{12,18}$	0.00505	0.00443	-0.00071	0.00093	0.00452	0.00582	6e-05	0.00079
$\kappa_{13,13}$	-0.002	-0.00244	-0.00324	0.00448	0.0042	-0.01685	-0.01632	0.0018
$\kappa_{13,14}$	-0.00046	-0.00063	0.00027	-0.00019	0.00077	0.00063	-0.00134	0.00052

$\kappa_{13,15}$	-0.0039	-0.00362	0.00153	-0.00214	-0.00184	-0.00139	0.00057	0.00099
$\kappa_{13,16}$	-0.00059	-0.00042	0.0	-0.00021	0.00074	5e-05	-0.00012	-2e-05
$\kappa_{13,17}$	0.00026	0.00022	-0.00022	4e-05	-0.00033	-6e-05	0.00028	-0.00022
$\kappa_{13,18}$	-0.0035	-0.00266	0.00155	-0.00144	-0.00603	-0.00319	0.00041	-0.00169
$\kappa_{14,14}$	-0.00329	0.001	-0.00677	-0.00462	-0.00863	-0.01605	-0.00894	0.00164
$\kappa_{14,15}$	-0.00098	-0.00067	0.00121	0.00131	0.00226	0.00197	-0.00056	0.00146
$\kappa_{14,16}$	0.00107	0.00038	-0.00053	-0.00072	-0.00074	-0.00024	0.00082	-0.00054
$\kappa_{14,17}$	0.00363	0.00398	-0.00064	0.00059	0.0031	0.00607	0.00456	0.00586
$\kappa_{14,18}$	-0.00058	0.00012	0.00035	0.00032	6e-05	0.00014	0.0004	0.00033
$\kappa_{15,15}$	0.0272	0.01337	-0.00654	-0.00549	-0.01831	-0.02926	-0.0131	-0.00969
$\kappa_{15,16}$	-0.00075	-0.00022	0.00053	0.00026	5e-05	0.00161	0.00139	0.00011
$\kappa_{15,17}$	0.00013	0.00069	-0.00014	-0.00062	2e-05	-0.00046	-0.00044	-0.00014
$\kappa_{15,18}$	-0.0017	0.0005	0.00135	-0.00108	-0.0034	5e-05	0.00456	0.00107
$\kappa_{16,16}$	0.02143	0.01138	-0.00973	-0.0095	-0.01537	-0.01052	-0.01068	-0.01132
$\kappa_{16,17}$	0.00049	-0.0014	0.00081	-0.00129	-0.00111	-0.00122	0.00056	-0.00025
$\kappa_{16,18}$	-0.0011	-0.00029	0.00032	-0.0009	-0.00052	-0.00153	-0.00075	-0.00042
$\kappa_{17,17}$	-0.00185	-0.01351	-0.00047	-0.00072	-0.00529	-0.00681	-0.00223	0.00989
$\kappa_{17,18}$	-0.00031	-4e-05	0.00027	0.0003	0.00025	0.00028	4e-05	0.00014
$\kappa_{18,18}$	-0.00128	-0.00078	-0.00438	0.00295	0.004	-0.0142	-0.01806	0.00553

mode	v032	v033	v034	v036	v038	v039	v040	v043
$\kappa_{0,0}$	0.0	0.0	0.0	0.0	0.0	0.0	0.0	0.0
$\kappa_{0,1}$	0.00969	-0.00352	0.00097	0.01002	-0.00546	-0.0013	-0.00484	-0.00366
$\kappa_{0,2}$	0.00034	0.00121	-0.00045	-0.00198	0.00229	-0.00021	0.00216	-0.00128
$\kappa_{0,3}$	-0.00145	0.00121	-0.00108	0.00855	0.00338	0.00056	0.00103	0.00171
$\kappa_{0,4}$	-2e-05	4e-05	-1e-05	3e-05	0.0	-6e-05	9e-05	-0.0
$\kappa_{0,5}$	0.00944	0.02204	-0.00761	0.00306	0.0101	-0.00646	0.04428	-0.0495

$\kappa_{0,6}$	-0.00042	6e-05	-0.00018	-9e-05	-0.00015	-0.00013	-0.00019	0.00047
$\kappa_{0,7}$	0.00959	-0.01678	-0.00966	0.00612	-0.00114	0.01216	-0.0294	-0.03576
$\kappa_{0,8}$	0.00248	0.0067	-0.00172	0.00065	0.007	0.00175	0.00608	0.00575
$\kappa_{0,9}$	6e-05	0.00034	-0.0003	1e-05	0.00047	0.00023	-0.00012	0.00107
$\kappa_{0,10}$	0.00095	-0.0003	0.0003	0.00138	-0.0019	-0.0005	0.00075	-3e-05
$\kappa_{0,11}$	-0.00145	-0.00094	0.0004	0.00067	-0.00176	-0.00089	-0.00089	-0.00032
$\kappa_{0,12}$	0.00156	-0.00519	0.0008	-0.00201	0.01162	-0.00478	-0.0114	0.00651
$\kappa_{0,13}$	-0.00367	-0.01824	0.00037	-0.00051	0.00749	0.00056	-0.00896	-0.00403
$\kappa_{0,14}$	0.00014	-0.00047	0.00016	0.00089	-0.00079	-0.00023	-0.00045	0.00026
$\kappa_{0,15}$	0.00591	0.00113	6e-05	0.00417	0.00062	-9e-05	0.00532	0.00081
$\kappa_{0,16}$	0.00084	-0.00189	0.0017	0.00234	-0.00326	0.00136	-0.00224	-0.0002
$\kappa_{0,17}$	-0.00013	1e-05	-0.0001	-0.00021	0.00011	0.00023	0.00012	6e-05
$\kappa_{0,18}$	-0.00134	-0.0113	0.00167	-0.00161	-0.01084	-0.00258	-0.00796	-0.00644
$\kappa_{1,1}$	-0.01282	0.00395	-0.00064	-0.01628	0.00041	-0.00364	-0.01015	-0.02976
$\kappa_{1,2}$	-0.0051	0.00348	-0.00378	0.00308	0.00089	0.00603	-0.00594	0.00465
$\kappa_{1,3}$	0.00469	0.00137	0.00057	0.0074	-0.00179	-0.00132	-0.00163	-0.00036
$\kappa_{1,4}$	-4e-05	-6e-05	0.00016	0.00022	-0.00016	-0.00013	-0.00045	0.00045
$\kappa_{1,5}$	0.00171	0.00012	-0.00015	0.00114	-0.00029	-0.00054	0.00043	0.00067
$\kappa_{1,6}$	-0.00119	0.00024	0.00016	-0.00056	-0.00315	-0.00229	0.00034	0.0019
$\kappa_{1,7}$	-0.00121	0.00019	-0.00197	0.00162	-0.00096	-0.00041	-0.00246	0.00487
$\kappa_{1,8}$	2e-05	-0.00038	0.00016	-0.00039	-0.00042	0.00021	1e-05	-0.00053
$\kappa_{1,9}$	0.00146	-0.00047	7e-05	0.00015	0.00106	-0.00044	0.00333	-0.00381
$\kappa_{1,10}$	-0.00512	0.00521	-0.00035	3e-05	-0.00827	0.00132	0.00329	0.01076
$\kappa_{1,11}$	-0.0032	0.00231	0.00945	-0.00334	-0.00883	-0.01226	0.00827	-0.00453
$\kappa_{1,12}$	-0.00052	0.00053	0.00022	-6e-05	0.00151	-3e-05	0.00049	0.00055
$\kappa_{1,13}$	0.00087	0.00183	-0.00012	0.00352	0.0013	-0.00215	-0.00048	0.0018

$\kappa_{1,14}$	-0.00019	-0.00012	0.00012	0.0004	-0.00195	-0.00031	-0.00097	0.00071
$\kappa_{1,15}$	-0.00249	0.01751	0.00253	-0.00144	-0.00497	-0.00483	0.03071	0.03322
$\kappa_{1,16}$	-0.0034	-0.00598	0.00016	-0.00022	0.0002	0.00126	-0.00944	0.00893
$\kappa_{1,17}$	-0.00023	0.00018	6e-05	-0.00025	-7e-05	-3e-05	6e-05	-7e-05
$\kappa_{1,18}$	-8e-05	0.00105	-0.0004	-0.00067	0.00088	-4e-05	0.00139	-0.00059
$\kappa_{2,2}$	0.01096	-0.01028	-0.00458	-0.0258	-0.00375	0.00119	0.0113	-0.026
$\kappa_{2,3}$	0.00372	-0.00048	-0.00121	-0.00025	0.00033	0.00067	0.00157	-0.00117
$\kappa_{2,4}$	0.00056	0.00066	-0.0008	-0.00114	0.00058	0.0007	0.00238	-0.00165
$\kappa_{2,5}$	4e-05	0.00027	0.00049	-0.00115	-0.00079	0.00099	0.00048	-0.00143
$\kappa_{2,6}$	-0.00027	0.00021	9e-05	0.00014	-0.00095	-0.00065	4e-05	0.00068
$\kappa_{2,7}$	0.00087	-7e-05	0.00041	0.00048	0.00152	7e-05	-0.00012	-0.00256
$\kappa_{2,8}$	-0.0003	-0.0001	4e-05	-0.00049	-0.00016	0.00012	-7e-05	-0.00051
$\kappa_{2,9}$	-0.00283	0.00202	0.0013	-0.00062	-0.00173	-0.00163	-0.00908	0.01255
$\kappa_{2,10}$	-0.0003	0.00238	-0.00049	0.00038	-0.00163	0.00075	0.00111	0.00155
$\kappa_{2,11}$	-0.00286	-0.00133	-0.00391	0.0061	-0.01438	0.0047	0.0007	0.00971
$\kappa_{2,12}$	-0.00047	0.00074	-0.00136	-0.00243	-0.00042	0.0005	0.00173	0.00099
$\kappa_{2,13}$	0.00072	0.0004	-2e-05	-0.00014	0.00069	8e-05	0.00089	-0.00072
$\kappa_{2,14}$	-0.00137	0.00074	0.00027	-0.00065	0.00084	-0.00043	0.001	0.00217
$\kappa_{2,15}$	0.00026	0.00332	0.00019	0.00022	-0.00134	-0.00038	0.00536	0.00305
$\kappa_{2,16}$	0.00666	0.0182	-0.00095	-7e-05	-0.00658	-0.00584	0.03389	-0.03836
$\kappa_{2,17}$	-0.00034	0.00017	-6e-05	-0.00038	0.00031	0.00031	0.0002	-0.00016
$\kappa_{2,18}$	0.00054	0.00027	-2e-05	0.00041	6e-05	-5e-05	0.00029	2e-05
$\kappa_{3,3}$	0.00476	-0.00464	0.00323	0.00606	-0.00178	-0.00235	-0.00417	-0.01707
$\kappa_{3,4}$	8e-05	7e-05	-2e-05	0.00012	7e-05	-5e-05	0.00018	-0.00011
$\kappa_{3,5}$	0.00179	0.00159	0.00025	-0.00351	0.00135	-0.00073	0.00247	-0.00184
$\kappa_{3,6}$	-0.00041	0.0001	6e-05	-0.00047	0.00038	0.00024	-7e-05	-0.00058

$\kappa_{3,7}$	0.00262	-0.00178	0.00019	0.00053	-0.00394	-0.00097	-0.00191	-0.00138
$\kappa_{3,8}$	5e-05	0.00038	-0.00036	-0.00013	0.00129	0.00063	0.00051	0.00045
$\kappa_{3,9}$	8e-05	-0.00025	-4e-05	-0.0001	3e-05	2e-05	0.00012	-0.00046
$\kappa_{3,10}$	0.00013	9e-05	3e-05	0.00035	-0.00022	-0.00018	-0.00012	0.00039
$\kappa_{3,11}$	0.00474	0.00296	-0.00096	0.00191	-0.00362	0.0021	-0.00324	-0.00047
$\kappa_{3,12}$	0.00043	0.00018	0.00069	-0.00058	0.00194	-0.001	-0.00028	-0.00162
$\kappa_{3,13}$	-3e-05	-0.00247	0.002	0.00103	-0.0036	-0.00305	-0.00187	-0.00245
$\kappa_{3,14}$	-0.00117	-0.00017	-0.00025	-0.0007	0.00048	0.00055	9e-05	0.0001
$\kappa_{3,15}$	0.00053	-0.00026	0.00019	7e-05	-0.00113	-0.00074	5e-05	0.00061
$\kappa_{3,16}$	-0.0005	-0.00012	0.00029	6e-05	-2e-05	5e-05	-0.00069	0.00073
$\kappa_{3,17}$	-8e-05	1e-05	-2e-05	3e-05	2e-05	-0.0	1e-05	7e-05
$\kappa_{3,18}$	-0.00031	-0.00074	0.00025	-0.00018	-0.0014	-0.00036	-0.00012	-0.00046
$\kappa_{4,4}$	0.00384	-0.00497	0.00378	-0.00222	-0.0014	-0.00231	-0.00506	-0.01045
$\kappa_{4,5}$	-0.00033	-9e-05	0.00019	0.00024	-0.0003	3e-05	-0.00035	0.00011
$\kappa_{4,6}$	0.0002	0.00041	-0.00055	0.0002	0.00038	0.00089	0.00012	0.00042
$\kappa_{4,7}$	2e-05	4e-05	3e-05	9e-05	-0.00013	8e-05	5e-05	-2e-05
$\kappa_{4,8}$	-0.00031	4e-05	-0.00013	-6e-05	-0.0001	-4e-05	-0.0003	0.00042
$\kappa_{4,9}$	-0.0094	-0.00104	0.00019	-0.01077	0.00153	0.00158	0.00179	0.00037
$\kappa_{4,10}$	-0.00153	-0.00024	-7e-05	-0.00179	0.0004	0.00028	0.00026	0.00018
$\kappa_{4,11}$	-0.0001	-0.00019	0.00015	4e-05	0.00059	-0.0003	0.00068	-0.00062
$\kappa_{4,12}$	2e-05	-6e-05	-3e-05	-0.00022	0.00012	2e-05	3e-05	-3e-05
$\kappa_{4,13}$	0.0001	9e-05	0.0001	0.00037	-3e-05	-0.0001	-0.0001	7e-05
$\kappa_{4,14}$	0.0005	0.00097	0.00365	-0.00235	0.00713	-0.00608	0.00052	-0.00571
$\kappa_{4,15}$	9e-05	0.00016	0.00011	0.00024	1e-05	-0.00014	-0.00018	0.0
$\kappa_{4,16}$	-0.00307	0.00151	-0.00156	-0.00193	0.00326	0.00265	0.0009	0.00177
$\kappa_{4,17}$	0.00014	0.00011	-0.00091	0.0001	-0.00017	0.00141	0.00014	0.00056

$\kappa_{4,18}$	0.00029	0.0002	3e-05	0.00043	4e-05	2e-05	1e-05	-4e-05
$\kappa_{5,5}$	0.01923	0.00427	0.01371	-0.0072	0.014	-0.0205	0.02493	-0.05018
$\kappa_{5,6}$	0.00022	4e-05	-0.0	0.00039	-7e-05	-2e-05	-9e-05	0.00023
$\kappa_{5,7}$	-0.01097	-0.00692	0.0095	-0.00165	-0.01019	-0.01109	-0.01215	-0.02827
$\kappa_{5,8}$	0.00102	0.00107	-0.00399	0.0017	-0.00128	0.00574	0.00145	0.00548
$\kappa_{5,9}$	0.00075	-0.00123	0.00056	0.00147	-0.00183	-0.00084	-0.00111	-1e-05
$\kappa_{5,10}$	-0.00312	0.00367	0.00017	-0.00461	0.00594	0.00083	0.00359	-0.00347
$\kappa_{5,11}$	0.00102	0.00077	-4e-05	-0.0002	0.00124	0.00021	0.00024	-0.00025
$\kappa_{5,12}$	-0.00388	-0.0167	-0.01791	0.00234	-0.02095	0.02618	-0.02003	0.03116
$\kappa_{5,13}$	-0.00414	-0.00808	0.0057	-0.00174	-0.01134	-0.00886	-0.00375	-0.01737
$\kappa_{5,14}$	1e-05	0.00031	-9e-05	-0.00054	0.00048	0.0002	0.00042	-0.0004
$\kappa_{5,15}$	-0.00276	-0.00063	0.00088	-0.00241	-0.00158	-0.00108	-0.00092	-0.00451
$\kappa_{5,16}$	-0.00154	0.00201	-0.00144	-0.00271	0.00356	0.0013	0.00169	0.00301
$\kappa_{5,17}$	7e-05	0.00018	-7e-05	0.00014	4e-05	0.00012	0.00014	5e-05
$\kappa_{5,18}$	-0.00105	0.01172	0.00016	0.00184	0.01147	0.00028	0.00743	-0.00068
$\kappa_{6,6}$	0.00421	-0.00557	0.00316	0.00277	-0.00207	-0.0017	-0.00376	-0.01161
$\kappa_{6,7}$	-0.00245	-0.00109	1e-05	-0.00241	-0.0011	8e-05	-0.00079	-0.00034
$\kappa_{6,8}$	0.00019	0.00024	-5e-05	0.00019	0.00014	-5e-05	0.00019	0.00017
$\kappa_{6,9}$	-0.00077	0.00021	3e-05	-0.00016	9e-05	7e-05	2e-05	0.00026
$\kappa_{6,10}$	0.00543	-0.00051	0.0001	0.00336	-0.00339	-0.00101	-0.00144	-0.00103
$\kappa_{6,11}$	-0.00041	-0.0001	0.00021	-0.00019	0.00042	1e-05	0.00021	-0.00068
$\kappa_{6,12}$	0.00011	8e-05	-1e-05	9e-05	0.0002	7e-05	0.00021	5e-05
$\kappa_{6,13}$	-0.0002	-0.00017	-6e-05	-0.00026	0.00016	6e-05	-7e-05	-0.00019
$\kappa_{6,14}$	0.00558	-0.00429	-0.00427	0.00131	0.00565	0.00633	-0.00298	0.00461
$\kappa_{6,15}$	0.01394	0.00092	-0.0005	0.01442	-0.0029	-0.00188	-0.00386	-0.0005
$\kappa_{6,16}$	-0.00053	-0.00017	0.00017	-0.00049	0.00018	-0.00022	-5e-05	-0.0003

$\kappa_{6,17}$	-0.00046	5e-05	0.00106	-0.00032	0.00026	-0.00147	-0.0001	-0.00059
$\kappa_{6,18}$	6e-05	0.0007	-2e-05	-0.0	0.00082	-7e-05	0.00057	-8e-05
$\kappa_{7,7}$	-0.02017	-0.05472	0.03752	-0.0123	-0.06675	-0.0508	-0.05342	-0.09744
$\kappa_{7,8}$	-0.00157	0.0033	0.00467	-0.00098	0.00501	-0.00675	0.00257	-0.0024
$\kappa_{7,9}$	-0.00166	-0.00135	0.00069	-0.00058	-0.00148	-0.00088	-0.00128	-0.00124
$\kappa_{7,10}$	0.00632	0.00626	-0.00031	0.00208	0.00756	-0.00067	0.00884	0.00143
$\kappa_{7,11}$	-0.00018	0.00078	0.0003	-0.00022	4e-05	-0.00111	0.00122	0.00236
$\kappa_{7,12}$	-0.0182	-0.02322	-0.00343	-0.00205	-0.03592	0.00245	-0.02573	0.00893
$\kappa_{7,13}$	0.00047	-0.00337	0.01778	-0.00104	-0.00505	-0.01933	0.00858	-0.01914
$\kappa_{7,14}$	0.0002	0.00098	0.00032	-0.00034	0.00059	-0.00055	0.00105	-6e-05
$\kappa_{7,15}$	-0.00403	0.00289	0.00374	-0.00629	0.00472	-0.00356	0.00497	-0.00517
$\kappa_{7,16}$	-0.001	0.0005	-0.0004	0.00016	-0.00047	0.00056	0.00018	0.00304
$\kappa_{7,17}$	0.00028	0.00021	4e-05	0.00037	-4e-05	-0.00017	6e-05	0.00039
$\kappa_{7,18}$	-0.0016	-0.01971	-0.00189	-0.00303	-0.02224	0.00135	-0.01659	0.00513
$\kappa_{8,8}$	0.0069	-0.0089	0.00141	0.00037	-0.00384	-0.00072	-0.00787	-0.00361
$\kappa_{8,9}$	6e-05	0.00028	0.00023	-0.00087	0.00178	0.00113	-0.00189	-0.00296
$\kappa_{8,10}$	-0.00138	-0.00133	-0.00058	-0.00066	0.0002	-4e-05	-0.00245	0.00135
$\kappa_{8,11}$	7e-05	0.00037	7e-05	-0.00014	0.00035	8e-05	0.00048	-5e-05
$\kappa_{8,12}$	0.00605	0.00047	-0.00199	5e-05	0.01147	0.00317	5e-05	0.00126
$\kappa_{8,13}$	-0.00141	-0.00798	0.00569	-0.00298	-0.00194	-0.00844	-0.00678	-0.00546
$\kappa_{8,14}$	-9e-05	2e-05	7e-05	-0.00012	6e-05	-5e-05	-9e-05	-0.00015
$\kappa_{8,15}$	-0.00099	-0.00113	0.00066	-0.00088	-0.00045	-0.00128	-0.00081	-0.00236
$\kappa_{8,16}$	0.00116	-0.00033	1e-05	-0.00011	0.00166	0.00045	-0.00044	-0.00153
$\kappa_{8,17}$	0.00078	0.00026	-9e-05	0.00108	-0.00012	-0.00017	-0.00018	0.00024
$\kappa_{8,18}$	-0.00258	0.01074	0.00584	-0.00166	0.00722	-0.00912	0.0101	-0.00935
$\kappa_{9,9}$	-0.00779	0.00319	0.00072	-0.0178	-0.00154	-0.00648	-0.00842	-0.01798

$\kappa_{9,10}$	-0.00326	0.00245	0.00028	0.001	0.00149	-1e-05	-0.00351	-0.00243
$\kappa_{9,11}$	-0.00046	-0.0002	0.0008	-0.00028	0.00051	-0.00128	0.00042	0.00119
$\kappa_{9,12}$	-0.0008	-0.00015	-0.00077	-0.00078	-0.00056	0.00117	-8e-05	0.00074
$\kappa_{9,13}$	0.00019	-0.00077	0.00044	-0.00012	-0.00137	-0.00057	-0.00029	-0.00119
$\kappa_{9,14}$	-0.00138	-0.00018	-0.00064	-0.00137	-0.00071	0.00137	0.00091	0.0009
$\kappa_{9,15}$	-0.00036	-0.00093	0.00193	-0.00126	0.00023	-0.00273	-0.00024	-0.00238
$\kappa_{9,16}$	-0.00722	0.00343	-0.00681	0.00451	0.00243	0.00958	-0.00934	0.00617
$\kappa_{9,17}$	0.00062	-0.00116	-0.00018	0.00019	0.00267	0.0019	-0.00177	-0.00382
$\kappa_{9,18}$	-1e-05	7e-05	0.00033	7e-05	0.00064	-0.00085	-0.00053	-0.00065
$\kappa_{10,10}$	0.0156	-0.00969	-0.00715	-0.017	-0.00464	0.00483	0.00817	-0.00558
$\kappa_{10,11}$	0.00038	-0.00061	0.0002	-0.0002	0.00147	0.00028	-0.00099	-0.00126
$\kappa_{10,12}$	-0.00339	-0.00295	-0.00019	-0.00376	-0.00237	0.00079	-0.00316	0.00074
$\kappa_{10,13}$	0.00259	0.00042	0.00029	0.00157	0.0007	-0.00101	0.00169	-7e-05
$\kappa_{10,14}$	0.0005	0.00095	0.00029	0.00071	-0.00016	-0.00061	0.00056	-0.0005
$\kappa_{10,15}$	-0.00224	0.00536	-0.00332	0.00746	0.00036	0.00474	-0.00337	0.00521
$\kappa_{10,16}$	-0.00082	0.00085	-0.00044	0.00133	0.00019	0.00072	-0.0012	0.00113
$\kappa_{10,17}$	-0.00077	-0.00096	0.00049	0.00081	0.00029	0.00015	-0.00372	0.00268
$\kappa_{10,18}$	0.00034	-0.0084	-0.001	-1e-05	-0.01004	0.00019	-0.00666	0.00167
$\kappa_{11,11}$	-0.00163	-0.00396	-0.00973	-0.01794	-0.00433	0.00114	-0.00186	-0.01314
$\kappa_{11,12}$	0.00037	0.00029	-0.00044	-0.0004	-0.00022	0.00052	0.00055	0.00107
$\kappa_{11,13}$	3e-05	0.00071	-0.00052	-0.00054	0.00067	0.00077	0.00096	0.00111
$\kappa_{11,14}$	0.00071	-0.00049	0.00094	0.00281	-0.00179	-0.00052	-0.00261	7e-05
$\kappa_{11,15}$	0.00199	-0.00175	-0.00211	0.00092	0.00378	0.00367	-0.00168	-0.00308
$\kappa_{11,16}$	0.0001	0.00173	-0.00121	0.0001	-0.00562	0.00227	0.00194	-0.00333
$\kappa_{11,17}$	0.00015	0.00017	6e-05	0.00028	-0.00019	-8e-05	0.00022	9e-05
$\kappa_{11,18}$	0.00096	-0.00087	0.00042	0.00039	-0.00075	3e-05	-0.00024	-0.00094

$\kappa_{12,12}$	0.00761	-0.00499	0.01446	-0.0045	0.00663	-0.01411	-0.00233	-0.03652
$\kappa_{12,13}$	0.00128	-0.00935	-0.00167	-0.00022	-0.00946	0.00243	-0.00841	0.00244
$\kappa_{12,14}$	-0.0004	-0.00082	1e-05	9e-05	-0.00087	-0.00023	-0.00065	0.00045
$\kappa_{12,15}$	-0.00523	-0.00322	0.00033	-0.00288	-0.00373	-0.00056	-0.00501	-0.00212
$\kappa_{12,16}$	0.00059	0.00047	0.0018	0.00164	0.00113	-0.00289	0.0001	-0.00274
$\kappa_{12,17}$	-9e-05	3e-05	1e-05	-0.00033	0.00037	0.0001	0.00018	-0.00026
$\kappa_{12,18}$	0.00963	-0.00016	-0.0056	0.00043	0.01394	0.00884	0.00446	0.00801
$\kappa_{13,13}$	-0.00463	0.01889	0.00929	0.0045	0.01944	-0.01053	0.00638	-0.0186
$\kappa_{13,14}$	-0.00073	0.00099	2e-05	-0.00107	0.00105	0.00014	0.00129	2e-05
$\kappa_{13,15}$	-0.00035	-0.0018	0.00377	0.00014	-0.00309	-0.00389	-0.00058	-0.00456
$\kappa_{13,16}$	1e-05	-0.00017	-1e-05	-0.00024	-3e-05	0.00029	9e-05	0.00116
$\kappa_{13,17}$	0.00013	-3e-05	8e-05	0.00034	-0.00018	-0.00021	-0.00019	0.0002
$\kappa_{13,18}$	-0.00141	-0.01128	0.00364	-0.00407	-0.00758	-0.00673	-0.01003	-0.00695
$\kappa_{14,14}$	0.00411	-0.00135	0.01502	0.0011	-0.00368	-0.01944	-0.00334	-0.03285
$\kappa_{14,15}$	0.00065	-0.0008	-0.00098	-0.00077	0.0009	0.00136	-1e-05	0.00136
$\kappa_{14,16}$	-0.00016	6e-05	-0.00013	0.0002	-0.0004	2e-05	-0.00039	-8e-05
$\kappa_{14,17}$	0.00282	0.00019	-0.00531	0.0019	0.00076	0.00768	-0.0002	0.00454
$\kappa_{14,18}$	-0.00077	-0.00173	-0.00015	-0.00037	-0.00188	8e-05	-0.00139	0.00112
$\kappa_{15,15}$	-0.00467	0.00329	0.00345	-0.01235	0.00054	-0.00974	-0.00726	-0.02455
$\kappa_{15,16}$	0.00082	-0.00096	-0.00046	6e-05	-0.00015	0.00087	0.00017	0.00238
$\kappa_{15,17}$	0.00065	1e-05	-6e-05	2e-05	0.00035	-0.00021	0.00054	-0.00096
$\kappa_{15,18}$	0.00025	-0.0072	-0.00045	1e-05	-0.00863	0.00049	-0.00559	0.00103
$\kappa_{16,16}$	0.01255	-0.00715	-0.00199	-0.02697	0.00286	-0.0009	0.00604	-0.01594
$\kappa_{16,17}$	0.00023	-0.00082	-0.00015	-5e-05	0.00117	0.00069	-0.00102	-0.00146
$\kappa_{16,18}$	-0.00027	-0.00397	3e-05	-0.00102	-0.00372	-0.00076	-0.00263	-0.00021
$\kappa_{17,17}$	0.00838	-0.00554	0.00452	0.00247	-0.00247	-0.00449	-0.00548	-0.00623

$\kappa_{17,18}$	0.00012	-6e-05	-5e-05	-0.00012	3e-05	0.00016	0.00019	-0.0
$\kappa_{18,18}$	0.00223	0.01774	0.00642	-0.00153	0.01219	0.00242	0.01385	-0.02372

mode	v044	v045	v046	v047	v048	v050	v054	v055
$\kappa_{0,0}$	0.0	0.0	0.0	0.0	0.0	0.0	0.0	0.0
$\kappa_{0,1}$	-0.00225	0.00055	0.00712	-0.00621	-0.00035	-5e-05	0.00276	-0.00441
$\kappa_{0,2}$	0.0005	-0.00254	-0.00147	-0.00275	0.00247	-0.00388	0.00071	0.00374
$\kappa_{0,3}$	0.00045	-0.00193	0.00356	-0.00184	0.00162	0.00641	-0.00514	-0.00746
$\kappa_{0,4}$	-4e-05	-6e-05	-3e-05	0.00031	4e-05	0.00013	-0.00029	-3e-05
$\kappa_{0,5}$	0.00437	-0.0006	-0.03235	-0.01876	0.04104	0.01917	-0.00302	0.01951
$\kappa_{0,6}$	-4e-05	-0.00026	0.00027	-8e-05	-0.00033	-0.00012	0.00066	0.00022
$\kappa_{0,7}$	0.01292	-0.00259	0.02421	-0.00032	-0.02171	-0.00433	0.0103	0.00944
$\kappa_{0,8}$	0.00282	-0.00521	-0.00947	-0.00596	-0.00018	-0.00082	-0.00261	0.0035
$\kappa_{0,9}$	0.00023	-0.00012	5e-05	0.00011	-0.00046	5e-05	0.00019	0.00014
$\kappa_{0,10}$	-0.00014	0.00193	-0.00121	0.00035	-0.00051	0.0001	0.00096	0.0001
$\kappa_{0,11}$	-0.00064	-0.00089	0.00203	0.00173	0.00118	-0.00346	0.00104	-0.00115
$\kappa_{0,12}$	-0.00115	-0.00199	0.01654	-0.00351	0.03511	0.02537	-0.03465	-0.00349
$\kappa_{0,13}$	-0.00394	0.00526	0.00628	-0.0194	-0.02443	-0.0134	0.01068	0.02096
$\kappa_{0,14}$	-0.00029	3e-05	0.00134	0.00032	-0.00024	-0.00021	0.00017	-0.00017
$\kappa_{0,15}$	0.00288	0.00274	-0.00709	-0.00141	-0.00045	0.00013	-0.00051	0.00557
$\kappa_{0,16}$	-0.00074	0.0014	0.00157	0.00125	-0.00463	-0.0	0.00273	-0.001
$\kappa_{0,17}$	0.00011	0.00023	-0.00043	1e-05	-0.00035	-0.00022	-6e-05	-4e-05
$\kappa_{0,18}$	-0.00404	0.00258	0.00977	0.00683	-0.00063	0.00108	0.00521	-0.00051
$\kappa_{1,1}$	-0.00681	-0.03896	0.02993	0.00492	0.00846	0.00589	0.03729	-0.0154
$\kappa_{1,2}$	0.00128	-0.00436	-0.00125	-0.00054	-0.01752	-0.00701	0.01731	-0.01238
$\kappa_{1,3}$	0.00027	-0.00119	0.00224	-0.00424	-0.00246	0.00094	0.00031	0.00242
$\kappa_{1,4}$	-0.00023	-0.0	0.00062	-0.00065	0.00035	0.00017	0.00112	-0.00034

$\kappa_{1,5}$	-0.00014	-0.00088	0.00086	-0.00067	0.0012	0.00084	-0.00157	0.00045
$\kappa_{1,6}$	0.00032	0.00229	-0.00156	-0.00021	-0.00134	-0.00177	0.00637	0.00304
$\kappa_{1,7}$	-0.00061	-0.00262	0.00313	-0.00402	-0.00373	-0.00333	0.00581	0.00399
$\kappa_{1,8}$	-2e-05	0.00035	-0.00028	0.00098	-0.00025	-0.00038	0.00013	0.0002
$\kappa_{1,9}$	8e-05	-0.00074	-0.0036	-0.00137	-0.00024	-0.00089	0.00095	0.00269
$\kappa_{1,10}$	-0.00273	-0.00104	-0.0131	0.00533	-0.01069	-0.00406	0.00916	-0.0091
$\kappa_{1,11}$	-0.00612	0.00926	0.00577	0.01786	0.02642	0.0166	-0.01603	-0.01115
$\kappa_{1,12}$	0.00057	-0.00021	-0.0015	-0.00024	0.00023	0.00036	0.00046	0.00136
$\kappa_{1,13}$	-9e-05	-0.00061	0.00139	-0.00263	0.00225	0.00501	-0.00322	-0.00326
$\kappa_{1,14}$	-0.00047	-0.00115	0.00207	0.00041	-0.00013	-0.00081	0.00165	-0.00135
$\kappa_{1,15}$	-0.00558	-0.00025	-0.02243	0.00492	0.02368	0.01197	-0.00769	-0.00743
$\kappa_{1,16}$	-0.00062	0.00077	0.00608	0.00086	-0.00072	0.00165	-0.00243	-0.00144
$\kappa_{1,17}$	-2e-05	0.00025	-0.00027	0.00033	0.00015	0.00017	-4e-05	-0.0
$\kappa_{1,18}$	0.00025	-2e-05	-0.00142	5e-05	0.00011	-0.00068	-0.00139	0.00036
$\kappa_{2,2}$	-0.00111	-0.04249	0.00625	-0.00192	0.00675	0.00049	0.00948	0.05214
$\kappa_{2,3}$	0.00114	-0.00012	-0.00058	-0.00485	0.00072	-0.00133	-0.00205	0.00121
$\kappa_{2,4}$	0.00093	0.00012	-0.00289	0.00327	-0.00149	-0.00073	-0.00598	0.00084
$\kappa_{2,5}$	-8e-05	0.00042	-0.00134	-0.00034	-0.00054	-0.00207	0.00597	0.00192
$\kappa_{2,6}$	-4e-05	0.00043	-0.00044	-3e-05	-0.00037	-0.00042	0.00168	0.00047
$\kappa_{2,7}$	0.00041	0.00013	-0.00013	-0.0005	0.00077	-0.00017	-0.00033	-0.00018
$\kappa_{2,8}$	-0.0001	0.00014	0.00012	0.00039	-0.00023	-0.00029	-6e-05	-0.00029
$\kappa_{2,9}$	-0.00031	0.00553	0.01749	0.00437	0.01484	0.01026	-0.01253	-0.00769
$\kappa_{2,10}$	0.00018	-0.00053	-0.00175	0.00094	-0.00179	-0.00077	0.00176	-0.00189
$\kappa_{2,11}$	0.00115	-0.0019	-0.01102	0.0188	-0.01797	-0.0097	0.01492	0.00514
$\kappa_{2,12}$	5e-05	-0.00122	-0.0008	-1e-05	4e-05	-0.00346	9e-05	0.00308
$\kappa_{2,13}$	0.00035	-0.0001	-0.00082	-0.00112	0.001	0.00028	-0.00096	4e-05

$\kappa_{2,14}$	-0.0	0.00041	-0.0011	-0.00048	0.0012	-0.00059	0.00028	0.00035
$\kappa_{2,15}$	3e-05	-0.00055	-0.00408	0.0016	0.00226	0.00144	-0.00108	-0.00138
$\kappa_{2,16}$	-0.00127	0.00161	-0.02098	-0.00126	0.00991	-0.00073	0.01279	0.00504
$\kappa_{2,17}$	4e-05	-5e-05	-3e-05	0.00054	0.00015	3e-05	-0.00064	-0.00031
$\kappa_{2,18}$	7e-05	-0.00097	0.0002	-0.00065	0.00047	-0.00022	-0.00043	0.00039
$\kappa_{3,3}$	-0.00119	-0.03246	0.01439	0.00391	-0.00355	-0.00805	0.01002	0.00328
$\kappa_{3,4}$	-5e-05	-0.00017	-0.0001	9e-05	-3e-05	-0.0001	-0.00013	-0.00011
$\kappa_{3,5}$	0.0004	0.00069	-0.00183	-0.0022	0.00464	0.00065	-0.00278	0.00271
$\kappa_{3,6}$	-1e-05	7e-05	-4e-05	8e-05	0.00026	0.00041	-0.00048	-0.00089
$\kappa_{3,7}$	-0.00025	0.00061	0.00253	0.00015	-0.00229	-0.0023	0.00279	0.00217
$\kappa_{3,8}$	0.00037	-0.00077	-0.00136	-0.00061	-0.00041	-5e-05	-0.00023	0.00076
$\kappa_{3,9}$	-6e-05	0.00021	-0.00047	0.00026	-0.00024	-0.00019	9e-05	5e-05
$\kappa_{3,10}$	4e-05	0.00018	-0.00046	-0.00063	-0.0005	6e-05	0.00051	0.00026
$\kappa_{3,11}$	0.0029	-0.0006	0.00428	-0.00384	-0.00157	0.00075	0.0029	0.0021
$\kappa_{3,12}$	0.00027	0.0	-0.00041	-0.00087	0.00224	0.00054	-0.00015	0.00239
$\kappa_{3,13}$	-0.00125	0.00171	0.00471	0.00098	0.00193	0.00132	-0.00014	-0.00165
$\kappa_{3,14}$	0.00032	0.00035	-0.00075	0.00032	-0.0006	-0.00024	-0.00033	0.00048
$\kappa_{3,15}$	-9e-05	0.00076	-0.00079	-0.0011	-0.00085	-0.00077	0.00249	0.00058
$\kappa_{3,16}$	-0.0001	-7e-05	0.0008	-0.00043	-0.00012	9e-05	0.00113	0.00011
$\kappa_{3,17}$	-2e-05	-4e-05	-5e-05	5e-05	-4e-05	3e-05	-1e-05	3e-05
$\kappa_{3,18}$	-0.00038	0.00035	0.00057	0.00071	-0.00019	-0.00076	0.00013	-0.00015
$\kappa_{4,4}$	-0.00028	-0.02094	0.01787	0.00815	0.00439	0.00617	0.01028	0.04834
$\kappa_{4,5}$	-0.00018	0.00029	8e-05	0.00058	-0.00049	0.0001	0.00082	-0.0002
$\kappa_{4,6}$	0.00042	-0.00095	-0.00115	-0.0005	-0.00282	-0.0017	0.00151	0.00051
$\kappa_{4,7}$	3e-05	-9e-05	-0.00018	0.00013	-0.00045	-0.00023	0.00033	5e-05
$\kappa_{4,8}$	-0.00011	-0.00018	0.00043	-0.00025	-0.00019	-3e-05	0.00032	-5e-05

$\kappa_{4,9}$	-0.00102	0.0047	-0.00315	0.00707	0.00459	0.00038	9e-05	-0.00516
$\kappa_{4,10}$	-0.00017	0.00072	-0.00026	0.00097	0.00106	0.00018	0.00018	-0.00068
$\kappa_{4,11}$	-0.00017	-0.0002	-0.00059	0.00062	0.00089	0.00038	-0.00218	-0.00017
$\kappa_{4,12}$	4e-05	-0.0	6e-05	-4e-05	0.00022	-1e-05	-0.00026	-5e-05
$\kappa_{4,13}$	-2e-05	-2e-05	-3e-05	-2e-05	-0.00017	0.00021	5e-05	4e-05
$\kappa_{4,14}$	-0.00127	0.00375	0.00893	-0.00611	0.02722	0.0178	-0.01942	-0.00477
$\kappa_{4,15}$	-4e-05	-0.00014	0.00039	-0.00023	0.00035	0.00033	0.00041	-0.00011
$\kappa_{4,16}$	0.00089	0.00053	-0.006	0.00315	-0.00293	-0.00316	0.00715	-0.00041
$\kappa_{4,17}$	0.00059	-0.00113	-0.00229	-0.00034	-0.00484	-0.00311	0.0032	0.001
$\kappa_{4,18}$	8e-05	-0.00011	4e-05	-0.00031	-2e-05	7e-05	2e-05	0.0002
$\kappa_{5,5}$	-0.00029	0.01063	-0.00234	-0.0193	0.04262	0.02541	-0.01564	0.04752
$\kappa_{5,6}$	8e-05	-9e-05	6e-05	-0.00019	-0.00014	0.00023	-5e-05	0.00037
$\kappa_{5,7}$	-0.00878	0.0114	0.02116	0.0129	0.0027	-0.01512	0.01373	-0.01144
$\kappa_{5,8}$	0.00212	-0.00451	-0.00793	0.00084	-0.01304	-0.00525	0.00811	0.00238
$\kappa_{5,9}$	-0.00072	0.0004	0.00203	0.0007	-9e-05	0.00179	-0.00053	-0.00142
$\kappa_{5,10}$	0.00162	-0.00159	-0.00548	-0.00179	0.00221	-0.00234	-0.00206	0.00142
$\kappa_{5,11}$	0.00011	-0.00089	-0.0006	-0.00091	0.00098	0.00029	-0.00118	-0.00034
$\kappa_{5,12}$	0.00202	-0.00754	0.00032	0.01689	-0.05742	-0.0065	0.02056	-0.00083
$\kappa_{5,13}$	-0.00628	0.00816	0.01371	0.01313	0.01021	-0.00406	-0.00421	-0.00067
$\kappa_{5,14}$	0.00019	-0.00046	-0.00062	-0.00023	1e-05	1e-05	-0.00022	-0.00032
$\kappa_{5,15}$	-0.00188	-0.0	0.00269	0.00339	0.00098	-0.00235	0.00218	-0.00293
$\kappa_{5,16}$	0.00171	-0.00359	-0.00206	-0.00334	0.00082	-0.00352	0.00047	0.00481
$\kappa_{5,17}$	0.0001	-0.00014	-0.00025	-0.0001	-0.00013	0.00016	-6e-05	0.00013
$\kappa_{5,18}$	0.00205	-0.00561	-0.0101	-0.00365	-0.00045	0.00115	-0.00013	-0.00514
$\kappa_{6,6}$	-0.00565	-0.0247	0.01387	0.00276	0.00436	-0.00311	0.04396	-0.0114
$\kappa_{6,7}$	-0.00067	0.00109	0.00072	0.00249	0.00015	-0.00082	0.00112	-0.00172

$\kappa_{6,8}$	0.00012	-0.00017	-0.0002	-0.00022	-3e-05	4e-05	-0.0002	0.00033
$\kappa_{6,9}$	-0.00015	7e-05	-0.0005	0.0009	-0.00036	-0.00016	0.00088	-0.00027
$\kappa_{6,10}$	0.00056	-0.00149	0.00468	-0.00517	-0.00021	0.00111	-0.00388	0.00151
$\kappa_{6,11}$	-0.00042	-0.00029	0.00046	0.00075	0.00117	0.00101	-0.00124	-0.00141
$\kappa_{6,12}$	8e-05	-8e-05	-0.00026	-0.00013	5e-05	5e-05	-0.00033	0.00023
$\kappa_{6,13}$	-0.00011	0.0002	9e-05	0.00036	1e-05	8e-05	-0.0002	-0.00068
$\kappa_{6,14}$	0.00568	-0.00657	-0.00562	-0.01336	-0.02268	-0.01639	0.00855	0.01521
$\kappa_{6,15}$	0.00162	-0.00763	0.00703	-0.01042	-0.00645	-0.00077	-0.00129	0.00657
$\kappa_{6,16}$	-0.00018	0.00027	0.00022	0.00032	0.00099	0.00046	-0.00056	-0.00028
$\kappa_{6,17}$	-0.00063	0.0013	0.00201	0.00047	0.00461	0.00303	-0.00285	-0.001
$\kappa_{6,18}$	0.00019	-0.0002	-0.00059	-0.00065	0.00066	6e-05	-0.0003	4e-05
$\kappa_{7,7}$	-0.04277	0.05721	0.11099	0.06734	0.05773	0.01659	0.01895	-0.04564
$\kappa_{7,8}$	-0.00213	0.00358	0.0023	-0.00266	0.02352	0.01654	-0.01558	-0.00791
$\kappa_{7,9}$	-0.00126	0.00111	0.00298	0.00207	0.0015	0.00028	-0.00024	-0.00246
$\kappa_{7,10}$	0.00308	-0.00257	-0.0092	-0.00943	0.00688	0.00247	-0.00841	0.00982
$\kappa_{7,11}$	-0.00041	0.00025	-0.00083	-6e-05	0.0015	0.00053	-0.00109	0.00056
$\kappa_{7,12}$	-0.01383	0.01047	0.02653	0.03606	-0.03913	-0.01195	0.03324	-0.03446
$\kappa_{7,13}$	-0.00805	0.01132	0.01131	0.00718	0.04423	0.0139	-0.01896	-0.00245
$\kappa_{7,14}$	0.0002	-0.00014	-0.00114	-0.00068	0.0016	0.00054	-0.00048	0.00089
$\kappa_{7,15}$	-7e-05	0.00582	-0.00265	0.00193	0.01302	0.00784	-0.01417	0.00183
$\kappa_{7,16}$	2e-05	0.00039	-0.00159	0.00108	-0.00167	0.00015	0.00199	-0.00045
$\kappa_{7,17}$	-1e-05	-0.00012	8e-05	-0.00021	0.00033	0.00052	-1e-05	0.00013
$\kappa_{7,18}$	-0.00515	0.00568	0.02031	0.01422	-0.00065	0.00911	0.00073	-0.00067
$\kappa_{8,8}$	-0.00284	0.00695	0.01437	-0.00011	0.00364	0.00746	0.04493	0.03569
$\kappa_{8,9}$	-9e-05	-0.00166	0.00233	-0.0009	0.0004	0.0021	-0.00446	-0.00393
$\kappa_{8,10}$	-0.00035	-0.00083	0.0027	-8e-05	0.00076	0.00023	0.00321	-0.00218

$\kappa_{8,11}$	0.00014	-0.00019	-0.00051	-0.00021	0.00039	0.00022	-8e-05	0.00041
$\kappa_{8,12}$	0.00577	-0.00571	-0.00442	-0.01608	-0.00505	-0.00153	-0.00625	0.01192
$\kappa_{8,13}$	-0.00461	0.00697	0.01414	0.00254	0.02403	0.01411	-0.01389	-0.00392
$\kappa_{8,14}$	-7e-05	-0.00025	0.00028	-2e-05	0.00031	0.00041	-0.00045	-0.00038
$\kappa_{8,15}$	-0.00109	8e-05	0.00224	0.00136	0.00264	0.00199	-0.00255	-0.00274
$\kappa_{8,16}$	0.00029	-0.00141	0.00103	-0.00222	0.00128	0.00131	-0.00317	-0.00022
$\kappa_{8,17}$	1e-05	-0.00064	0.00043	-0.00076	-0.00036	0.00015	0.00021	0.00039
$\kappa_{8,18}$	-0.00399	0.00683	0.00615	0.00126	0.04878	0.03176	-0.02844	-0.01246
$\kappa_{9,9}$	-0.00707	-0.03403	0.03625	0.00999	0.02353	0.02491	0.03095	0.0289
$\kappa_{9,10}$	0.00082	-0.00104	0.00143	0.00062	-0.00809	-0.0033	0.00383	0.00061
$\kappa_{9,11}$	-0.00031	0.00089	-0.00043	0.00065	0.00079	-7e-05	-0.00164	-0.00041
$\kappa_{9,12}$	-0.00025	-0.00048	-0.00096	0.00192	-0.00277	-0.00212	0.00304	-8e-05
$\kappa_{9,13}$	-0.00044	0.00097	0.00149	-0.00065	0.00171	0.00146	-0.00185	-0.00196
$\kappa_{9,14}$	9e-05	0.00138	-0.00286	0.00214	-0.00313	-0.00233	0.00269	0.0004
$\kappa_{9,15}$	-0.00123	0.00223	0.00395	0.0005	0.00907	0.0055	-0.00639	-0.00156
$\kappa_{9,16}$	0.00206	-0.00587	-0.00281	-0.00334	-0.02604	-0.01025	0.02661	-0.01503
$\kappa_{9,17}$	-2e-05	-0.00149	0.00226	0.0007	-0.00013	0.00085	-0.0052	-0.00205
$\kappa_{9,18}$	-9e-05	0.00063	0.00119	-0.00119	0.00295	0.00344	-0.00274	-0.0012
$\kappa_{10,10}$	-0.00311	-0.04602	0.00494	-0.00404	0.0048	0.00333	0.04512	0.03747
$\kappa_{10,11}$	9e-05	0.00072	0.00253	-0.00176	0.00182	0.00072	-0.00073	-0.00023
$\kappa_{10,12}$	-0.00074	0.00081	0.00295	0.00425	-0.00276	-0.00079	0.0012	-0.00124
$\kappa_{10,13}$	0.00044	-0.00038	-0.00104	-0.00259	0.00209	0.00026	-0.00188	0.00291
$\kappa_{10,14}$	-0.00023	0.00055	-0.0001	-0.00052	0.00128	0.0006	0.00065	-0.00064
$\kappa_{10,15}$	0.00139	-0.00435	-0.00478	6e-05	-0.01782	-0.00712	0.0188	-0.0136
$\kappa_{10,16}$	0.00021	-0.0002	-0.00061	-7e-05	-0.00469	-0.00119	0.00408	-0.00084
$\kappa_{10,17}$	-0.00055	-0.00015	0.00408	-0.00215	0.001	0.00103	0.00344	-0.00135

$\kappa_{10,18}$	-0.00192	0.00212	0.00761	0.0049	-0.00752	-0.00402	0.004	0.00097
$\kappa_{11,11}$	0.00044	-0.05103	0.01146	0.00433	-0.00731	-0.0099	0.02326	0.01276
$\kappa_{11,12}$	-0.0	-0.00079	-0.00083	-0.00018	-0.00103	-0.00115	0.00233	0.00089
$\kappa_{11,13}$	0.00065	-0.00046	-0.00153	-0.00043	-0.00025	-0.00092	0.00021	0.00155
$\kappa_{11,14}$	-0.00062	-0.00112	0.00503	-0.00133	0.00228	0.00268	0.00059	-0.00151
$\kappa_{11,15}$	0.00113	-0.00262	-0.00097	-0.00497	-0.00047	4e-05	0.00484	0.00443
$\kappa_{11,16}$	-0.0009	-9e-05	-0.00055	0.00432	0.00012	-0.00145	0.00442	-3e-05
$\kappa_{11,17}$	2e-05	-0.00028	9e-05	-0.00033	-0.00033	-0.00038	0.00037	0.0005
$\kappa_{11,18}$	-2e-05	0.0006	0.00026	0.00055	-0.00011	0.00052	-0.00045	0.0001
$\kappa_{12,12}$	-0.00224	0.01288	0.01881	-0.00877	0.04656	0.05813	-0.0231	0.03678
$\kappa_{12,13}$	-0.00115	0.00178	0.00963	0.00774	-0.00053	0.00433	-0.00191	-0.00088
$\kappa_{12,14}$	-0.00044	0.00041	0.00112	0.00108	-0.00021	0.00075	0.0001	-0.00019
$\kappa_{12,15}$	-0.00275	0.00123	0.00649	0.00635	-0.00156	-0.00029	0.00338	-0.00801
$\kappa_{12,16}$	-0.00042	0.00154	0.00251	-0.00225	0.00624	0.0038	-0.00453	-0.00192
$\kappa_{12,17}$	0.00011	0.00023	-0.00051	-2e-05	-8e-05	-3e-05	-0.00032	-3e-05
$\kappa_{12,18}$	0.00728	-0.01034	-0.0143	-0.02215	-0.01692	-0.00907	-0.00028	0.02429
$\kappa_{13,13}$	-0.00429	0.00903	0.00274	-0.00598	0.05437	0.07491	0.00606	-0.02414
$\kappa_{13,14}$	0.00033	-0.00011	-0.00168	-0.00017	0.00129	0.00032	-0.00026	0.00046
$\kappa_{13,15}$	-0.00229	0.00377	0.00352	0.00283	0.00904	0.0045	-0.00312	-0.00376
$\kappa_{13,16}$	0.00022	0.00014	-0.00043	-0.00071	-0.00029	-0.00015	0.00011	0.00095
$\kappa_{13,17}$	-0.00013	-0.00012	0.00039	1e-05	0.00017	0.00018	0.00018	4e-05
$\kappa_{13,18}$	-0.0042	0.00943	0.01967	0.00172	0.01145	0.0007	-0.00934	0.00152
$\kappa_{14,14}$	-0.00831	-0.01954	0.033	0.00882	0.04964	0.05638	-0.01249	-0.00534
$\kappa_{14,15}$	0.00094	0.0005	-0.00251	-0.0021	-0.00374	-0.00245	0.00281	0.00104
$\kappa_{14,16}$	-0.0003	-0.00197	0.00089	7e-05	-0.00026	-0.00091	0.00066	-0.00173
$\kappa_{14,17}$	0.00426	-0.0074	-0.00909	-0.00428	-0.02678	-0.01631	0.01369	0.00745

$\kappa_{14,18}$	-0.00033	3e-05	0.00167	0.00106	-0.00084	-0.00052	0.00112	9e-05
$\kappa_{15,15}$	-0.01183	-0.03711	0.03009	0.00446	0.0293	0.01916	0.05683	-0.03239
$\kappa_{15,16}$	0.00056	0.00058	-0.00133	-0.00092	-0.00137	-0.00079	-0.00025	0.00413
$\kappa_{15,17}$	0.00027	0.00014	-0.00047	-5e-05	8e-05	0.0004	-0.00183	0.00036
$\kappa_{15,18}$	-0.00169	0.00191	0.00641	0.00499	-0.00525	-0.00119	0.00227	-0.00027
$\kappa_{16,16}$	0.00164	-0.04167	0.01917	-0.00573	0.01331	0.01581	0.00675	0.09224
$\kappa_{16,17}$	-0.00021	-0.00041	0.00134	0.00015	0.0009	0.00087	-0.00205	-0.00121
$\kappa_{16,18}$	-0.00091	0.0011	0.00448	0.00104	0.00059	-9e-05	-0.00079	0.00067
$\kappa_{17,17}$	-0.00534	-0.02082	0.02291	0.00559	0.01722	0.01525	0.04029	0.03126
$\kappa_{17,18}$	9e-05	0.00034	-0.00052	7e-05	-0.0005	-0.00018	-0.00013	6e-05
$\kappa_{18,18}$	-0.00286	0.01103	0.0061	-0.0025	0.04319	0.04761	0.00441	0.00708

mode	v056	v057	v058	v067	v069	v070	v074	v087
$\kappa_{0,0}$	0.0	0.0	0.0	0.0	0.0	0.0	0.0	-0.0
$\kappa_{0,1}$	-0.0001	0.00188	0.00115	-0.00664	-0.0005	-0.00785	-0.0016	0.00041
$\kappa_{0,2}$	0.00419	-0.00167	-0.00013	0.00273	-0.00418	0.00288	0.00155	0.00267
$\kappa_{0,3}$	0.00282	-0.0046	0.00148	-0.00434	-0.0018	0.00194	-0.00344	0.00256
$\kappa_{0,4}$	-7e-05	0.00011	-2e-05	3e-05	8e-05	-0.00021	8e-05	2e-05
$\kappa_{0,5}$	0.00422	-0.00906	-0.00493	0.02995	-0.00191	0.00205	0.01143	0.00567
$\kappa_{0,6}$	-2e-05	-0.00063	-0.0005	-0.00022	3e-05	4e-05	-0.00067	-0.00011
$\kappa_{0,7}$	0.01385	-0.00763	-0.00561	-0.01267	0.00026	0.02143	-0.00677	0.00369
$\kappa_{0,8}$	-0.00198	-0.00972	-0.00367	0.00925	0.00095	0.00723	0.00167	-0.00092
$\kappa_{0,9}$	-0.00109	0.0005	-0.00047	-0.00027	0.00112	0.00083	0.00039	-0.00022
$\kappa_{0,10}$	-0.00044	-0.00035	0.00037	-0.00126	-0.00053	-0.0001	-0.00032	-0.00025
$\kappa_{0,11}$	0.0015	0.00072	7e-05	-0.0026	-0.00038	-0.00075	-0.0018	-0.00129
$\kappa_{0,12}$	0.01205	-0.02772	0.00032	-0.01264	0.00361	0.04447	-0.00565	-0.00281
$\kappa_{0,13}$	-0.00234	-0.01603	0.00224	-0.01734	-0.00392	0.02126	-0.00209	0.00579

$\kappa_{0,14}$	8e-05	0.00122	-4e-05	-0.00123	1e-05	-0.00078	-0.00029	-7e-05
$\kappa_{0,15}$	0.00029	-0.00451	0.00034	0.00324	-0.00222	0.00664	-0.00109	0.00041
$\kappa_{0,16}$	0.00349	-0.00076	0.00362	-0.00279	-0.00241	-0.00117	0.00059	0.00079
$\kappa_{0,17}$	-0.00029	-2e-05	-6e-05	5e-05	0.00024	0.00017	0.0002	-0.00046
$\kappa_{0,18}$	-0.00367	0.01409	0.00069	-0.01398	-0.00483	-0.00897	-0.00288	0.00126
$\kappa_{1,1}$	0.05125	0.00615	0.0078	-0.00548	-0.02655	-0.01554	-0.03378	0.02193
$\kappa_{1,2}$	-0.00042	-0.005	-0.00352	-0.00135	-0.00116	-0.00621	-0.01181	-0.00036
$\kappa_{1,3}$	-0.00523	-0.00308	-0.00205	-0.00104	0.00167	0.00025	-0.003	-0.0016
$\kappa_{1,4}$	-0.00053	0.0001	0.0001	-0.00039	0.00026	-0.00015	-0.00047	-0.00011
$\kappa_{1,5}$	0.00065	-0.00104	-0.00075	-8e-05	0.00015	0.00027	-0.00135	0.00039
$\kappa_{1,6}$	-1e-05	-0.00691	-0.00272	-0.0023	-0.0013	0.00119	-0.00386	-0.00144
$\kappa_{1,7}$	-0.00144	-0.00875	-0.00414	-0.00161	-0.00023	0.00141	-0.00589	-0.00069
$\kappa_{1,8}$	0.00023	0.00116	0.00041	-0.0002	-0.00049	-0.00074	0.00083	0.00036
$\kappa_{1,9}$	-0.00043	0.0019	-0.00056	0.00124	-0.00143	-0.00233	0.00067	0.00069
$\kappa_{1,10}$	-0.00275	0.00346	-0.00054	0.00082	9e-05	-0.01083	-0.00359	-0.00072
$\kappa_{1,11}$	0.00017	0.02206	0.00791	-0.00068	0.0008	-0.02123	0.00298	0.00106
$\kappa_{1,12}$	-0.00043	-0.0016	1e-05	0.0014	-0.00053	0.0019	-0.00037	-0.00051
$\kappa_{1,13}$	-0.00199	-0.00462	-0.0017	0.00089	0.00382	0.00076	-0.00447	-0.00155
$\kappa_{1,14}$	-0.00198	0.00076	-0.00072	-0.00148	0.00195	-0.00195	-0.00241	-0.00137
$\kappa_{1,15}$	-0.00565	0.01509	0.00521	0.01282	0.00858	-0.02343	0.00479	-0.00013
$\kappa_{1,16}$	0.00106	-0.00261	0.00201	-0.00444	0.00097	0.00538	-0.00206	0.00075
$\kappa_{1,17}$	0.00032	-2e-05	8e-05	0.00012	-5e-05	1e-05	9e-05	0.00012
$\kappa_{1,18}$	-3e-05	-0.00062	-0.00071	0.00142	0.00045	0.00051	0.00022	-0.00065
$\kappa_{2,2}$	0.03708	0.01803	-0.00303	-0.00268	-0.02461	0.00989	0.01311	0.02198
$\kappa_{2,3}$	0.00096	-7e-05	-0.00095	0.00071	-0.00048	0.0008	-0.00043	0.00023
$\kappa_{2,4}$	0.00211	-0.00088	-0.00035	0.00177	-0.00037	0.00053	0.00238	0.00016

$\kappa_{2,5}$	0.00063	0.00119	0.0015	0.00052	-0.00311	0.00132	0.00189	0.00082
$\kappa_{2,6}$	-0.00025	-0.0018	-0.00065	-0.00046	-0.00042	4e-05	-0.00122	-0.00025
$\kappa_{2,7}$	0.0004	3e-05	0.00037	0.00054	-0.00052	0.00133	0.00043	-0.00032
$\kappa_{2,8}$	4e-05	0.00107	0.00018	-3e-05	-0.00015	-0.00053	0.0008	0.00019
$\kappa_{2,9}$	0.00414	-0.00685	0.00452	-0.00304	0.00688	0.00996	0.00047	-0.00137
$\kappa_{2,10}$	-0.00077	0.00095	-0.00062	0.00043	0.00055	-0.00165	0.00014	-0.00086
$\kappa_{2,11}$	-0.00953	0.02943	-0.004	-0.00547	-0.00191	-0.03333	0.00942	-0.0016
$\kappa_{2,12}$	1e-05	0.00146	-0.00183	0.00137	-0.00049	-0.0007	0.00172	0.00095
$\kappa_{2,13}$	0.00017	0.00014	0.00012	0.00123	0.0001	0.00037	0.00051	2e-05
$\kappa_{2,14}$	0.00097	-0.00091	0.00021	0.00063	-0.00068	9e-05	0.00073	0.00021
$\kappa_{2,15}$	-0.00161	0.00429	-6e-05	0.00219	0.00219	-0.0044	0.00117	-0.00211
$\kappa_{2,16}$	-0.00135	0.0151	-0.00239	0.01412	-0.00633	-0.02187	0.00805	0.00471
$\kappa_{2,17}$	4e-05	0.00033	0.00029	0.00048	0.00011	-0.00011	0.00071	-0.00016
$\kappa_{2,18}$	0.00042	0.00026	-0.00039	0.00064	-6e-05	-0.00104	7e-05	0.00043
$\kappa_{3,3}$	0.00069	0.00244	0.00397	-0.00339	-0.00945	-0.00328	-0.00412	0.00053
$\kappa_{3,4}$	0.00013	-7e-05	-0.0001	-4e-05	0.00011	6e-05	-2e-05	1e-05
$\kappa_{3,5}$	0.00092	-0.00333	-0.0007	0.00387	0.00099	0.00283	-3e-05	-0.0009
$\kappa_{3,6}$	0.00025	0.00104	0.00036	-4e-05	0.00043	-0.00022	0.00053	-0.0001
$\kappa_{3,7}$	-0.0013	3e-05	-0.00064	-0.0023	-0.0008	-0.0016	-0.00104	-7e-05
$\kappa_{3,8}$	1e-05	-0.00108	-0.00045	0.00087	0.00039	0.00113	4e-05	-0.00018
$\kappa_{3,9}$	0.00014	0.00043	0.00015	0.00021	-0.00038	-0.00022	0.00016	0.00033
$\kappa_{3,10}$	4e-05	-0.00129	-0.00036	8e-05	-0.00017	0.00056	-0.00068	6e-05
$\kappa_{3,11}$	-2e-05	-0.0029	-0.00384	0.00104	0.00027	0.00601	-0.00074	-0.00095
$\kappa_{3,12}$	0.00031	-0.00201	-0.0003	0.00038	-0.0001	0.00341	0.00054	-0.00094
$\kappa_{3,13}$	0.00025	0.00305	0.00125	-0.00387	-0.00151	-0.00278	-0.00104	-0.00037
$\kappa_{3,14}$	-0.00041	0.0006	0.00012	-0.0	0.00012	0.00116	0.00163	5e-05

$\kappa_{3,15}$	0.00034	-0.00195	-0.00065	-0.00063	-0.0009	-5e-05	-0.00129	-1e-05
$\kappa_{3,16}$	-0.00016	0.00035	-0.00011	-0.00088	0.00011	0.00021	-0.0004	-0.00023
$\kappa_{3,17}$	-4e-05	-8e-05	-6e-05	-8e-05	4e-05	6e-05	-2e-05	-3e-05
$\kappa_{3,18}$	-0.00066	0.00142	-1e-05	-0.00145	0.00045	-0.00014	0.00017	-0.0007
$\kappa_{4,4}$	0.02991	0.00857	0.01399	0.01962	-0.05088	-0.00076	0.03165	0.04958
$\kappa_{4,5}$	-7e-05	0.00042	0.00018	-0.00042	-0.00012	-0.00048	0.00013	-4e-05
$\kappa_{4,6}$	-0.00058	-0.0009	-0.0011	0.0002	-0.00025	-6e-05	-0.00016	-0.00023
$\kappa_{4,7}$	-0.00017	0.00038	-5e-05	0.00014	-5e-05	-0.00044	0.00021	8e-05
$\kappa_{4,8}$	-8e-05	-0.00021	-0.00034	-0.00012	0.00021	-8e-05	-0.00031	-0.0001
$\kappa_{4,9}$	0.005	0.00449	0.00426	-0.00096	-0.00037	-0.00259	0.0033	-0.0004
$\kappa_{4,10}$	0.00091	0.00046	0.00067	-0.00029	-9e-05	6e-05	0.00033	9e-05
$\kappa_{4,11}$	0.00059	-0.00077	0.00053	-3e-05	0.00023	0.0	0.00025	-0.0002
$\kappa_{4,12}$	9e-05	-3e-05	6e-05	-3e-05	2e-05	0.00018	-4e-05	-0.00012
$\kappa_{4,13}$	-0.00026	-0.00016	0.0	-3e-05	0.00014	-4e-05	-4e-05	-1e-05
$\kappa_{4,14}$	0.00825	-0.01587	0.00636	-8e-05	0.00419	0.01513	-0.00482	-0.00311
$\kappa_{4,15}$	5e-05	-0.00028	-3e-05	-0.00011	-3e-05	0.00019	-0.00043	0.00018
$\kappa_{4,16}$	0.00129	0.0039	-0.00065	0.00189	0.00015	0.00446	0.00413	0.00215
$\kappa_{4,17}$	-0.00092	-0.00072	-0.00174	-0.00039	-0.00072	-0.00029	-0.00044	-0.00034
$\kappa_{4,18}$	5e-05	-0.00033	-0.00011	0.00018	5e-05	-3e-05	3e-05	-0.0001
$\kappa_{5,5}$	0.03571	-0.00736	0.02363	0.03527	-0.05386	0.01261	0.01856	0.03981
$\kappa_{5,6}$	-0.00024	-0.00046	-0.00018	0.0002	0.0001	0.00033	-5e-05	1e-05
$\kappa_{5,7}$	0.00183	0.01647	0.00708	-0.01341	-0.01895	-0.02731	0.00069	-0.00072
$\kappa_{5,8}$	-0.00291	0.0036	-0.00491	0.00178	-3e-05	-0.00271	0.00061	0.00091
$\kappa_{5,9}$	0.00117	0.00023	0.00099	-0.00203	-0.00033	-0.00241	-0.00179	0.00041
$\kappa_{5,10}$	0.00091	-0.0048	-6e-05	0.0068	-0.00113	0.00515	0.00221	-0.00096
$\kappa_{5,11}$	0.00049	-0.00182	-0.00015	0.00124	-3e-05	0.0016	-0.00055	0.00048

$\kappa_{5,12}$	-0.01861	0.01976	-0.01753	-0.03434	0.02574	-0.01374	0.00296	-0.01193
$\kappa_{5,13}$	0.00047	0.01749	0.00447	-0.00961	-0.00921	-0.01819	0.00779	-0.00104
$\kappa_{5,14}$	-5e-05	6e-05	0.00012	0.00071	9e-05	8e-05	0.00037	0.00033
$\kappa_{5,15}$	0.00183	0.00482	0.00128	-0.00243	-0.00262	-0.00529	0.00077	0.00082
$\kappa_{5,16}$	0.00074	-0.00203	-0.00226	0.00463	-0.00051	0.00458	0.00251	0.00185
$\kappa_{5,17}$	-0.0001	-9e-05	-0.0001	0.00024	8e-05	-8e-05	2e-05	-4e-05
$\kappa_{5,18}$	-0.0013	-0.00625	-0.0018	0.01596	0.00372	-0.01078	0.0007	-0.00491
$\kappa_{6,6}$	0.03929	0.00101	0.0146	-0.02416	-0.05071	-0.00525	-0.03477	0.04923
$\kappa_{6,7}$	0.00063	0.00187	0.00068	-0.00195	-0.00021	-0.00232	0.00077	-2e-05
$\kappa_{6,8}$	-0.00022	-0.00038	-0.00022	0.00037	0.0001	0.00031	-3e-05	-1e-05
$\kappa_{6,9}$	-0.00027	0.00083	0.00011	4e-05	8e-05	-0.00037	0.00035	9e-05
$\kappa_{6,10}$	-0.0011	-0.00419	-0.00176	-0.00063	-0.00026	-0.00097	-0.00351	-0.00097
$\kappa_{6,11}$	0.0001	0.00282	0.00091	0.00021	0.00038	-0.00195	0.00067	0.00039
$\kappa_{6,12}$	-0.0001	-0.00023	-4e-05	0.0003	0.0001	0.00027	0.00012	-0.00016
$\kappa_{6,13}$	8e-05	0.00027	0.00021	-0.0004	0.00016	8e-05	-0.0001	-0.00013
$\kappa_{6,14}$	-0.00495	-0.01587	-0.0082	-0.00141	-0.00402	0.01907	-0.00081	0.00255
$\kappa_{6,15}$	-0.00689	-0.00581	-0.00653	0.0012	0.0001	0.00305	-0.00415	0.00092
$\kappa_{6,16}$	0.00035	-0.00015	0.00037	-0.00012	0.00018	4e-05	3e-05	-0.00035
$\kappa_{6,17}$	0.00114	0.00015	0.00174	0.00022	0.00074	0.0002	0.00015	8e-05
$\kappa_{6,18}$	0.00016	-0.00076	-3e-05	0.00097	1e-05	0.0004	4e-05	-4e-05
$\kappa_{7,7}$	0.04386	0.09119	0.04932	-0.09776	-0.08015	-0.05834	-0.01427	0.03798
$\kappa_{7,8}$	0.00728	-0.0086	0.00721	0.0031	0.00588	0.00742	-0.00609	-0.00387
$\kappa_{7,9}$	0.00099	0.003	0.00121	-0.00287	-0.00061	-0.00216	-2e-05	0.00073
$\kappa_{7,10}$	0.00078	-0.01017	-0.00125	0.01323	0.00025	0.01411	0.00163	-0.00063
$\kappa_{7,11}$	0.0004	-0.00133	0.00032	0.001	-0.00033	0.00164	-0.00077	0.00034
$\kappa_{7,12}$	-0.00925	0.04101	-0.00094	-0.05682	0.00165	-0.04623	-0.01163	0.00344

$\kappa_{7,13}$	0.01516	0.01347	0.01436	-0.00318	-0.01351	-0.01003	0.00194	0.0018
$\kappa_{7,14}$	0.00081	-0.00118	0.00037	0.00187	-0.00085	0.00121	-0.00025	0.00079
$\kappa_{7,15}$	0.00121	0.00215	0.0056	0.00851	-0.00012	0.00927	0.00899	-0.00223
$\kappa_{7,16}$	-0.00192	0.00111	-0.00054	-0.00061	0.00181	0.00064	0.00036	-0.001
$\kappa_{7,17}$	0.0001	-0.00037	-1e-05	8e-05	0.0001	0.00034	-0.00032	0.00015
$\kappa_{7,18}$	-0.00066	0.01658	0.00035	-0.03075	-0.00038	0.00129	-0.00208	0.00523
$\kappa_{8,8}$	0.06084	0.00694	0.02125	-0.00509	-0.09139	-0.0004	0.00018	0.09941
$\kappa_{8,9}$	0.00131	0.00511	0.00065	0.00175	0.00256	-0.00119	0.00315	2e-05
$\kappa_{8,10}$	-0.00252	0.00502	-0.00066	-0.00252	0.00125	-0.00152	-0.00048	-5e-05
$\kappa_{8,11}$	0.00021	-0.00054	0.00017	0.0007	-5e-05	0.00071	0.00023	-2e-05
$\kappa_{8,12}$	-0.00215	-0.02278	-0.00551	0.00572	0.00437	0.02336	-0.00116	-0.00551
$\kappa_{8,13}$	0.00648	0.001	0.00948	-0.00727	-0.00126	0.00398	-0.00236	0.00497
$\kappa_{8,14}$	0.00014	-8e-05	6e-05	-0.0001	0.00034	1e-05	-0.00015	-0.00024
$\kappa_{8,15}$	0.0015	8e-05	0.00056	-0.00244	0.00087	-0.00097	-0.00266	-0.00091
$\kappa_{8,16}$	0.00087	0.0006	0.00031	0.00117	0.00094	0.00068	0.0012	0.0004
$\kappa_{8,17}$	-0.00041	-0.00052	-0.00057	1e-05	0.00018	-7e-05	-0.00065	7e-05
$\kappa_{8,18}$	0.01201	-0.00318	0.0116	0.01303	0.01056	0.00195	0.0009	-0.00969
$\kappa_{9,9}$	0.07717	0.01517	0.018	0.01491	-0.06332	-0.01404	0.00056	0.06892
$\kappa_{9,10}$	-0.00098	-0.00199	-0.00091	0.00641	-0.00291	-0.00239	0.00294	-0.0001
$\kappa_{9,11}$	-0.00151	0.00154	0.00017	-0.0003	0.0014	-0.00402	0.00084	-0.00182
$\kappa_{9,12}$	-0.00059	0.00343	-0.00047	-0.00135	-0.00015	-0.00097	0.00128	0.00022
$\kappa_{9,13}$	0.00039	-0.0001	0.00073	-0.00054	0.00019	-0.00247	-0.00112	-0.00102
$\kappa_{9,14}$	-0.00069	0.00337	0.00035	0.00019	-0.00142	-0.00232	0.00227	0.00181
$\kappa_{9,15}$	0.00212	0.00037	0.00291	-0.00111	0.00185	0.00298	0.00031	-0.00038
$\kappa_{9,16}$	-0.00047	-0.0109	-0.00487	-0.0026	-0.00187	-0.00516	-0.01665	0.0005
$\kappa_{9,17}$	0.00074	0.00479	0.00208	0.00129	0.00048	0.0002	0.00321	0.00129

$\kappa_{9,18}$	0.00056	-0.00242	0.00032	0.0008	0.00069	-0.00031	-0.00121	-0.00031
$\kappa_{10,10}$	0.07	0.01321	0.00405	-0.02014	-0.06257	0.00753	-0.02083	0.06876
$\kappa_{10,11}$	0.00026	-0.00219	-0.00023	-0.00021	0.00035	0.0017	-0.00073	0.00108
$\kappa_{10,12}$	0.00114	0.00324	0.00094	-0.00454	-7e-05	-0.00166	0.0006	0.00105
$\kappa_{10,13}$	-0.00038	-0.00153	-0.00054	0.00212	-0.0004	0.00192	-0.00041	0.00041
$\kappa_{10,14}$	0.00045	2e-05	0.00014	0.00056	-0.00032	-2e-05	-0.00086	-5e-05
$\kappa_{10,15}$	-0.00036	-0.00525	-0.00414	0.00106	-0.00192	-0.00976	-0.01388	0.00145
$\kappa_{10,16}$	-0.00081	-0.00079	-0.00056	0.00042	-0.00058	-0.00176	-0.00095	0.00012
$\kappa_{10,17}$	-0.00124	0.00059	0.00069	-0.00156	0.00092	0.00117	-0.00123	5e-05
$\kappa_{10,18}$	-0.00377	0.00914	-0.00234	-0.01246	0.00022	-0.00379	-0.002	-0.00026
$\kappa_{11,11}$	0.011	0.00772	-0.01246	-0.00604	-0.0099	-0.00788	-0.00888	0.02292
$\kappa_{11,12}$	-0.00037	0.0004	-2e-05	0.00051	-0.00067	-0.00037	0.00017	0.00045
$\kappa_{11,13}$	5e-05	-0.00153	-0.00026	0.00098	-0.00022	0.00192	0.00044	0.00023
$\kappa_{11,14}$	-0.00408	-0.00032	-0.0002	-0.00175	0.00425	0.00015	-0.00252	-0.00197
$\kappa_{11,15}$	0.00024	-0.00564	-0.00262	-0.00035	-0.00133	-0.00094	-0.00168	0.00319
$\kappa_{11,16}$	-0.00023	0.00456	-0.00127	-0.00068	-0.00204	-0.00111	0.0007	0.0047
$\kappa_{11,17}$	-0.00059	-0.00096	-0.00041	5e-05	0.00035	0.00055	-0.00032	-0.00032
$\kappa_{11,18}$	0.00021	0.00103	0.00064	-0.00083	-0.00015	6e-05	0.00062	-3e-05
$\kappa_{12,12}$	0.03863	-0.00909	0.01921	0.01869	-0.04307	0.03436	0.0176	0.03088
$\kappa_{12,13}$	-0.00251	0.00858	-0.00169	-0.01641	0.00398	-0.00182	0.00182	-0.00138
$\kappa_{12,14}$	4e-05	0.00059	0.00018	-0.00144	2e-05	-0.00043	-7e-05	0.00073
$\kappa_{12,15}$	0.00081	0.00656	0.00152	-0.00804	-0.00043	-0.0073	-0.00147	0.00108
$\kappa_{12,16}$	0.0016	-0.00419	0.00151	0.0013	-0.00075	0.00151	-0.00251	0.0006
$\kappa_{12,17}$	-0.00024	-0.00025	0.00013	0.00024	0.00012	0.00047	0.00024	-0.00031
$\kappa_{12,18}$	-0.00432	-0.02514	-0.00983	0.00842	-0.00198	0.03313	0.00437	0.00175
$\kappa_{13,13}$	0.05193	-0.01473	0.0251	0.01855	-0.02513	-0.01354	-0.02695	0.0247

$\kappa_{13,14}$	0.00047	-0.0006	0.00017	0.00185	2e-05	0.00106	0.00064	6e-05
$\kappa_{13,15}$	0.00099	0.0059	0.00354	-0.00278	-0.00046	-0.00744	0.00165	0.0004
$\kappa_{13,16}$	0.00024	-0.00025	0.00011	0.00048	-0.00039	0.00041	0.0002	0.00058
$\kappa_{13,17}$	0.00018	-5e-05	-0.0	-0.00016	-0.00013	-0.00012	-0.00022	0.00037
$\kappa_{13,18}$	0.00321	0.00251	0.00528	-0.01413	0.00057	0.00881	0.001	-0.00308
$\kappa_{14,14}$	0.03147	0.00505	0.01666	-0.00086	-0.02076	-0.00961	-0.0084	0.00905
$\kappa_{14,15}$	-0.00084	-0.00318	-0.00033	-0.00107	-0.00094	0.00418	-0.00134	0.00172
$\kappa_{14,16}$	-0.0003	0.00054	-0.00064	-0.00088	0.00123	-0.00144	-0.00168	-0.00104
$\kappa_{14,17}$	-0.00903	-0.00376	-0.01013	0.0	-0.00011	0.00119	0.00069	-0.00198
$\kappa_{14,18}$	-0.00057	0.00136	-0.00022	-0.00248	0.00017	-0.00095	-0.00037	-0.00014
$\kappa_{15,15}$	0.08826	0.00159	0.01799	-0.02103	-0.06214	-0.01333	-0.06268	0.06955
$\kappa_{15,16}$	-0.00141	-0.00028	-0.00064	-0.0001	0.00026	0.00261	0.00218	0.00021
$\kappa_{15,17}$	0.00072	-0.00062	-6e-05	0.00043	-0.00025	0.00059	0.00021	0.00021
$\kappa_{15,18}$	-0.00157	0.00709	-0.0009	-0.0116	0.00056	-0.00185	-0.00152	-0.00027
$\kappa_{16,16}$	0.06707	0.01508	0.00487	0.0216	-0.06371	0.02319	0.03979	0.07044
$\kappa_{16,17}$	0.00064	0.00182	0.00093	-0.00015	0.00016	-0.00049	0.00056	0.00043
$\kappa_{16,18}$	5e-05	0.0015	5e-05	-0.00482	-0.00065	0.00011	-0.0014	0.00066
$\kappa_{17,17}$	0.06776	0.0071	0.0254	-0.00091	-0.09146	-0.00269	-0.00283	0.09826
$\kappa_{17,18}$	-0.00042	-3e-05	3e-05	-9e-05	0.00032	0.00055	0.00035	-0.00053
$\kappa_{18,18}$	0.07166	-6e-05	0.01605	0.0237	-0.04157	-0.00635	0.0019	0.00997

mode	v105	v106	v108	v109	v110	v122	v123	v124
$\kappa_{0,0}$	0.0	0.0	0.0	0.0	0.0	0.0	0.0	0.0
$\kappa_{0,1}$	0.00017	0.01229	0.01713	-0.01598	-0.01214	0.00612	-0.00962	-0.01122
$\kappa_{0,2}$	-0.00741	-0.01246	0.00259	0.01045	-0.00911	0.00121	0.00399	0.00536
$\kappa_{0,3}$	0.00171	-0.00118	-0.0083	-0.01575	0.00679	-0.00016	0.00206	-0.00414
$\kappa_{0,4}$	8e-05	-3e-05	-2e-05	0.0002	2e-05	-0.00011	4e-05	-0.00011

$\kappa_{0,5}$	-0.07106	-0.18032	0.14186	0.27715	-0.12923	0.04343	0.05993	0.10645
$\kappa_{0,6}$	-0.0	0.00066	-0.00013	-0.00089	-3e-05	6e-05	1e-05	0.00039
$\kappa_{0,7}$	-0.05867	0.1097	0.13714	-0.16017	-0.12787	0.0579	-0.07281	-0.03916
$\kappa_{0,8}$	0.00988	-0.03425	-0.02093	0.04863	0.01952	-0.01164	0.02006	0.01395
$\kappa_{0,9}$	0.00153	0.00026	-0.00214	-0.00039	0.00198	-8e-05	-0.00033	-0.00041
$\kappa_{0,10}$	-0.00016	-0.00099	-0.00229	0.00192	0.00296	0.00022	-0.00107	-0.00087
$\kappa_{0,11}$	-0.00238	0.00252	0.004	-0.00628	-0.00704	0.00356	-0.00462	-0.00014
$\kappa_{0,12}$	0.02546	0.0161	-0.02673	-0.04431	0.05114	-0.04987	0.00924	-0.05845
$\kappa_{0,13}$	-0.01158	0.03191	0.01182	-0.04871	-0.0278	0.03463	-0.04565	-0.02247
$\kappa_{0,14}$	-0.00079	0.00276	-0.00059	-0.00364	-0.00034	0.00039	-0.00178	-0.00195
$\kappa_{0,15}$	-0.00077	-0.01211	-0.01138	0.01667	0.00833	-0.00152	0.00138	0.0026
$\kappa_{0,16}$	0.00173	0.00988	-0.00482	-0.01112	0.00421	-0.00273	-0.00092	-0.00521
$\kappa_{0,17}$	0.00014	-0.00026	-0.00012	0.00031	0.00054	0.00028	-0.00029	0.00042
$\kappa_{0,18}$	0.00305	0.051	0.03239	-0.0783	-0.02071	0.01324	-0.03399	-0.02568
$\kappa_{1,1}$	-0.02399	0.03919	0.12128	-0.0682	-0.14441	0.02259	-0.01122	0.01354
$\kappa_{1,2}$	0.0045	0.01336	-0.0136	-0.02721	0.00708	0.00103	0.00129	0.00844
$\kappa_{1,3}$	-0.00106	0.00128	0.00082	-0.00319	0.00084	0.00266	-0.00217	0.00127
$\kappa_{1,4}$	0.00091	-0.0003	-0.00045	0.00032	0.00089	0.00031	0.00048	0.00064
$\kappa_{1,5}$	0.00074	0.00019	-0.00151	-0.00091	0.00053	-0.00181	0.00066	-0.0009
$\kappa_{1,6}$	-0.00226	-0.00189	-0.00239	0.00345	0.00138	0.00208	-0.00048	0.00388
$\kappa_{1,7}$	-0.00234	0.00371	0.00454	-0.00469	-0.00374	-0.00032	0.00254	0.00219
$\kappa_{1,8}$	0.00016	0.00184	0.00029	-0.0024	-0.00075	0.00056	-0.00155	-0.00099
$\kappa_{1,9}$	-0.00513	-0.01142	0.01399	0.02059	-0.01256	0.00573	0.00074	0.00682
$\kappa_{1,10}$	0.01994	-0.02896	-0.05681	0.04103	0.04657	-0.01262	0.01418	0.01474
$\kappa_{1,11}$	0.00749	-0.00115	0.02259	0.01039	0.00237	-0.00107	0.01622	-0.01217
$\kappa_{1,12}$	-0.00055	-0.00332	-0.00105	0.00487	0.00118	-0.00021	0.00128	0.00268

$\kappa_{1,13}$	0.00192	-0.00661	-0.00375	0.01	0.00579	-0.00371	0.00521	0.00187
$\kappa_{1,14}$	0.00128	0.00313	0.00011	-0.00507	0.00152	0.00177	-0.00124	0.00043
$\kappa_{1,15}$	0.05606	-0.12356	-0.16338	0.18981	0.13968	-0.05881	0.07162	0.03351
$\kappa_{1,16}$	0.01522	0.03101	-0.03521	-0.05127	0.03124	-0.01346	-0.00751	-0.02127
$\kappa_{1,17}$	-0.00027	-0.00048	-0.00039	0.00062	0.00011	-0.00039	0.00012	0.0002
$\kappa_{1,18}$	-0.0018	-0.00404	0.00182	0.00553	-0.00181	0.00129	0.00094	0.00281
$\kappa_{2,2}$	-0.02171	-0.0184	0.10946	0.04269	-0.1425	0.02141	-0.03276	-0.00657
$\kappa_{2,3}$	4e-05	-0.00028	0.00062	-0.00096	-0.00047	-0.00039	-0.00079	-0.00159
$\kappa_{2,4}$	-0.00275	0.00015	0.00127	0.00026	-0.00216	-0.002	-0.00059	-0.00272
$\kappa_{2,5}$	-0.00355	-0.00543	0.00835	0.01136	-0.00757	0.00336	0.00146	0.00488
$\kappa_{2,6}$	-2e-05	-0.00104	-0.00124	0.00181	0.00123	0.00023	0.00046	0.00124
$\kappa_{2,7}$	-0.00385	0.00078	0.00861	-0.00196	-0.0075	0.0036	-0.00191	0.00151
$\kappa_{2,8}$	-0.00052	0.00089	0.00137	-0.00132	-0.00118	0.0005	-0.00045	-0.00025
$\kappa_{2,9}$	0.01466	0.03762	-0.04547	-0.06775	0.04449	-0.02498	0.00401	-0.02914
$\kappa_{2,10}$	0.00202	-0.00602	-0.00823	0.00855	0.00597	-0.0012	0.0038	0.00427
$\kappa_{2,11}$	-0.00524	0.00194	-0.01994	0.00809	-0.00567	0.00083	-0.01141	0.00816
$\kappa_{2,12}$	-0.00093	-0.00225	0.00018	0.00173	-0.00218	0.00085	0.00127	0.00204
$\kappa_{2,13}$	-0.00065	-0.00464	0.0003	0.00564	-0.00023	-0.00013	0.00182	0.00178
$\kappa_{2,14}$	0.00036	-0.0055	-0.00958	0.00823	0.0051	-0.00299	0.00329	0.00154
$\kappa_{2,15}$	0.00607	-0.02064	-0.02072	0.03335	0.01756	-0.00453	0.00821	0.00738
$\kappa_{2,16}$	-0.05664	-0.11536	0.13972	0.1971	-0.12647	0.04968	0.0298	0.08061
$\kappa_{2,17}$	-0.00021	-0.00025	0.00055	0.00067	-0.00017	-5e-05	0.00059	0.0002
$\kappa_{2,18}$	-0.00023	-0.00148	-0.00119	0.00176	-0.0005	-0.00072	0.00077	0.00045
$\kappa_{3,3}$	-0.03568	0.02105	0.08715	-0.02663	-0.08634	0.04773	-0.02994	0.00846
$\kappa_{3,4}$	-7e-05	-0.00017	0.00016	0.00029	-0.00037	0.00011	2e-05	0.00014
$\kappa_{3,5}$	-0.00287	-0.01322	0.00301	0.02124	-0.00246	-0.0001	0.00586	0.00658

$\kappa_{3,6}$	-0.00036	2e-05	0.00039	-0.00076	-0.00065	5e-05	-0.00018	0.00028
$\kappa_{3,7}$	-0.00321	0.00891	0.00865	-0.01068	-0.00839	0.00611	-0.00663	-0.00243
$\kappa_{3,8}$	0.0013	-0.00373	-0.00314	0.00499	0.00268	-0.0015	0.00194	0.00131
$\kappa_{3,9}$	-0.00041	0.00049	0.00095	-7e-05	-0.00091	0.00014	-0.00034	-0.0001
$\kappa_{3,10}$	6e-05	-0.00086	-0.00164	0.00083	0.0014	-0.00045	0.00042	0.00035
$\kappa_{3,11}$	-0.00173	0.00018	-0.0018	0.00038	-0.0015	-0.00368	-0.00274	-0.00319
$\kappa_{3,12}$	-0.00272	-0.00348	0.00438	0.00576	-0.00385	0.00106	0.00015	0.00133
$\kappa_{3,13}$	-0.0061	0.01331	0.01183	-0.01905	-0.0105	0.00611	-0.00878	-0.0057
$\kappa_{3,14}$	0.00076	-0.00032	-0.00048	-0.00055	0.00107	0.00023	-0.00087	-0.00075
$\kappa_{3,15}$	-0.00094	-0.00043	3e-05	0.00109	-0.00074	0.00057	-0.00048	0.00103
$\kappa_{3,16}$	-0.00013	0.00031	-0.00134	-0.00138	0.0005	0.00052	-0.00088	0.0003
$\kappa_{3,17}$	7e-05	-0.00013	-2e-05	8e-05	6e-05	-2e-05	-3e-05	-1e-05
$\kappa_{3,18}$	-0.00139	0.00246	0.00271	-0.00233	-0.00253	0.00195	-0.00229	-0.00015
$\kappa_{4,4}$	-0.03494	0.03809	0.05289	-0.04602	-0.0756	-0.00611	-0.00076	-0.04376
$\kappa_{4,5}$	-2e-05	0.00131	-5e-05	-0.00131	5e-05	0.00019	-0.0004	-0.00014
$\kappa_{4,6}$	0.00035	-0.00102	-0.0007	0.00178	-1e-05	0.00088	0.00018	0.00159
$\kappa_{4,7}$	0.00018	-0.0002	0.00014	0.00036	-0.00032	0.00023	-1e-05	0.00035
$\kappa_{4,8}$	-5e-05	0.00036	-0.00014	-0.00044	9e-05	-8e-05	0.00019	7e-05
$\kappa_{4,9}$	0.00061	-0.00066	-0.00194	0.00048	0.00212	-0.00287	0.00279	0.00138
$\kappa_{4,10}$	0.00016	-0.0001	-0.00065	-7e-05	0.00038	-0.00065	0.00037	-1e-05
$\kappa_{4,11}$	-0.00052	-0.00114	0.00125	0.0018	-0.00072	-0.00015	0.0009	0.00015
$\kappa_{4,12}$	-0.00018	9e-05	1e-05	-0.00031	-0.0001	-5e-05	-0.00017	-0.00014
$\kappa_{4,13}$	0.00035	-0.0001	-0.00014	0.00038	0.00034	-0.0	0.00014	7e-05
$\kappa_{4,14}$	-0.00281	-0.00891	0.00789	0.0118	0.00306	-0.00859	0.00973	-0.00567
$\kappa_{4,15}$	0.0001	-4e-05	3e-05	1e-05	-0.00014	-0.00016	0.00025	0.0001
$\kappa_{4,16}$	-0.0006	-0.00229	-0.00443	0.00249	-0.00042	-0.00081	-0.0012	0.00282

$\kappa_{4,17}$	2e-05	-0.00111	-0.0003	0.00203	-0.00119	0.00213	-0.00119	0.00236
$\kappa_{4,18}$	-0.00029	-0.00042	-0.00014	0.00073	-6e-05	1e-05	0.00027	0.0003
$\kappa_{5,5}$	-0.10097	-0.09584	0.20117	0.13455	-0.16935	0.06744	0.01947	0.04071
$\kappa_{5,6}$	0.00027	-0.00037	-0.00081	0.00061	0.0009	-0.00045	0.00034	-0.00018
$\kappa_{5,7}$	-0.07057	0.07145	0.12596	-0.09644	-0.11597	0.06823	-0.05329	-0.00204
$\kappa_{5,8}$	0.01021	-0.0082	-0.01873	0.01219	0.01163	-0.00576	0.00373	0.00402
$\kappa_{5,9}$	0.00066	0.0072	-0.00147	-0.00925	0.00193	-0.0027	-0.00095	-0.00468
$\kappa_{5,10}$	-0.00608	-0.02292	0.01046	0.03036	-0.01065	0.00699	0.00692	0.01577
$\kappa_{5,11}$	-0.00042	-0.00512	0.00127	0.00632	-0.00095	-0.00136	0.00249	0.00085
$\kappa_{5,12}$	0.0558	0.09714	-0.11615	-0.12111	0.08569	-0.0397	-0.05458	-0.05208
$\kappa_{5,13}$	-0.03778	0.04353	0.06647	-0.05626	-0.06255	0.03439	-0.03675	-0.00892
$\kappa_{5,14}$	0.00134	-0.00183	0.00138	0.00232	-0.00016	0.00046	0.00104	0.00116
$\kappa_{5,15}$	-0.00751	0.00774	0.01789	-0.01088	-0.01864	0.00858	-0.00685	0.00081
$\kappa_{5,16}$	0.00214	-0.01246	-0.01176	0.01213	0.00509	-0.00266	0.00517	0.00523
$\kappa_{5,17}$	0.00017	-0.0008	-0.00028	0.00108	0.00033	-0.00029	0.0004	0.00038
$\kappa_{5,18}$	0.00475	-0.03899	-0.0045	0.05927	0.0062	2e-05	0.0236	0.03174
$\kappa_{6,6}$	-0.03608	0.00277	0.05659	-0.00854	-0.07727	0.00215	0.02697	-0.00849
$\kappa_{6,7}$	-0.00031	0.005	0.00182	-0.00594	-0.00158	0.00094	-0.0024	-0.00197
$\kappa_{6,8}$	0.00011	-0.0011	-0.00035	0.00146	0.00039	-0.00022	0.0005	0.00035
$\kappa_{6,9}$	-0.00019	-0.00024	-5e-05	0.0004	-0.0004	0.0003	6e-05	0.0009
$\kappa_{6,10}$	0.00101	0.00244	0.00012	-0.00308	0.00167	-0.0005	-0.00045	-0.00421
$\kappa_{6,11}$	5e-05	0.00148	0.00197	-0.00236	-0.00136	3e-05	1e-05	-0.00051
$\kappa_{6,12}$	0.00011	-0.00109	-0.00038	0.00162	0.00045	-4e-05	0.00039	0.00031
$\kappa_{6,13}$	-7e-05	0.00121	0.00042	-0.00178	-0.00041	0.00024	-0.00079	-0.00035
$\kappa_{6,14}$	0.00476	0.00453	-0.00511	-0.01179	-0.00206	0.00846	-0.01811	-0.00737
$\kappa_{6,15}$	9e-05	0.0028	0.0031	-0.00258	-0.00391	0.00316	-0.00403	-0.00564

$\kappa_{6,16}$	-0.0002	0.00022	0.00084	-0.00045	-0.00047	0.00018	-0.00024	0.00011
$\kappa_{6,17}$	-0.00038	0.00112	0.00073	-0.00198	0.0006	-0.00169	0.00092	-0.00157
$\kappa_{6,18}$	-0.00033	-0.00323	-0.0002	0.00429	7e-05	-8e-05	0.00169	0.00181
$\kappa_{7,7}$	-0.18336	0.32535	0.43858	-0.40763	-0.34469	0.16203	-0.1651	-0.12532
$\kappa_{7,8}$	-0.00203	-0.02432	-0.01153	0.03358	0.01577	-0.01396	0.02028	0.00701
$\kappa_{7,9}$	-0.00292	0.00741	0.00501	-0.01042	-0.0038	0.00111	-0.00199	-0.00336
$\kappa_{7,10}$	-0.00217	-0.04663	-0.00885	0.06324	0.0062	-0.00498	0.02065	0.01759
$\kappa_{7,11}$	0.00311	-0.00741	-0.00776	0.00983	0.00762	-0.00414	0.00559	6e-05
$\kappa_{7,12}$	0.02691	0.16052	-0.02007	-0.21514	0.02159	-0.00352	-0.06223	-0.0666
$\kappa_{7,13}$	-0.04424	-0.01448	0.06478	0.03153	-0.03844	0.00624	0.02825	-0.0083
$\kappa_{7,14}$	-0.00084	-0.00731	-0.00086	0.00989	0.00028	-0.00116	0.00449	0.00266
$\kappa_{7,15}$	-0.00872	-0.02452	0.00646	0.03527	0.00209	-0.001	0.0137	0.00633
$\kappa_{7,16}$	0.00505	-0.00294	-0.01139	0.00475	0.01198	-0.00342	0.00325	0.00074
$\kappa_{7,17}$	0.00045	-0.00093	-0.00164	0.00127	0.00137	-0.00105	0.00101	-3e-05
$\kappa_{7,18}$	0.02548	0.08897	-0.01899	-0.13554	0.02265	-0.02281	-0.04198	-0.06865
$\kappa_{8,8}$	-0.02644	0.03529	0.02792	-0.05051	-0.06322	-0.04474	0.04285	-0.0732
$\kappa_{8,9}$	-0.00228	0.00232	0.00537	-0.00484	-0.00559	-0.00024	0.00052	-0.00072
$\kappa_{8,10}$	0.00169	0.00752	-0.00107	-0.01276	0.00166	0.00049	-0.0042	-0.0031
$\kappa_{8,11}$	0.0001	-0.00247	-0.00038	0.00393	0.00037	-0.00027	0.00133	0.00137
$\kappa_{8,12}$	0.00209	-0.01439	-0.00734	0.01655	0.00801	3e-05	-0.00247	7e-05
$\kappa_{8,13}$	0.00073	0.02879	0.01688	-0.03977	-0.00424	-0.0063	-0.01153	-0.02727
$\kappa_{8,14}$	7e-05	2e-05	-3e-05	-5e-05	0.00026	-0.00029	0.00031	-0.0
$\kappa_{8,15}$	-0.00146	0.00592	0.00511	-0.00911	-0.00425	0.00087	-0.0031	-0.00202
$\kappa_{8,16}$	-0.00046	-0.00072	0.00248	-0.0001	-0.00298	-0.00059	-0.00015	-0.00113
$\kappa_{8,17}$	-1e-05	-0.00019	0.00037	0.00014	-0.00051	0.00021	-1e-05	0.00025
$\kappa_{8,18}$	-0.01529	-0.04461	0.00483	0.06926	0.00158	-0.0129	0.03238	0.02372

$\kappa_{9,9}$	-0.01455	0.04993	0.06706	-0.07678	-0.11592	-0.03448	0.01645	-0.04336
$\kappa_{9,10}$	-0.00213	0.01467	0.01153	-0.02291	-0.00959	0.00468	-0.00332	0.00098
$\kappa_{9,11}$	0.00279	0.00155	-0.00625	-0.0041	0.00728	0.00022	-0.00056	-0.00123
$\kappa_{9,12}$	0.00098	0.00283	-0.00201	-0.00338	-0.00012	0.00115	-0.00258	0.00061
$\kappa_{9,13}$	-0.001	0.00158	0.00297	-0.00122	0.00029	-0.00054	0.00095	-0.00152
$\kappa_{9,14}$	0.00191	4e-05	-0.00185	0.00086	0.0012	-0.00015	0.00016	0.00059
$\kappa_{9,15}$	-0.00205	0.00146	0.00587	-0.00209	-0.0019	-0.00179	0.00084	-0.00347
$\kappa_{9,16}$	0.00733	0.01901	-0.02422	-0.03773	0.01071	-0.0002	0.00208	0.0127
$\kappa_{9,17}$	-0.00212	0.00854	0.01234	-0.01311	-0.01013	0.00166	-0.0036	-0.00499
$\kappa_{9,18}$	-0.00027	-0.00106	0.0008	0.00131	0.001	-0.0017	0.00159	-0.001
$\kappa_{10,10}$	-0.00426	-0.03274	0.03071	0.04956	-0.09533	-0.03343	0.02567	-0.02899
$\kappa_{10,11}$	0.00026	0.00647	0.00649	-0.00981	-0.00332	0.00101	-0.00113	-0.0042
$\kappa_{10,12}$	0.00281	0.01831	-0.00424	-0.02723	0.00305	-0.00327	-0.0064	-0.01076
$\kappa_{10,13}$	-0.00188	-0.00691	0.00164	0.00973	-0.0019	0.00043	0.00204	0.00184
$\kappa_{10,14}$	-0.00206	-0.00253	0.00122	0.00405	-0.0017	0.00092	0.00149	0.0028
$\kappa_{10,15}$	0.00048	0.00174	-0.00965	-0.00872	0.00539	0.00256	0.00779	0.01545
$\kappa_{10,16}$	0.00095	0.00443	-0.00306	-0.00656	0.00243	-2e-05	0.00013	0.00125
$\kappa_{10,17}$	0.00423	0.00679	-0.00615	-0.01213	0.0064	-0.00117	-0.00092	-0.00293
$\kappa_{10,18}$	0.00132	0.04066	0.003	-0.05937	-0.00199	0.003	-0.02428	-0.02325
$\kappa_{11,11}$	0.06423	0.01461	0.1375	-0.02062	-0.07293	0.02053	-0.00321	-0.01383
$\kappa_{11,12}$	0.00122	-0.00224	-0.00266	0.0039	0.00152	-3e-05	0.00143	0.00228
$\kappa_{11,13}$	0.00188	-0.00474	-0.00276	0.00685	0.00265	-0.00143	0.00237	0.00101
$\kappa_{11,14}$	0.00375	0.00719	0.0036	-0.01337	0.00422	0.00149	-0.0029	-0.00272
$\kappa_{11,15}$	-0.00564	0.00664	0.0158	-0.01258	-0.01746	0.00312	-0.00466	-0.00106
$\kappa_{11,16}$	-0.0046	-0.00789	0.0213	0.01662	-0.02157	0.00446	0.00347	0.00519
$\kappa_{11,17}$	0.00044	-0.00093	0.0004	0.00139	8e-05	0.00071	-0.00019	0.00084

$\kappa_{11,18}$	-0.00057	0.0034	0.00104	-0.00469	-0.00109	0.00049	-0.00263	-0.002
$\kappa_{12,12}$	-0.03762	-0.00832	0.1567	0.02127	-0.07881	0.00277	0.01771	-0.04584
$\kappa_{12,13}$	0.01365	0.04977	0.00304	-0.0665	0.0098	-0.00872	-0.02285	-0.03425
$\kappa_{12,14}$	0.00186	0.00519	-0.00171	-0.00751	0.00203	-0.00158	-0.00164	-0.00375
$\kappa_{12,15}$	0.00066	0.02509	0.00824	-0.03527	-0.00656	0.00248	-0.00914	-0.00868
$\kappa_{12,16}$	-0.00297	-0.00408	0.00981	0.00361	-0.00486	0.00039	0.00404	-0.00034
$\kappa_{12,17}$	6e-05	-0.0008	0.00089	0.00116	-9e-05	0.00053	-7e-05	0.00056
$\kappa_{12,18}$	0.01527	-0.03453	-0.01747	0.04918	0.0164	-0.00203	0.00159	0.00397
$\kappa_{13,13}$	-0.01914	-0.06304	0.03593	0.07527	0.00226	-0.04571	0.09811	0.02094
$\kappa_{13,14}$	0.00052	-0.00691	-0.00149	0.00955	0.00131	-0.00096	0.00362	0.00311
$\kappa_{13,15}$	-0.0065	0.00312	0.01364	-0.00131	-0.0072	0.0013	0.00147	-0.00569
$\kappa_{13,16}$	0.00138	-0.00125	-0.00496	0.00136	0.00379	-0.002	0.00142	-0.00066
$\kappa_{13,17}$	0.00017	0.00045	-0.00078	-0.00059	0.00021	-0.00043	6e-05	-0.00051
$\kappa_{13,18}$	-0.02574	0.05232	0.01712	-0.07594	-0.02766	0.01126	-0.03891	-0.03472
$\kappa_{14,14}$	-0.02882	0.01266	0.13513	-0.01201	-0.06513	0.00218	0.02684	-0.02398
$\kappa_{14,15}$	0.003	-0.00131	-0.00102	0.00062	0.00257	0.00138	-0.00113	-0.00026
$\kappa_{14,16}$	0.00017	0.00151	0.00103	-0.00295	-0.00066	0.00064	-0.00024	0.00045
$\kappa_{14,17}$	0.00983	0.0001	-0.00083	-0.00065	-0.00172	0.01158	-0.01093	0.00758
$\kappa_{14,18}$	0.00187	0.00753	-0.00252	-0.01086	0.00243	-0.00078	-0.00392	-0.0048
$\kappa_{15,15}$	-0.02621	0.00817	0.09532	-0.02625	-0.12928	-0.01886	0.04828	-0.0035
$\kappa_{15,16}$	0.00218	-0.00105	-0.00803	0.00229	0.00625	-0.00138	-0.0021	-0.00214
$\kappa_{15,17}$	-0.00096	-8e-05	0.00142	0.00014	-0.00103	-0.00044	-0.00035	-0.00108
$\kappa_{15,18}$	0.00311	0.03324	-9e-05	-0.04668	0.00057	-0.00036	-0.01858	-0.02057
$\kappa_{16,16}$	-0.01792	0.01248	0.06742	-0.00678	-0.11648	-0.02833	-0.00849	-0.06277
$\kappa_{16,17}$	-0.00109	0.00514	0.00529	-0.00844	-0.00444	0.00042	-0.00204	-0.00275
$\kappa_{16,18}$	-0.00082	0.01502	0.00296	-0.0229	-0.00275	0.00027	-0.00889	-0.01084

$\kappa_{17,17}$	-0.03431	0.02153	0.02682	-0.02987	-0.06773	-0.04961	0.05491	-0.06372
$\kappa_{17,18}$	0.00014	-0.00026	0.00034	0.0005	0.00044	0.0006	-0.00042	0.00029
$\kappa_{18,18}$	-0.13788	-0.05148	0.0352	0.07802	-0.0575	-0.02815	0.07764	0.01548

mode	v126	v128	v129	v130	v133	v137	v139	v140
$\kappa_{0,0}$	0.0	0.0	0.0	0.0	-0.0	0.0	0.0	0.0
$\kappa_{0,1}$	0.00655	-0.00518	0.001	0.00879	0.00017	-0.00281	0.00108	0.00583
$\kappa_{0,2}$	0.00246	0.00503	0.00245	0.00604	-0.00676	0.00656	0.00809	-0.0016
$\kappa_{0,3}$	-0.0011	0.00096	-0.15707	0.00601	-0.00329	-0.00407	-0.02017	-0.37165
$\kappa_{0,4}$	9e-05	1e-05	-4e-05	0.00016	-2e-05	-0.00025	4e-05	0.00016
$\kappa_{0,5}$	0.04114	0.01777	-0.00569	-0.00342	-0.02042	-0.03899	0.01338	-0.01457
$\kappa_{0,6}$	-0.00052	-0.00026	-0.00016	2e-05	-0.00059	-0.0005	0.00032	-0.00051
$\kappa_{0,7}$	0.04234	-0.00113	0.00616	0.00716	-0.01823	0.0211	-0.00358	-0.00632
$\kappa_{0,8}$	-0.00871	0.01873	-0.00015	-0.00036	-0.00251	-0.01171	-0.00612	0.0031
$\kappa_{0,9}$	-0.00067	0.00029	-8e-05	-0.00097	0.00118	7e-05	0.00052	9e-05
$\kappa_{0,10}$	-0.00041	-0.00052	0.00034	-0.00231	0.00181	-0.00016	-0.00047	0.00079
$\kappa_{0,11}$	0.00149	-0.00557	-0.003	0.00041	-0.00224	0.00788	0.01054	-0.0041
$\kappa_{0,12}$	0.00335	0.04431	-0.00359	0.00163	-0.02634	-0.0826	-0.02785	-0.00661
$\kappa_{0,13}$	0.00052	0.00694	-0.00165	0.00194	0.04329	0.0653	0.02195	-0.01173
$\kappa_{0,14}$	0.00012	-0.00261	-0.00045	-7e-05	-0.00023	0.00296	0.00262	-0.00092
$\kappa_{0,15}$	-0.00248	0.00591	0.00275	-0.00614	0.00137	0.00267	0.00085	0.00308
$\kappa_{0,16}$	-0.00402	-0.00537	0.00029	0.00223	-0.00121	-0.00062	-0.00323	0.00082
$\kappa_{0,17}$	-0.00033	0.00014	0.00025	-0.00086	0.00065	-0.00031	-0.00038	0.00048
$\kappa_{0,18}$	0.01469	-0.02258	-0.00549	-0.00047	0.02341	0.00168	0.00903	-0.01671
$\kappa_{1,1}$	0.02614	0.00178	-0.00372	0.1138	-0.09972	-0.00991	0.08092	0.00144
$\kappa_{1,2}$	-0.00871	0.00573	0.00336	0.00562	-0.00496	0.0066	-0.00809	0.01622
$\kappa_{1,3}$	-0.00011	-0.00165	-0.00244	0.00106	0.00325	0.00247	-0.00349	-0.0014

$\kappa_{1,4}$	-0.00038	-0.00051	-0.00074	-0.0008	0.00034	0.00049	-8e-05	-0.00156
$\kappa_{1,5}$	0.00049	0.00042	0.00038	0.00286	-0.00261	-0.00201	0.00146	-0.00066
$\kappa_{1,6}$	-0.00463	-0.00079	-0.00437	-0.0035	-0.00307	-0.00374	0.00191	-0.01421
$\kappa_{1,7}$	-0.00281	0.00176	0.00138	0.00279	-0.00054	-0.00101	0.00024	0.00402
$\kappa_{1,8}$	0.00047	-0.00091	0.00045	0.00021	0.00061	0.00125	0.00058	0.00064
$\kappa_{1,9}$	0.0033	-0.00356	-0.0009	-0.00023	0.00189	0.00289	0.00267	-0.00332
$\kappa_{1,10}$	-0.01959	0.00308	-0.00243	0.00118	0.00777	0.01544	0.00671	-0.00253
$\kappa_{1,11}$	0.0226	-0.02699	-0.00705	-0.00839	-0.00141	-0.0233	-0.0206	-0.01252
$\kappa_{1,12}$	-0.0012	0.00238	-0.00043	-0.00223	0.001	0.00043	-0.00031	-0.00162
$\kappa_{1,13}$	-0.00201	0.00272	-0.00102	-0.00197	-0.00219	-0.01154	-0.00406	0.00057
$\kappa_{1,14}$	-0.00046	-0.00481	0.00264	-0.00019	0.00082	0.00494	0.00411	0.00422
$\kappa_{1,15}$	-0.04255	-0.00978	-0.00917	-0.01281	0.01873	-0.01082	0.009	-0.01239
$\kappa_{1,16}$	-0.0079	0.00373	0.00138	0.00098	0.00218	0.0014	-0.00126	0.00347
$\kappa_{1,17}$	-5e-05	0.00017	0.00085	-5e-05	-0.00071	-0.00014	0.00033	0.00186
$\kappa_{1,18}$	0.00061	0.00145	0.00081	-0.00019	-0.00084	0.00049	0.0001	0.00225
$\kappa_{2,2}$	0.01996	-0.0209	-0.01884	0.09657	-0.05086	0.0414	0.15726	-0.06874
$\kappa_{2,3}$	0.00084	0.00202	-0.00733	-0.00176	0.00058	0.00062	-0.00015	-0.01691
$\kappa_{2,4}$	0.00137	0.00231	0.0036	0.00159	-0.00168	-0.00428	-0.00018	0.00663
$\kappa_{2,5}$	0.00031	-0.00102	0.00516	-0.00167	0.00161	0.00535	0.00019	0.00933
$\kappa_{2,6}$	-0.00121	-8e-05	-0.0011	0.00012	0.00013	-0.00021	2e-05	-0.00322
$\kappa_{2,7}$	0.00271	0.00127	0.00159	0.00046	-0.00058	0.00174	0.0004	0.00133
$\kappa_{2,8}$	0.00068	-0.00042	-0.00027	-0.00027	0.00044	0.00063	-0.00054	-0.0001
$\kappa_{2,9}$	-0.00616	0.01024	0.00212	-0.00587	-0.01317	-0.0266	-0.01301	0.00656
$\kappa_{2,10}$	-0.00295	0.00047	0.00053	-0.00087	0.00028	0.00152	0.00098	0.00228
$\kappa_{2,11}$	-0.01699	-0.04512	-0.00535	0.00368	0.00816	0.03479	0.03869	-0.01559
$\kappa_{2,12}$	0.00167	0.00048	0.0016	0.00267	-0.00027	0.00466	0.00265	0.0044

$\kappa_{2,13}$	0.00033	0.00054	0.00062	-0.00117	-0.00109	-0.00085	-0.00037	0.00022
$\kappa_{2,14}$	-0.00294	0.00242	-0.00266	0.00322	-0.00053	0.00225	0.00031	-0.00496
$\kappa_{2,15}$	-0.00557	-0.00356	-0.00066	-0.00297	0.00321	-0.00018	0.00356	-8e-05
$\kappa_{2,16}$	0.03522	-0.01237	-0.00283	0.00691	-0.00408	-0.00914	-0.00366	-0.00286
$\kappa_{2,17}$	0.00025	0.00075	0.00063	0.00046	0.00037	-0.00012	-0.001	0.00141
$\kappa_{2,18}$	0.00013	0.00108	-0.00059	0.00262	-0.00193	0.00146	0.00147	-0.00169
$\kappa_{3,3}$	0.02652	-0.00992	-0.02989	0.15017	0.03169	0.04393	-0.00147	-0.07463
$\kappa_{3,4}$	0.00018	0.0001	-2e-05	0.00077	-0.00019	0.0002	0.00038	-0.0001
$\kappa_{3,5}$	0.00142	0.00244	0.0512	-0.00349	-0.00116	-0.00183	0.00538	0.11609
$\kappa_{3,6}$	0.0002	-0.0002	-0.00181	-0.00076	-0.00097	-0.00027	-0.00031	-0.00396
$\kappa_{3,7}$	0.00265	-0.00655	0.02435	-0.00512	0.0058	0.01125	0.0049	0.05447
$\kappa_{3,8}$	-0.00135	0.00137	0.00259	0.00019	-0.00066	-0.00175	0.00019	0.0059
$\kappa_{3,9}$	0.00052	7e-05	0.0047	-0.00023	0.00051	0.00024	-6e-05	0.01078
$\kappa_{3,10}$	-0.00072	0.00093	-0.00118	-0.00039	-0.00086	-0.00062	-0.00022	-0.00326
$\kappa_{3,11}$	-5e-05	-0.0004	0.02093	-0.0006	-0.00227	0.00187	0.00259	0.04866
$\kappa_{3,12}$	0.00069	0.00147	0.02261	-0.00132	-0.00048	-0.00167	0.00429	0.0528
$\kappa_{3,13}$	0.0045	-0.00332	-0.01618	0.0032	0.00279	0.00491	0.0016	-0.03679
$\kappa_{3,14}$	-0.00016	-6e-05	-0.01083	-0.00101	0.00208	-0.00026	-0.00137	-0.0246
$\kappa_{3,15}$	-0.00099	0.00054	0.00587	0.00071	-0.00156	-0.00046	0.00226	0.01239
$\kappa_{3,16}$	-0.00106	-0.00098	-0.00683	0.00013	-0.00058	0.00098	0.00116	-0.01682
$\kappa_{3,17}$	-4e-05	-3e-05	-0.00097	0.00016	6e-05	-0.00019	-2e-05	-0.00207
$\kappa_{3,18}$	0.00063	-0.00425	-0.0017	0.00165	0.00154	0.00361	0.00278	-0.00278
$\kappa_{4,4}$	0.04193	0.02533	-0.03221	0.17001	0.02336	0.02907	-0.03952	-0.06826
$\kappa_{4,5}$	-0.00029	-0.00104	0.00052	-0.00061	-0.00011	7e-05	0.00016	0.00112
$\kappa_{4,6}$	-0.00103	0.00079	0.00104	9e-05	0.00084	0.00234	0.00034	0.00267
$\kappa_{4,7}$	-6e-05	-0.00048	-0.00015	0.00012	0.00023	0.00031	1e-05	-0.00039

$\kappa_{4,8}$	-0.00021	-0.00022	5e-05	-0.00019	-0.00022	-0.0003	0.0	0.00033
$\kappa_{4,9}$	-0.00093	-0.00046	-0.00127	-0.00525	-0.003	-0.00077	-0.00073	-0.00229
$\kappa_{4,10}$	-0.00015	0.00016	-4e-05	-0.00122	-0.00056	4e-05	0.00052	-0.00027
$\kappa_{4,11}$	0.00053	0.00019	0.00076	0.00149	-0.0005	-0.00127	0.00021	0.00216
$\kappa_{4,12}$	-0.0	0.00037	-0.00021	-0.00017	-6e-05	0.00033	0.0003	-0.00068
$\kappa_{4,13}$	-0.00011	0.00025	-0.0001	9e-05	0.0003	-0.00036	-0.00036	3e-05
$\kappa_{4,14}$	0.00759	0.02511	0.00303	0.00183	-0.01667	-0.03273	-0.01239	0.00939
$\kappa_{4,15}$	8e-05	8e-05	0.00039	-0.00016	-0.00071	-0.00041	0.00012	0.00104
$\kappa_{4,16}$	0.00062	0.00115	0.01232	0.0032	0.0015	0.00512	0.00293	0.0244
$\kappa_{4,17}$	-0.00144	-0.00064	0.00094	-0.00038	0.00178	0.0059	0.00228	0.00179
$\kappa_{4,18}$	-0.00013	0.00027	0.00012	0.00041	-0.00045	9e-05	-6e-05	0.00038
$\kappa_{5,5}$	0.0757	0.01394	-0.0206	0.00074	0.00235	0.01248	0.0047	-0.06191
$\kappa_{5,6}$	-0.00024	0.00041	0.00061	-0.0003	5e-05	-0.00099	-0.00048	0.00125
$\kappa_{5,7}$	0.04497	-0.01362	0.00329	0.00183	0.02732	0.08746	0.0115	0.01294
$\kappa_{5,8}$	-0.00985	-0.00814	0.00058	0.00019	0.00033	0.00175	0.00498	0.00051
$\kappa_{5,9}$	-0.00037	-0.00301	-0.00146	0.00297	-0.00238	-0.00293	-0.00154	-0.00224
$\kappa_{5,10}$	0.00167	0.0097	-0.00057	-0.00045	0.00242	0.00671	-0.0005	-0.00022
$\kappa_{5,11}$	0.00114	0.00337	-0.00014	0.00078	-0.00101	0.00058	-0.00239	0.00046
$\kappa_{5,12}$	-0.068	-0.04265	-0.00467	0.0022	-0.01306	-0.04689	0.03693	-0.01561
$\kappa_{5,13}$	0.0242	-0.01961	-0.00532	0.00219	0.01303	0.04242	0.01761	-0.01405
$\kappa_{5,14}$	0.00046	0.00058	0.00043	0.00223	0.00153	-0.00219	-0.00243	0.00162
$\kappa_{5,15}$	0.00572	-0.00523	-0.00189	0.00534	0.00054	0.01223	0.00578	-0.00226
$\kappa_{5,16}$	-0.00266	0.00861	0.00446	0.00142	-0.0014	0.0062	0.00684	0.0058
$\kappa_{5,17}$	-0.00028	0.00011	0.00032	0.00039	-0.00015	-0.00101	-0.00035	0.00069
$\kappa_{5,18}$	-0.00663	0.01789	0.00434	0.00613	-0.0171	-0.02452	-0.02054	0.01825
$\kappa_{6,6}$	0.04251	-0.04276	-0.03342	0.17017	0.01861	0.0458	-0.02271	-0.07591

$\kappa_{6,7}$	0.0005	-0.00272	-0.00076	0.00012	0.0005	0.00109	0.00094	-0.00214
$\kappa_{6,8}$	-9e-05	0.00054	5e-05	-5e-05	9e-05	-0.00034	-0.00023	0.0002
$\kappa_{6,9}$	2e-05	-0.00078	0.00096	0.00011	0.00016	0.00107	0.00041	0.00199
$\kappa_{6,10}$	-0.00076	0.00016	-0.00734	-0.00172	-7e-05	-0.00242	-0.00134	-0.01486
$\kappa_{6,11}$	0.00168	-0.00087	-0.00078	0.00026	-0.00032	-0.00038	-0.00032	-0.00045
$\kappa_{6,12}$	-0.0002	0.00044	-0.00021	-0.0002	0.00018	-0.00012	8e-05	-0.00059
$\kappa_{6,13}$	6e-05	0.00011	-0.00024	-0.00017	3e-05	-0.0001	-9e-05	-0.00042
$\kappa_{6,14}$	-0.00552	0.01777	-0.00825	-0.00095	0.02308	0.02652	0.01277	-0.02345
$\kappa_{6,15}$	0.00174	0.00021	-2e-05	0.00453	0.0044	0.00021	0.00179	-0.00013
$\kappa_{6,16}$	0.00014	0.00039	0.00032	3e-05	-0.00104	-0.00113	0.0004	0.0006
$\kappa_{6,17}$	0.00115	0.00063	-0.0006	0.00025	-0.00272	-0.00641	-0.00225	-0.00108
$\kappa_{6,18}$	1e-05	0.00174	0.00037	0.00029	-0.00069	-0.00048	-0.00098	0.00126
$\kappa_{7,7}$	0.18429	-0.16667	-0.01915	0.01143	0.02653	0.11847	0.03854	-0.06149
$\kappa_{7,8}$	-0.00165	0.01631	-0.0008	0.00449	-0.02219	-0.02775	-0.00913	0.0005
$\kappa_{7,9}$	0.00283	-0.00598	0.00028	0.001	-0.0008	0.00296	0.00093	2e-05
$\kappa_{7,10}$	-0.00181	0.01738	0.00153	-0.00434	-0.00244	-0.00276	-0.00053	0.0012
$\kappa_{7,11}$	-0.00157	0.00054	0.00077	0.0003	-0.00082	-0.00296	-0.00136	0.0029
$\kappa_{7,12}$	-0.01351	-0.09346	-0.00479	0.00103	0.01269	-0.00404	0.00575	-0.0065
$\kappa_{7,13}$	0.0489	-0.03084	-0.00529	-0.00152	-0.00982	0.04542	0.00383	-0.02219
$\kappa_{7,14}$	0.00025	0.00184	0.00032	0.00148	-0.00028	-0.0	-0.0013	-0.00028
$\kappa_{7,15}$	0.00608	0.00642	-0.001	-0.01131	0.00199	-0.00482	-0.0098	-0.0017
$\kappa_{7,16}$	-0.00433	-0.00262	1e-05	-0.00156	0.00211	-0.00349	-0.00174	0.00023
$\kappa_{7,17}$	-0.00042	0.00028	5e-05	0.00045	-0.00076	-0.00088	-8e-05	0.00012
$\kappa_{7,18}$	0.00177	-0.0326	-0.00919	-0.00573	0.01317	-0.02696	0.0112	-0.02897
$\kappa_{8,8}$	0.06319	-0.01103	-0.01595	-0.00789	-0.00448	0.02935	-0.04693	-0.03989
$\kappa_{8,9}$	0.003	0.00031	0.00171	-0.0015	-0.00508	-0.00175	-0.00205	0.00505

$\kappa_{8,10}$	0.00025	-0.00195	-0.00162	0.0008	0.00114	0.00031	0.00172	-0.00343
$\kappa_{8,11}$	-0.0003	0.00103	0.00097	0.0002	-0.00036	-0.00066	-0.00021	0.00206
$\kappa_{8,12}$	-0.00569	0.03816	-0.00028	-0.00026	0.00184	-0.00249	0.0044	-0.00303
$\kappa_{8,13}$	0.01593	0.00762	-0.00046	-0.00137	-0.00021	-0.02288	-0.01098	-0.00264
$\kappa_{8,14}$	-3e-05	2e-05	1e-05	0.00103	-0.00053	-0.00085	-0.00011	0.00028
$\kappa_{8,15}$	0.00196	-0.00134	-0.00164	0.0033	-0.00416	-0.00265	0.0023	-0.00359
$\kappa_{8,16}$	0.00159	0.00253	-0.0007	0.00056	-0.00197	-0.00051	0.00042	-0.00165
$\kappa_{8,17}$	3e-05	6e-05	9e-05	0.00091	-0.00052	-0.0007	0.00035	0.0003
$\kappa_{8,18}$	0.00246	0.01409	0.00403	0.00633	-0.04282	-0.04905	-0.01284	0.01432
$\kappa_{9,9}$	0.03933	0.03164	-0.00754	0.11087	-0.11292	-0.03185	0.0565	-0.00578
$\kappa_{9,10}$	0.00198	0.01591	0.00192	0.00488	-0.00301	-0.00261	-0.01454	0.0111
$\kappa_{9,11}$	-0.00099	-0.00294	-0.00104	-0.00349	0.00341	0.00072	0.00156	-0.00354
$\kappa_{9,12}$	-0.00159	-0.00298	0.00075	0.00096	0.00084	0.00296	0.00348	0.00162
$\kappa_{9,13}$	0.00108	-0.00278	-0.00173	-0.00168	-0.00088	-0.00316	-0.00364	-0.00327
$\kappa_{9,14}$	-0.00038	-0.00385	-0.00015	-0.00225	0.0056	0.00211	-0.0038	-0.00041
$\kappa_{9,15}$	0.00324	-0.00138	-0.00019	0.00157	-0.00439	-0.0104	-0.00188	-0.00157
$\kappa_{9,16}$	-0.0143	0.01034	0.00417	0.00302	-0.00643	0.00969	-0.01336	0.02043
$\kappa_{9,17}$	0.00657	0.00105	0.00398	0.00343	0.00059	0.00312	-0.00204	0.01173
$\kappa_{9,18}$	0.00078	0.00185	-0.00196	-0.00292	-0.00174	-0.00746	-0.00409	-0.00281
$\kappa_{10,10}$	0.02213	-0.04873	-0.01663	0.0917	-0.06926	0.04745	0.14182	-0.06343
$\kappa_{10,11}$	0.00519	0.00315	0.00025	-0.00276	-0.00038	-0.00296	-0.0053	-0.00039
$\kappa_{10,12}$	-0.00077	-0.00341	-0.00016	0.00099	0.00027	1e-05	0.00057	-0.00065
$\kappa_{10,13}$	0.00176	0.00118	-0.00055	-0.00102	0.0012	0.00307	0.00158	-0.00293
$\kappa_{10,14}$	0.00059	-0.00036	0.00118	-0.00236	-0.00236	0.0011	0.00092	0.00265
$\kappa_{10,15}$	-0.0079	0.00689	0.00242	0.00373	-0.00744	0.00396	-0.01009	0.01396
$\kappa_{10,16}$	-0.00215	0.00213	0.00165	-0.00123	0.0001	0.00014	-0.00404	0.00569

$\kappa_{10,17}$	-0.00157	-0.00014	-0.001	-0.00133	0.00066	0.00242	-0.00096	-0.00071
$\kappa_{10,18}$	0.00173	-0.01725	-0.00239	-0.0026	0.00978	0.00983	0.0127	-0.01062
$\kappa_{11,11}$	0.0495	-0.01752	-0.01361	0.09983	-0.03263	-0.04335	0.0632	-0.03547
$\kappa_{11,12}$	-0.00138	-0.00045	0.00045	0.00206	0.0014	0.0031	0.00091	0.00054
$\kappa_{11,13}$	-0.00101	0.00211	-0.00097	0.00056	0.00079	0.0003	-0.00062	-0.00209
$\kappa_{11,14}$	0.00111	-0.0045	-0.00273	-0.00023	0.00318	-0.00107	1e-05	-0.00082
$\kappa_{11,15}$	0.00718	0.00601	0.00073	0.00446	0.00035	0.00548	-0.00111	-0.00207
$\kappa_{11,16}$	0.00906	-0.00856	-0.00048	0.00249	-0.0014	0.00389	-0.00524	0.00319
$\kappa_{11,17}$	-0.00029	0.00011	-0.00084	-0.00043	0.0005	0.00028	0.00073	-0.00165
$\kappa_{11,18}$	0.00017	-0.0015	-0.00022	-0.00028	0.00092	0.00075	0.00149	-0.0017
$\kappa_{12,12}$	0.06808	0.04434	-0.01496	0.01028	-0.05952	-0.12107	-0.01801	-0.03976
$\kappa_{12,13}$	0.00156	-0.01422	-0.00468	-0.00425	-8e-05	-0.02918	0.00367	-0.01461
$\kappa_{12,14}$	-0.0001	-0.00175	-0.00046	0.00175	0.00025	-0.00328	-0.00119	-0.00047
$\kappa_{12,15}$	0.00306	-0.0123	-0.00102	0.0028	-0.00033	-0.00127	-0.00193	-0.00012
$\kappa_{12,16}$	0.00584	0.0033	-0.00039	-0.00115	-0.00229	-0.0072	-0.00726	-0.00096
$\kappa_{12,17}$	6e-05	0.00059	-0.00019	-0.00086	0.00091	-0.0004	-0.00072	-0.00027
$\kappa_{12,18}$	-0.01252	0.03425	-0.00142	-0.00261	0.01534	0.00086	0.01201	-0.00837
$\kappa_{13,13}$	0.02487	0.03089	0.00117	0.02028	-0.12235	-0.17657	-0.05593	0.01811
$\kappa_{13,14}$	-0.0008	0.00167	-0.00057	0.00041	-0.00069	-0.00117	-0.00045	-0.00214
$\kappa_{13,15}$	0.00937	-0.01004	-0.00326	0.00193	-0.00048	0.00369	-0.0003	-0.00856
$\kappa_{13,16}$	-0.00151	-7e-05	0.00015	-0.00149	0.00031	0.00029	0.00033	-0.00119
$\kappa_{13,17}$	-4e-05	-3e-05	-0.0002	0.00038	-0.00066	5e-05	0.00053	-0.0005
$\kappa_{13,18}$	0.01125	0.00048	-0.00407	-0.00123	0.01251	0.04152	0.02161	-0.01634
$\kappa_{14,14}$	0.05725	-0.00659	-0.0246	0.19001	-0.06674	-0.11189	-0.01626	-0.05296
$\kappa_{14,15}$	-0.00045	0.00225	-0.00086	-0.0079	0.00733	0.00299	-0.00161	-0.00041
$\kappa_{14,16}$	0.00029	-0.00133	0.00118	0.00597	-0.00018	0.0035	0.0025	0.00466

$\kappa_{14,17}$	-0.00927	0.00019	-0.00081	-0.00132	0.01647	0.01517	0.00905	-0.00257
$\kappa_{14,18}$	-0.001	-0.00301	-0.00129	0.00284	0.00222	0.00106	0.00225	-0.00346
$\kappa_{15,15}$	0.04619	-0.02478	-0.0071	0.11421	-0.12654	-0.02753	0.06688	-0.01328
$\kappa_{15,16}$	-0.0025	-0.00065	0.00057	-0.00531	0.00485	0.00499	0.0047	-0.00058
$\kappa_{15,17}$	0.00084	0.00112	0.00073	0.00037	-0.00062	-0.00293	-0.0007	0.0013
$\kappa_{15,18}$	0.00015	-0.0171	-0.00259	-0.00071	0.00621	0.00431	0.01097	-0.01014
$\kappa_{16,16}$	0.03555	0.02686	-0.01234	0.10411	-0.0675	0.02437	0.12654	-0.04539
$\kappa_{16,17}$	0.0027	0.00031	0.00111	0.00199	-0.00098	-0.00028	-0.00097	0.00354
$\kappa_{16,18}$	0.00214	-0.00648	-0.00268	0.00089	0.00348	0.00394	0.00548	-0.00873
$\kappa_{17,17}$	0.06205	-0.00751	-0.03149	0.16937	0.0013	0.01911	-0.05973	-0.06692
$\kappa_{17,18}$	-0.00028	-0.00015	0.00012	-0.00127	0.00113	-0.00047	-0.00057	0.00036
$\kappa_{18,18}$	0.01749	0.00983	-0.00478	0.03711	-0.16815	0.02708	0.05268	-0.00877

S5 Quantum Dynamical Simulations

The quantum dynamical simulations were performed based on the Hamiltonian described above. In this section, we provide further details of the simulations.

S5.1 Computational Details

The simulation without vibrational modes was performed by solving the time-dependent Schrödinger equation of the shifted diabatic electronic Hamiltonian using the matrix exponential with a timestep of 0.01 fs.

The quantum dynamical simulations including vibrational modes were performed using the multilayer formulation of the multiconfiguration time-dependent Hartree method (ML-MCTDH).[7–13] The ML-MCTDH tree used for the full model $\hat{\mathcal{H}}$ is presented in Figure S5. The single particle functions (spf) were set to $s = [19, 150, 100, 67, 45, 30]$, where each index

corresponds to the number of spf for each branch of the ML-tree per layer. The primitive basis functions (pbf) were selected according to the largest dimensionless reorganization energy $\max(\frac{\kappa_m^2}{2\omega_m^2})$ and scaled by the factor 30 with a minimum of 30 pbf. This number of spf and pbf was selected after careful convergence tests, which ensure that the results for the population dynamics remain largely unchanged upon further increase, while pushing our simulations to the limits of what is computationally feasible. For the simulations with $\hat{\mathcal{H}}_{\text{inter}}$ and $\hat{\mathcal{H}}_{\text{intra}}$ the spf of the first layer were set to the number of electronic states accordingly.

The ML-trees of the calculations incorporating model $\hat{\mathcal{H}}_1$, $\hat{\mathcal{H}}_2$, and $\hat{\mathcal{H}}_3$ are depicted in Figure S6, Figure S7, and Figure S8, respectively.

S5.2 Dynamics of the Intramolecular Singlet Fission in the DADB Molecule

Figure S9 shows the time evolution of the population of the electronic states involved in the intramolecular SF in the donor molecule DADB for the full model $\hat{\mathcal{H}}$, model $\hat{\mathcal{H}}_1$, model $\hat{\mathcal{H}}_2$, and model $\hat{\mathcal{H}}_3$. The intramolecular SF involves the LE states in the DADB molecule, the CT_D group, and the ME_D group. In all four models after the initial excitation of the higher-lying LE state in the DADB molecule, the population of the CT_D states immediately increases followed by the population of the ME_D group in the first 2 fs. After this initial process, the CT_D and ME_D group oscillate in antiphase indicating a strong coupling between the two. When including vibrational modes with frequencies 100 cm^{-1} $< \omega_m <$ 1000 cm^{-1} (see Figure S9 (b)) differences in the dynamics can be observed after 2 fs. The maximum population of the ME_D is only reached after 20 fs. In Figure S9 (c) and (d) the dynamics undergo a similar behavior compared to the full model in Figure S9 (a), while Figure S9 (d) shows slightly larger oscillations. This is because the simulation includes only the five ring-breathing vibrational modes of the tetrachlorophenylene bridge in DADB (with 1000 cm^{-1} $<$ $\omega_m <$ 1200 cm^{-1}).

Ultrafast intramolecular SF occurs in all four models. The results show that the vi-

brational modes only influence the details of the dynamics of the process after the initial population of the ME_D, which is in line with the electronic dynamics presented in Figure 7 of the manuscript.

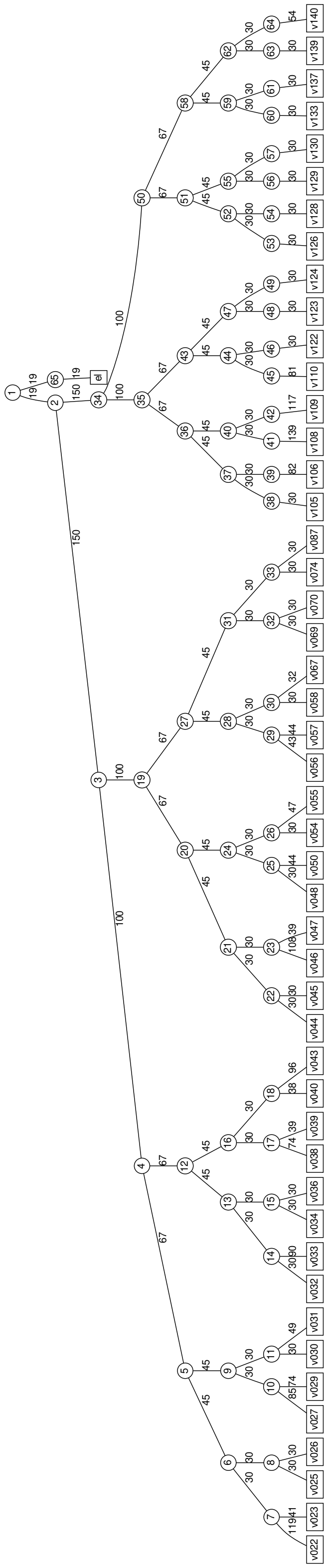

Figure S5: ML-MCTDH tree used for the quantum dynamical simulation incorporating the full model $\hat{\mathcal{H}}$.

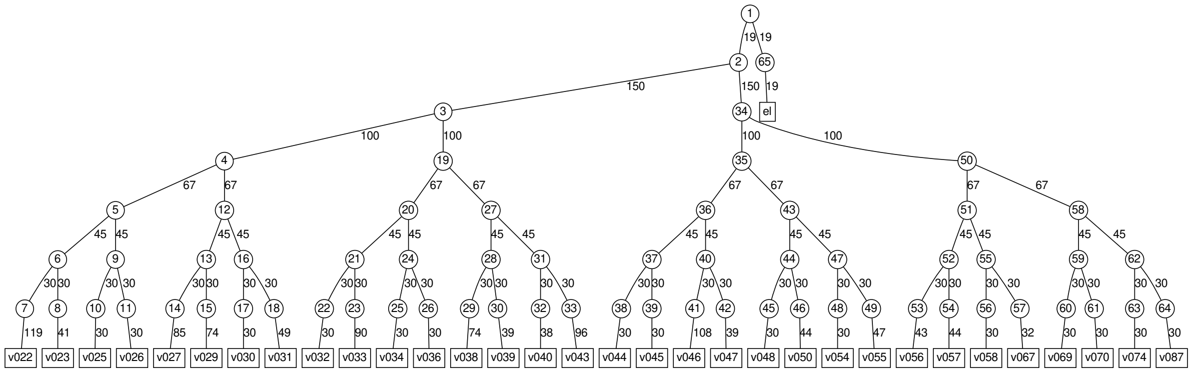

Figure S6: ML-MCTDH tree used for the quantum dynamical simulation incorporating the model $\hat{\mathcal{H}}_1$.

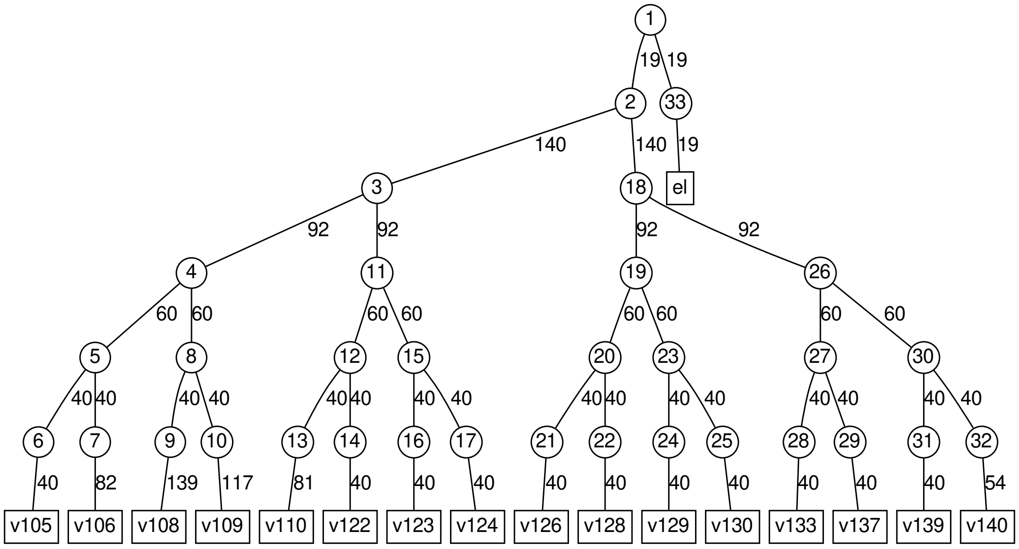

Figure S7: ML-MCTDH tree used for the quantum dynamical simulation incorporating the model $\hat{\mathcal{H}}_2$.

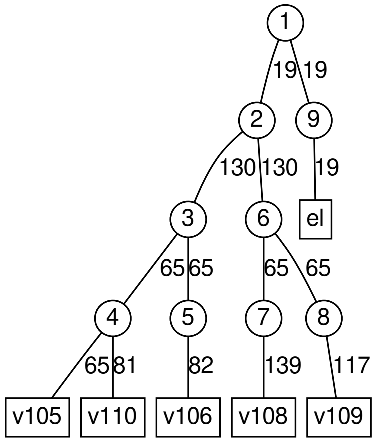

Figure S8: ML-MCTDH tree used for the quantum dynamical simulation incorporating the model $\hat{\mathcal{H}}_3$.

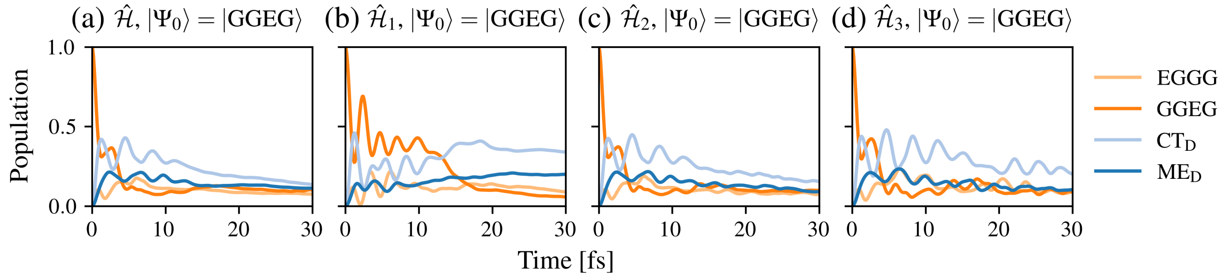

Figure S9: Time evolution of the population of the electronic states involved in the intramolecular singlet fission in the DADB molecule with initial state $|\Psi_0\rangle = |\mathrm{GGEG}\rangle$ of the full model $\hat{\mathcal{H}}$, model $\hat{\mathcal{H}}_1$, model $\hat{\mathcal{H}}_2$, and model $\hat{\mathcal{H}}_3$.

References

(1) Dunning, T. H. Gaussian basis sets for use in correlated molecular calculations. I. The atoms boron through neon and hydrogen. *J. Chem. Phys.* **1989**, *90*, 1007–1023.

(2) Granovsky, A. A. Extended multi-configuration quasi-degenerate perturbation theory: The new approach to multi-state multi-reference perturbation theory. *J. Chem. Phys.* **2011**, *134*, 214113.

(3) Barca, G. M. J. et al. Recent developments in the general atomic and molecular electronic structure system. *J. Chem. Phys.* **2020**, *152*, 154102.

(4) Nakamura, H.; Truhlar, D. G. The direct calculation of diabatic states based on configurational uniformity. *J. Chem. Phys.* **2001**, *115*, 10353–10372.

(5) Nakamura, H.; Truhlar, D. G. Direct diabatization of electronic states by the fourfold way. II. Dynamical correlation and rearrangement processes. *J. Chem. Phys.* **2002**, *117*, 5576–5593.

(6) Nakamura, H.; Truhlar, D. G. Extension of the fourfold way for calculation of global diabatic potential energy surfaces of complex, multiarrangement, non-Born–Oppenheimer systems: Application to HNCO(S,S1). *J. Chem. Phys.* **2003**, *118*, 6816–6829.

(7) Meyer, H.-D.; Manthe, U.; Cederbaum, L. The multi-configurational time-dependent Hartree approach. *Chem. Phys. Lett.* **1990**, *165*, 73–78.

(8) Manthe, U.; Meyer, H.-D.; Cederbaum, L. S. Wave-packet dynamics within the multiconfiguration Hartree framework: General aspects and application to NOCl. *J. Chem. Phys.* **1992**, *97*, 3199–3213.

(9) Beck, M. H.; Jäckle, A.; Worth, G. A.; Meyer, H.-D. The multiconfiguration time-dependent Hartree (MCTDH) method: a highly efficient algorithm for propagating wavepackets. *Phys. Rep.* **2000**, *324*, 1–105.

(10) Wang, H.; Thoss, M. Multilayer formulation of the multiconfiguration time-dependent Hartree theory. *J. Chem. Phys.* **2003**, *119*, 1289–1299.

(11) Manthe, U. A multilayer multiconfigurational time-dependent Hartree approach for quantum dynamics on general potential energy surfaces. *J. Chem. Phys.* **2008**, *128*, 164116.

(12) Vendrell, O.; Meyer, H.-D. Multilayer multiconfiguration time-dependent Hartree method: Implementation and applications to a Henon–Heiles Hamiltonian and to pyrazine. *J. Chem. Phys.* **2011**, *134*, 044135.

(13) Worth, G. A.; Beck, M. H.; Jäckle, A.; Meyer, H.-D. The MCTDH Package, Version 8.2, (2000). H.-D. Meyer, Version 8.3 (2002), Version 8.4 (2007). O. Vendrell and H.-D. Meyer Version 8.5 (2013). Versions 8.5 and 8.6 contain the ML-MCTDH algorithm. See http://mctdh.uni-hd.de for a description of the Heidelberg MCTDH package. Used version: 8.6.4 (2023).
